# A New Online Computational Biology Curriculum

**DOI:** 10.1371/journal.pcbi.1003662

**Published:** 2014-06-12

**Authors:** David B. Searls

**Affiliations:** Independent Consultant, Philadelphia, Pennsylvania, United States of America; Ontario Institute for Cancer Research, Canada

## Abstract

A recent proliferation of Massive Open Online Courses (MOOCs) and other web-based educational resources has greatly increased the potential for effective self-study in many fields. This article introduces a catalog of several hundred free video courses of potential interest to those wishing to expand their knowledge of bioinformatics and computational biology. The courses are organized into eleven subject areas modeled on university departments and are accompanied by commentary and career advice.

## Introduction

Less than two years ago, the author published an online bioinformatics curriculum in this journal and made the claim (with some important caveats) that a sufficient number and variety of free video courses had made their way to the web that it was possible to obtain a reasonably comprehensive bioinformatics education on one's laptop [1]. In that compilation of courseware, only a few entries originated from the then-nascent Coursera platform (https://www.coursera.org), and none came from its academic competitor edX (https://www.edx.org). In the intervening time, these platforms and several others have fairly exploded with new content, such that on the order of a thousand courses are now available online from over a hundred academic institutions. That fact alone justifies an update to the curriculum and a reassessment of the viability of online education in this field.

To begin with the latter, it should first be acknowledged that MOOCs are controversial in many regards. This article will not attempt to review or comment on the generic issues beyond making a few general observations in the Conclusion below. It is the opinion of the author that MOOCs are indeed a valuable resource even if they are not a magic bullet. The general limitations as regards bioinformatics were discussed in the previous article [1] and in a companion piece giving practical advice to online learners [2] and need not be recapitulated here. Certainly the sizeable increases in content that have occurred in the interim have improved the prospects, yet they have also raised the bar, and it is now clearer than ever where the gaps and shortcomings are in the available curriculum. Specific instances will be commented upon in the appropriate contexts below. One general observation is that the MOOC universe provides good coverage at the introductory level and plenty of specialized “elective” courses, but comprehensive intermediate and advanced courses are thin on the ground in some areas, including biology. For example, as of this writing there are no MOOCs dedicated to the subject of structural biology, which is surprising given the importance of visualization in the field and the availability of excellent online resources. Nevertheless, the sizeable expansion of courses available, particularly in allied fields such as neurosciences and evolutionary biology, has been deemed sufficient to widen the scope of this edition to encompass the more expansive term “computational biology” as opposed to “bioinformatics” (for those who consider the distinction important).

MOOCs continue to generate large enrollments, at least initially, and these numbers together with anecdotal evidence from course discussion forums indicate active interest in online education among a certain population. This evidently extends to the readership of PLOS Computational Biology, judging from article-level metrics for the original curriculum [1], which has now attracted over 60,000 views and as of a year after its appearance was the 12th most viewed article in the history of the journal (per data available from http://www.ploscompbiol.org/static/almInfo).

Those same metrics reveal high levels of interest in skills improvement and career advice, a conclusion that is based upon the popularity of the “Ten Simple Rules” series, which accounts for six of the ten most viewed articles. The topics of these six include giving talks [3], making posters [4], getting published [5], obtaining grants [6], selecting postdoctoral positions [7], and choosing between career paths in academia and industry [8] (the final article also having been written by this author). To better accommodate these interests, the current edition of the curriculum has been extended in two ways. First, articles have been included (at the end of the catalog) that specifically address nonscientific skills likely to be useful in career development. Second, the commentaries on individual courses now include not only evaluations of their content but also career advice and other personal comments tied to that subject and based on the experiences of the author, both in the classroom (real and virtual) and over the course of a varied career in bioinformatics. These features are described in more detail below.

## Description

As before, the curriculum is offered in the form of a virtual course catalog divided into the departments of an imagined university. One consequence of the avalanche of new courses is that the catalog is several times longer than the previous edition, making it intractable for the article format used previously. Thus, the new catalog is provided as Supporting Information to this article in the form of a PDF attachment (Catalog S1). To assist in using the document, the PDF has a listing of courses by department attached as metadata (as well as at the head of the text), which can be opened as a navigation bar in typical PDF readers like Adobe Reader (View>Navigation Panels>Bookmarks) or Apple Preview (View>Table of Contents).

Previously, the virtual university had only four departments: Biology, Mathematics, Computer Science, and Other. Another consequence of the greatly increased number of courses is that many additional departments have been founded in this edition in specialized areas more or less relevant to computational biology. Since courses in the original basic science departments may also bear upon one or more of the new specialized departments, and vice versa, such courses are simply cross listed between departments, as is common practice in real universities. This allows courses to be grouped together in “majors,” which in this edition substitute for the “tracks” that were described in the previous paper [1]. Those tracks had more to do with career paths than with subject area concentrations and are still relevant but, as an orthogonal feature, would be difficult to depict across many departments. It is hoped that appropriate tracks in the new material will be self-evident given the track descriptions in the previous catalog and the course commentaries in this one.

In this edition of the catalog, each listing takes one of three forms: Courses, Current Topics, or Seminars. Courses, which comprise the majority of entries, are based on discrete university offerings, which as before are required to be video-based and free of charge. Most of these are MOOCs, which is to say that they run on a set schedule with interactive features and have graded assessments; nearly all MOOCs listed are from Coursera or edX. In the catalog these are distinguished from other learning resources, such as those that simply offer recordings of lectures for completely independent self-study, though it should be noted that the term MOOC is sometimes applied more broadly and that MOOCs may also be made available in archived form for offline self-study.

Course listings in this catalog are essentially of the same type and form as in the previous edition, except that course names are uniformatized and no longer need correspond to the exact names given by instructors, which can often be quirky, vague, or overlong. The new canonical subject names should make the catalog easier to navigate, and even if the recommended course is not a perfect fit to the label, the course chosen is the one judged to be the closest fit to what is deemed a suitable topic in the curriculum. Any variations are explained in the course commentaries.

Course entries are headed by the instructor's name (omitting titles), his or her institution, the original title of the course, and in parentheses, the platform, date of latest offering (or TBA to indicate a date yet to be arranged), and the URL. This is followed by an indented, italicized, and quoted paragraph that is excerpted from the course description offered by the provider. This in turn is followed by this author's commentary on the course, a list of prerequisites (if any), alternative course offerings, and suggested follow-ups, all as in the previous catalog [1]. In addition, this edition of the catalog identifies the primary textbook used in the course together with suggested alternatives.

The course listings labeled as Current Topics in a given subject are not formal university courses but generally meetings, workshops, or seminar series in which the videos are talks by a number of investigators in the form of tutorials or descriptions of their current research. For our purposes, this simulates a typical upper-level university course that exposes students to the most recent research in a certain area in a coordinated fashion.

The course listings labeled as Seminars in a given subject are similar in spirit but are much less coordinated since they are drawn from individual online seminar videos from different sources. Unlike Current Topics, which are talks from a single focused meeting or source, Seminars comprise some 10–20 individual talks selected by the author to be representative of a particular subfield but certainly not to be comprehensive or even especially balanced. In a few cases, Seminars are compiled so as to help compensate for the absence or weakness of some upper-level course in the curriculum, such as the Developmental Biology Seminars. In other cases, they provide exposure to prominent scientists, recent research trends, and/or broader perspectives. Each collection of Seminars on a specified topic is aggregated as a YouTube playlist, to which a link is provided.

As was noted for the preceding publication [1], this article is necessarily an opinion piece, since universities tend to disagree on optimal curricula and the author's personal judgments are involved in selecting the most appropriate course (sometimes from among many) for a particular topic. Moreover, the commentaries attached to each course offer opinions on the importance of the subject to a computational biology education, as well as the quality of instruction for that particular course. For better or worse, this is the “value-added” provided by the author, beyond a simple compilation of URLs.

Even beyond this, the current edition of this catalog is still more unabashedly personal in two new regards. First, the author has road tested most of the recommended courses, enrolling in up to a dozen at a time on a continuous basis in the case of MOOCs. Many of the MOOCs deemed most worthwhile were actually completed for a grade. This was sufficient to be one of the top 50 students in terms of the number of completed courses on Coursera as of mid-2013, and as of this writing, the author has completed for a grade a total of 60 courses on Coursera and 12 on edX. Of these, 50 were chosen as primary recommended courses in a given subject area. While there are about 200 subject area listings in the catalog, many of these are Current Topics courses and Seminars, and still more of the remaining courses are not MOOCs as we have defined them, so that in fact about 40% of the MOOCs receiving primary recommendations were completed for a grade.

For the completed courses only, the course listings have an additional section called Evaluation, comprising a table with the following entries: (1) Course Level, which is instantiated as either Introductory, Intermediate, or Advanced, representing an assessment of the true level of difficulty of the material, regardless of the official course description; (2) Hours per Week, representing the estimated effort required for all course activities, which again may vary from that suggested by the instructors; (3) Course Grade, representing numeric evaluations on a scale of 100 of several aspects of the course including (a) Lectures, based on their content, style, and production values, (b) Homework, based on the effectiveness of exercises (graded or not), ancillary materials, assigned reading, or any other activities beyond the course lectures, (c) Assessment, based on the quality of the quizzes and exams in terms of whether they are sufficiently challenging, reflective of actual accomplishment, and learning experiences in themselves, and (d) Overall, based on the combination of all factors, including intangibles; (4) Student Grade, representing the grade the author himself received in the course, in the interest of fairness and full disclosure; and finally (5) Curve, indicating both the passing grade of the course, as required for a certificate, and the grade required for an “A” or for a certificate with distinction, where applicable. The Student Grade combined with the Curve may be useful to the reader in assessing such things as the level of difficulty of the course, the stringency of the grading, and the credibility of the author's judgments. Note that because courses that were followed to completion by the author and selected as primary recommendations tended to be of higher quality, the Course Grades have an inbuilt bias toward the high end of the scale.

The second personalized element in this edition is the occasional inclusion of paragraphs with a heading of Personal Note in the course commentaries. These are autobiographical annotations related to courses or their subject matter, reflecting the author's own experience and included as a matter of interest or to elaborate on why certain courses may be useful in a career in computational biology. (The author's own career is briefly described in the previous catalog [1] as well as in the Personal Notes themselves.) The reader is, of course, free to discount or disregard these highly individualized annotations.

## Conclusion

As noted in the Introduction, MOOCs are controversial in many regards and certainly not universally acclaimed. Many of the criticisms, however, have been or are being addressed to some degree. Identification verification technologies have lent more legitimacy to assessment and certification. Particularly with courses that are well staffed with teaching assistants, the availability of individual attention can be surprisingly high, and often the discussion forums are a satisfactory substitute for direct student-teacher interaction. Curricula are becoming better coordinated by virtue of the release of entire packages of courses in a given area of study by a single institution, in what Coursera calls “Specializations” and edX calls “XSeries.” Increasingly imaginative approaches are being taken by individual courses to designing student activities so as to better simulate classroom, laboratory, or field experiences, though much remains to be done in this arena. Assessment, which remains very uneven in quality and effectiveness, may in fact not be much worse than in real courses and at least has the potential to benefit greatly from across-the-board quality-control measures, technology improvements, and data-mining approaches afforded by the nature of MOOCs.

What must be weighed against the hurdles facing MOOCs and online learning is the tremendous variety and increasing depth of courses available. As can be seen in the catalog, there is often a choice among competing courses for popular topics, and while some more advanced subjects may not be offered exactly when they are wanted, sometimes the material is available offline, and there are enough courses that several of interest are almost certainly running at any given time. Having now taken comparable numbers of real and virtual courses, the author is firmly of the opinion that both types are normally distributed with regards to quality and that the distributions largely overlap. The subpar MOOCs can be sampled and discarded at very little cost, and the best MOOCs are very good indeed.

## Supporting Information

Catalog S1 **Course catalog.**
(PDF)
