# Supplementary material for: A New Online Computational Biology Curriculum
Source: PLoS Comput Biol. 2014 Jun 12;10(6):e1003662. doi: 10.1371/journal.pcbi.1003662 (PMC4055417; doi:10.1371/journal.pcbi.1003662)
Supplement: Catalog S1 — Course catalog. (PDF) [file pcbi.1003662.s001.pdf]

Computational Biology Online Course Catalog

Supplement to:

Searls DB (2014) A new online computational biology curriculum.
PLoS Comput Biol 10(6): e1003662.
doi: 10.1371/journal.pcbi.1003662

© David B. Searls 2014. This is an open-access article distributed under the terms of the Creative Commons Attribution License, which permits unrestricted use, distribution, and reproduction in any medium, provided the original author and source are credited.

See the full publication (cited above) for a detailed description of the catalog format, including Evaluation categories.

For references to the previous catalog, see: Searls DB (2012) An online bioinformatics curriculum. PLoS Comput Biol 8(9): e1002632. doi: 10.1371/journal.pcbi.1002632

A 💻 icon indicates a MOOC (Coursera, edX, or Udacity), and a ✓ means that it was completed and evaluated by the author in this catalog.

Note that links to edX courses may change with each iteration of the course. If inactive links or archived courses are encountered, simply search for the same course by subject and/or school at https://www.edx.org/course-list. The same may be true of Stanford courses, which can be found at https://class.stanford.edu/courses. The Berkeley Webcast site is occasionally flaky, so that all links to courses there also include alternative links to the YouTube versions.

Course Listings by Department

Mathematics Department ... **1**
- Calculus 💻 ... 1
- Differential Equations ... 1
- Linear Algebra 💻✓ ... 2
- Abstract Algebra ... 3
- Real Analysis ... 3
- Complex Analysis 💻✓ ... 4
- Functional Analysis 💻 ... 4
- Topology ... 5
- Discrete Mathematics ... 5
- Combinatorics ... 6
- Probability 💻✓ ... 6
- Statistics 💻 ... 7
- Logic 💻✓ ... 7
- Theory of Computation 💻✓ ... 8
- Linear Programming 💻 ... 9
- Numerical Methods 💻 ... 10
- Dynamical Systems ... 10
- Signal Processing 💻 ... 11
- Information Theory 💻 ... 12
- Game Theory 💻✓ ... 12
- Graph Theory ... 13

Computer Science Department ... **15**
- Introduction to Computer Science 💻 ... 15
- Data Structures 💻 ... 16
- Machine Structures 💻 ... 17
- Algorithms 💻✓ ... 18
- Operating Systems 💻 ... 19
- Software Engineering 💻 ... 19
- Human-Computer Interaction 💻 ... 20
- Web Development 💻 ... 21
- Mobile Computing 💻 ... 21
- Embedded Systems 💻 ... 22
- Scientific Computing 💻 ... 22
- Parallel Computing 💻 ... 23
- Programming Languages 💻✓ ... 24
- Functional Programming 💻✓ ... 24
- Databases 💻 ... 25
- Computer Networks 💻✓ ... 25
- Artificial Intelligence 💻✓ ... 26
- Machine Learning 💻✓ ... 27
- Natural Language Processing 💻 ... 27
- Robotics 💻 ... 28
- Discrete Optimization 💻 ... 28
- Computer Graphics 💻 ... 29
- Image Processing 💻 ... 29
- Computer Science Seminars ... 30

Data Science Department ... **31**
- Introduction to Data Science 💻✓ ... 31
- Data Science 💻 ... 31
- Data Analytics 💻 ... 32
- Big Data 💻✓ ... 32
- Databases 💻 ... 33
- Metadata 💻 ... 34
- Statistics 💻 ... 34
- Mathematical Biostatistics 💻✓ ... 35
- Statistical Programming 💻✓ ... 35
- Machine Learning 💻✓ ... 36
- Statistical Learning 💻✓ ... 37
- Graphical Models 💻✓ ... 38
- Neural Networks 💻 ... 38
- Natural Language Processing 💻 ... 39
- Computer Graphics 💻 ... 39
- Visualization ... 39
- Current Topics in Data Science ... 39
- Current Topics in Big Data ... 40
- Current Topics in Machine Learning ... 40
- Current Topics in Visualization ... 40
- Data Science Seminars ... 41

Chemistry Department ... **42**
- Introduction to Chemistry 💻 ... 42
- General Chemistry 💻 ... 42
- Laboratory Chemistry ... 43
- Introduction to Physical Chemistry 💻 ... 43
- Thermodynamics 💻✓ ... 43
- Solid State Chemistry 💻 ... 44
- Crystallography ... 45
- Introductory Organic Chemistry 💻 ... 46
- Intermediate Organic Chemistry 💻 ... 46
- Biochemistry ... 47
- Chemical Biology 💻 ... 47
- Medicinal Chemistry 💻✓ ... 47
- Analytical Chemistry 💻 ... 48
- Nanotechnology 💻 ... 48
- Cheminformatics ... 48
- Computational Chemistry ... 49

Biology Department ... **50**
- Introduction to Biology 💻 ... 50
- Laboratory Biology ... 51
- Biochemistry ... 52
- Genetics 💻 ... 53
- Molecular Biology ... 54
- Cell Biology ... 55
- Developmental Biology ... 55
- Evolutionary Biology ... 56
- Cancer Biology ... 56
- Genomics 💻✓ ... 57
- Proteomics ... 58
- Epigenetics 💻✓ ... 58
- Immunology 💻 ... 59
- Virology 💻✓ ... 59
- Apoptosis 💻✓ ... 60
- Current Topics in Genomics ... 61
- Current Topics in Immunology ... 61
- Developmental Biology Seminars ... 61
- Cell Signaling Seminars ... 61
- Gene Regulation Seminars ... 61
- Structural Biology Seminars ... 61

Computational Biology Department 62
Bioinformatics Methods 62
Computational Biology.................................... 63
Computational Genomics 63
Computational Evolution 65
Computational Ecology 65
Computational Neuroscience 65
Sequence Analysis Algorithms 66
Genomics Algorithms 66
Quantitative Biology 66
Mathematical Biology 67
Mathematical Ecology 68
Current Topics in Bioinformatics Methods 68
Current Topics in Computational Biology 69
Current Topics in Computational Genomics 69
Computational Biology Seminars....................... 69
Evolutionary Biology Department 70
Introduction to Evolution 70
Darwinism 70
Evolutionary Biology 71
Evolutionary Genetics 71
Computational Evolution 72
Introduction to Paleontology 72
Introduction to Paleobiology 72
Dinosaur Paleobiology 73
Paleoanthropology 73
Paleopathology 74
Astrobiology 75
Current Topics in Paleoanthropology 75
Current Topics in Cognitive Evolution................ 76
Evolutionary Biology Seminars.......................... 76
Systems Biology Department 77
Physiology 77
Introduction to Systems Biology 77
Mathematical Systems Biology 78
Quantitative Systems Biology 78
Experimental Systems Biology 79
Introduction to Networks 79
Network Analysis 80
Network Biology 80
Introduction to Modeling 81
General Systems Modeling 81
Biological Systems Modeling 82
Neuronal Modeling 82
Graphical Models 82
Dynamical Systems 82
Systems Biology Applications 82
Current Topics in Systems Biology 83
Current Topics in Synthetic Biology 83
Current Topics in Network Analysis 83
Systems Biology Seminars................................ 84
Neurosciences Department 85
Introduction to Psychology 85
Introduction to Neurosciences 85
Introduction to Medical Neurobiology 86
Neuroanatomy and Neurophysiology 86
Clinical Neurology 87
Neuropharmacology 88
Systems Neuroscience 88
Circuits and Electronics 88
Computational Neuroscience 89
Neuronal Modeling 89
Behavioral Biology..90
Neuroeconomics 90
Consciousness 91
Current Topics in Cognitive Evolution 91
Neurosciences Seminars 92
Translational Sciences Department 93
Introduction to Drug Discovery 93
Pharmacology 93
Neuropharmacology 94
Medicinal Chemistry 94
Cheminformatics 94
Health Informatics 95
Infectious Disease Dynamics 95
Epidemiology and Biostatistics 96
Clinical Trials 97
Clinical Data Management 97
Biomedical Engineering.....................................98
Biomedical Imaging 98
Genomic Medicine 99
Biomedical Ethics 99
Healthcare Policy 100
Healthcare Entrepreneurship 100
Current Topics in Genomic Medicine 101
Drug Discovery Seminars.................................101
Translational Sciences Seminars 101
Humanities Department............................. 102
Introduction to Philosophy 102
Logic 103
Ontology...103
Ethics 103
Philosophy of Mind 104
Philosophy and Science 105
Philosophy and Mathematics 106
Linguistics 106
Literature 107
History 108
Archaeology 109
Architecture 110
Art 110
Music 111
Economics 112
Business 112
Writing Skills 113
Speaking Skills 114
Teaching Skills 114
Creative Skills 115
Argumentation Skills 115
Leadership Skills 115
Entrepreneurial Skills 116
Humanities Seminars.......................................117

Mathematics Department

The Mathematics Department has been filled out with a number of MOOCs and other online courseware, compared to the previous catalog, and now comprises a fairly respectable undergraduate curriculum in this subject area. The upper-level courses will likely be of interest only to mathematical biologists and to computational biologists at the theoretical end of the spectrum, although these also include courses that have mathematical content but are usually offered in engineering departments. All else being equal, courses are recommended that have a computational slant, and many of these actually involve programming.

Calculus

Bart Snapp, Jim Fowler, and Jenny George, Ohio State University "Calculus One" and "Calculus Two: Sequences and Series" (Coursera, Winter/Spring 2014: https://www.coursera.org/course/calc1 and https://www.coursera.org/course/sequence)

> [Part 1] *"Functions and Limits… Infinity and Continuity… Basics of Derivatives… Curve Sketching, The Product Rule and Quotient Rule… The Chain Rule… The Derivatives of Trigonometric Functions and their Inverses… Applications of Differentiation… Optimization… Linear Approximation… Antiderivatives… Integrals… The Fundamental Theorem of Calculus… Techniques of Integration… Applications of Integration."* [Part 2] *"Sequences… Series… Convergence tests… Alternating series… Power series… Taylor series"*

Commentary. For those who need a thorough review of calculus there is no shortage of online courses, and this one is among the most basic, yet thorough. It is available through Coursera or direct from Ohio State (https://mooculus.osu.edu).

Textbook. The course makes use of its own very snazzy free online textbook, which is perhaps its main attraction (https://mooculus.osu.edu/textbook/mooculus.pdf). Also, Gilbert Strang of the Massachusetts Institute of Technology has made his textbook available online (http://ocw.mit.edu/resources/res-18-001-calculus-online-textbook-spring-2005/textbook). Finally, the *Funny Little Calculus Text* by Robert Ghrist (a supplement to the first Alternative course listed below) simply must be seen to be believed (http://books.google.com/books?id=HbbeRHUozJcC).

Alternatives. "Calculus: Single Variable" by the University of Pennsylvania's Robert Ghrist on Coursera is a more advanced and rapid-fire treatment (https://www.coursera.org/course/calcsing). This could be productively combined with the "Massively Multivariable Open Online Calculus Course" by Jim Fowler (again) and Steve Gubkin of Ohio State University (https://www.coursera.org/course/m2o2c2), which introduces a fair amount of linear algebra and may be a more useful treatment for computational biologists who will be dealing with its high dimensional datasets. Another means of reviewing the subject in a fresh way is by studying it in an entirely different context, and Coursera's "Mathematical Methods for Quantitative Finance" by Kjell Konis of the University of Washington may be of interest to many (https://www.coursera.org/course/mathematicalmethods). It quickly ranges over calculus and a lot more, using many examples from finance and providing a good idea of what "quants" do on Wall Street and in other financial centers. See the previous catalog for additional alternatives.

Differential Equations

Arthur Mattuck, Massachusetts Institute of Technology "Differential Equations" (MIT OpenCourseWare, Fall 2011: http://ocw.mit.edu/courses/mathematics/18-03sc-differential-equations-fall-2011)

"The laws of nature are expressed as differential equations. Scientists and engineers must know how to model the world in terms of differential equations, and how to solve those equations and interpret the solutions. This course focuses on the equations and techniques most useful in science and engineering."

Commentary. This carryover from the previous catalog is not a MOOC but has many features of one in its current modularized form, including visual demonstrations, and the lecturer is excellent. The topic is essential in mathematical biology and in the modeling of biological systems.

Personal Note. There is a trend nowadays to try to get by with graphical and numerical solutions to differential equations, and these are indeed useful skills. Having survived the classroom version of this very course, though, the author is of the curmudgeonly opinion that one should be exposed to analytical approaches such as this before becoming completely dependent on computers and qualitative methods.

Prerequisites. Calculus.

Textbook. Highly polished albeit quite terse course notes are provided in an episodic fashion. A consolidated and rather more chatty set of notes is available from Cornell's Robert Terrell (http://www.math.cornell.edu/~bterrell/dn.pdf).

Alternatives. Udacity's "Differential Equations in Action" by Jörn Loviscach focuses on numerical rather than analytical solutions (see Personal Note), using Python to solve a variety of problems including epidemic spread (https://www.udacity.com/course/cs222). Also see the previous catalog.

Linear Algebra

Philip Klein, Brown University "Coding the Matrix: Linear Algebra through Computer Science Applications" (Coursera, Summer 2013: https://www.coursera.org/course/matrix)

"In this class, you will learn the concepts and methods of linear algebra, and how to use them to think about problems arising in computer science. You will write small programs in the programming language Python to implement basic matrix and vector functionality and algorithms, and use these to process real-world data to achieve such tasks as: two-dimensional graphics transformations, face morphing, face detection, image transformations such as blurring and edge detection, image perspective removal, audio and image compression, searching within an image or an audio clip, classification of tumors as malignant or benign, integer factorization, error-correcting codes, secret-sharing, network layout, document classification, and computing Pagerank (Google's ranking method)."

Commentary. Of the several fine courses available for this essential topic, this one was chosen primarily for its heavy emphasis on computation (using Python) at a fundamental level, as well as on applications. However, the course stops just short of eigenvectors and their applications, an important topic for computational biology, so some students may prefer to choose from the alternatives below, particularly if they have a more theoretical bent. There are hints of a sequel to this course that would address this shortcoming.

Evaluation.

Course Level	*Hours per Week*	*Course Grade*				*Student Grade*	*Curve*
		Lectures	*Homework*	*Assessment*	*Overall*		*Pass/"A"*
Introductory	8	88	93	92	90	99.8	60/90

Prerequisites. Some mathematical sophistication and programming experience.

Textbook. The instructor has an optional textbook [1] with a companion web site that has data and support code, and even auto-grading (http://codingthematrix.com), essentially providing an offline alternative for this MOOC. Another free textbook is *A First Course in Linear Algebra* by Robert Beezer of the University of Puget Sound (http://linear.pugetsound.edu).

Alternatives. The course by Gilbert Strang of the Massachusetts Institute of Technology, recommended in the previous catalog, is still a very strong contender, and has well-done MOOC-like features (http://ocw.mit.edu/courses/mathematics/18-06sc-linear-algebra-fall-2011). Also, edX now has "Linear Algebra—Foundations to Frontiers" by Maggie Myers and Robert van de Geijn of the University of Texas at Austin (https://www.edx.org/course/utaustinx/utaustinx-ut-5-01x-linear-algebra-1162). See the previous catalog for additional recommendations.

Going Further. Again, see the previous catalog for recommendations for going further in applied math of this nature, and in particular consider the advanced courses by the estimable Gilbert Strang.

Abstract Algebra

Benedict Gross, Harvard University "Abstract Algebra" (Harvard Extension School, Fall 2003: http://www.extension.harvard.edu/open-learning-initiative/abstract-algebra)

> *"Algebra is the language of modern mathematics. This course introduces students to that language through a study of groups, group actions, vector spaces, linear algebra, and the theory of fields."*

Commentary. Algebraic structures are important in theoretical computer science and may be of interest to certain computational biologists for that reason, though most will get what they need from the less formal and/or more computational courses in this Department. Though not a MOOC, the course web page has (handwritten) notes and problem sets. If the lecture links cause any problems, they are also available on YouTube (http://www.youtube.com/playlist?list=PLA58AC5CABC1321A3).

Prerequisites. Differential Equations, Linear Algebra, and experience writing proofs.

Textbook. *Algebra* by Michael Artin [2]. A classic text, but expensive. For a free online alternative, try the one by Thomas Judson of the Stephen F. Austin University (http://abstract.ups.edu/download.html).

Real Analysis

Francis Su, Harvey Mudd College "Real Analysis I" (Harvey Mudd College, Spring 2010: http://analysisyawp.blogspot.com)

> *"This course is a rigorous analysis of the real numbers, as well as an introduction to writing and communicating mathematics well. Topics will include: construction of the real numbers, fields, complex numbers, topology of the reals, metric spaces, careful treatment of sequences and series, functions of real numbers, continuity, compactness, connectedness, differentiation, and the mean value theorem, with an introduction to sequences of functions."*

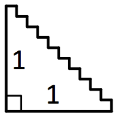

Commentary. Real analysis is calculus on steroids, and many computational biologists will neither need nor desire it. Here is a quick diagnostic: Given a right triangle with sides adjacent to the right angle of length one, approximate its hypotenuse by a staircase of *n* steps. Each step thus has both height and width $1/n$, totaling $2/n$. Taking the limit as *n* goes to infinity of *n* steps times $2/n$, as you are used to doing in calculus, you get a total length of 2. Now if it really, really bothers you that you don't get the square root of 2, then this course may be for you. The instructor is wonderful, but it is not a MOOC and tragically the resolution of the videos is marginal. For that reason, this course should be considered by only the most enthusiastic math-minded students, until a MOOC appears to supplant it.

Prerequisites. Discrete Mathematics, according to the Harvey Mudd catalog, but the syllabus suggests that Abstract Algebra or general mathematical sophistication would also serve.

Textbook. *Principles of Mathematical Analysis* by Walter Rudin [3]. Inspired by this standard text, Jiri Lebl of the University of Illinois, Urbana-Champaign recently made available his own very nice free

online textbook (http://www.jirka.org/ra/realanal.pdf). At the next level, Jeff Viaclovsky's graduate course in "Measure and Integration" at the Massachusetts Institute of Technology has a solid set of notes (http://ocw.mit.edu/courses/mathematics/18-125-measure-and-integration-fall-2003/lecture-notes).

Alternatives. A slightly more basic version of "Real Analysis" (also not a MOOC) is taught by S.H. Kulkami of the Indian Institute of Technology, Madras (http://nptel.ac.in/courses/111106053).

Going Further. "Measure and Integration" by Inder Rana of the Indian Institute of Technology, Bombay (http://nptel.ac.in/courses/111101005) is a graduate level continuation of real analysis, again in non-MOOC form.

Complex Analysis 💻✓

Petra Bonfert-Taylor, Wesleyan University "Analysis of a Complex Kind" (Coursera, Fall 2013: https://www.coursera.org/course/complexanalysis)

> *"Introduction to complex numbers, their geometry and algebra, working with complex numbers... The Mandelbrot set, Julia sets, a famous outstanding conjecture, history of complex numbers, sequences of complex numbers and convergence, complex functions... Complex differentiation and the Cauchy-Riemann equations... Conformal mappings, Möbius transformations and the Riemann mapping theorem... Complex integration, Cauchy-Goursat theorem, Cauchy integral formula, Liouville's Theorem, maximum principle, fundamental theorem of algebra... Power series representation of analytic functions, singularities, the Riemann zeta function, Riemann hypothesis, relation to prime numbers."*

Commentary. The complex plane forms the foundation of engineering math and dynamical systems, and this course is a good way to become comfortable living in it and to review the underpinnings of calculus at the same time. Ironically, it is no more difficult than the Real Analysis course above, though no cakewalk. Of particular interest are conformal mappings, which have neuroinformatics applications in imaging and representations such as flattening of brain cortex maps [4]. The course is very effectively taught with a combination of well-designed slides and annotations from the instructor.

Evaluation.

Course Level	*Hours per Week*	*Course Grade*				*Student Grade*	*Curve*
		Lectures	*Homework*	*Assessment*	*Overall*		*Pass/"A"*
Intermediate	8	94	93	92	95	91.9	65/—

Prerequisites. Calculus.

Textbook. None is required, but the instructor recommends a free online set of lecture notes from Matthias Beck, Gerald Marchesi, Dennis Pixton, and Lucas Sabalka of the State University of New York, Binghamton and San Francisco State (http://math.sfsu.edu/beck/complex.html).

Functional Analysis 💻

John Cagnol and Anna Rozanova-Pierrat, École Centrale Paris "An Introduction to Functional Analysis" (Coursera, Winter 2014: https://www.coursera.org/course/functionalanalysis)

> *"Functional analysis is the branch of mathematics dealing with spaces of functions. It is a valuable tool in theoretical mathematics as well as engineering. It is at the very core of numerical simulation... In this class, I will explain the concepts of convergence and talk about topology. You will understand the difference between strong convergence and weak convergence... You will learn about different types of spaces including metric spaces, Banach Spaces, Hilbert Spaces and what property can be expected. You will see beautiful lemmas and theorems such as Riesz and Lax-Milgram and I will also describe Lp spaces, Sobolev spaces and provide a few details about PDEs, or Partial Differential Equations."*

Commentary. This is an advanced course for those interested in theory, particularly as the instructors make much of their teaching in the French tradition, favoring the beauty of the math for its own sake over its utility in computation. The Gallic attitude alone may make the course interesting enough to pursue, and in fact it is beautifully produced, including its free online textbook. Aesthetics aside, functional analysis can be useful in the solution of systems of partial differential equations, and other applications of the material arise in areas such as theoretical neuroscience [5] and machine learning methods in bioinformatics [6].

Prerequisites. Calculus. Abstract Algebra would probably be helpful.

Textbook. The course is self-contained, with a very nice online textbook. The Oxford Mathematical Institute also has a set of online notes and course materials for this and many other subjects (http://www.maths.ox.ac.uk/courses/course/22977/material).

Alternatives. Joel Feinstein of the University of Nottingham has a "Functional Analysis" course on iTunes U (https://itunes.apple.com/us/itunes-u/functional-analysis/id396420684).

Topology

N.J. Wildberger, University of New South Wales "Algebraic Topology" (UNSWelearning, 2010: http://www.youtube.com/playlist?list=PL6763F57A61FE6FE8)

> *" A first course in Algebraic Topology, with emphasis on visualization, geometric intuition and simplified computations… [It] introduces us to a wide range of novel objects: the sphere, torus, projective plane, knots, Klein bottle, the circle, polytopes, curves… The course has some novel features, including Conway's ZIP proof of the classification of surfaces, a rational form of turn angles and curvature, an emphasis on the importance of the rational line as the model of the continuum, and a healthy desire to keep things simple and physical."*

Commentary. Having been introduced to point-set topology in any of the Analysis courses above, computational biologists will naturally want to learn more about how topology applies to the turning, twisting, and folding of proteins and nucleic acids. This is a course in algebraic topology, but it covers enough geometric topology to introduce knot theory and other topics that have been seen as relevant to macromolecules [7]. It is not a MOOC but the instructor and videos are both fairly clear.

Prerequisites. Abstract Algebra or familiarity with group theory.

Textbook. None is recommended in the course, but Wikibooks has a particularly thorough treatment of topology (http://en.wikibooks.org/wiki/Topology).

Going Further. Those who like the instructor's distinctive style may want to try some of his other courses in advanced mathematics (https://www.youtube.com/user/njwildberger/playlists), in particular his quixotic advocacy of an idea called rational trigonometry. The "Summer School on Computational Topology and Topological Data Analysis" from 2013 is another topology resource (http://videolectures.net/computationaltopology2013_ljubljana).

Discrete Mathematics

Tom Leighton and Marten van Dijk, Massachusetts Institute of Technology "Mathematics for Computer Science" (MIT OpenCourseWare, Fall 2010: http://ocw.mit.edu/courses/electrical-engineering-and-computer-science/6-042j-mathematics-for-computer-science-fall-2010)

> *"This course covers elementary discrete mathematics for computer science and engineering. It emphasizes mathematical definitions and proofs as well as applicable methods. Topics include formal logic notation, proof methods; induction, well-ordering; sets, relations; elementary graph theory;*

integer congruences; asymptotic notation and growth of functions; permutations and combinations, counting principles; discrete probability."

Commentary. This is a core course for any work on the theoretical end of computational biology. It is not a MOOC but, as is usual with MIT OpenCourseWare, makes available detailed notes, assignments, and exams in addition to the lecture videos. The latter do not have as high a resolution as might be desired, so the book excerpts that are supplied as notes are especially helpful.

Prerequisites. Calculus.

Textbook. The course has its own very complete set of notes (http://ocw.mit.edu/courses/electrical-engineering-and-computer-science/6-042j-mathematics-for-computer-science-fall-2010/readings).

Alternatives. This course displaces the recommendation in the previous catalog for the one taught by Steve Skiena at Stony Brook University (http://www.cs.sunysb.edu/%7Ealgorith/math-video), which remains a good alternative though it is yet more difficult to follow visually. Also see the previous catalog for other alternatives, but note that the Udacity course listed there was withdrawn for quality reasons.

Going Further. Several topics that generally fall under the rubric of discrete math are covered more extensively by other courses in this curriculum, such as Combinatorics, Probability, and Logic.

Combinatorics

Federico Ardila, San Francisco State University and Universidad de los Andes "Enumerative Combinatorics" (San Francisco State University, Fall 2013: http://www.youtube.com/playlist?list=PL-XzhVrXIVeSi7xym1XAfFIxOAaHVhjtP)

"Objects: sets, permutations, partitions, compositions, trees, posets, polytopes, etc. Methods: Bijections, generating functions, Möbius inversion, algebraic and topological methods."

Commentary. This course covers enumerative combinatorics, the most basic flavor of this topic and the one most important to Probability and to computer science in general. The subject is introduced at some length in the Discrete Mathematics course as well. Though not a MOOC, there is a course web site with (handwritten) lecture notes and homework (http://math.sfsu.edu/federico/Clase/EC/ec.html). Those with some background already may prefer the advanced MOOC under Going Further, below.

Alternatives. The Indian Institute of Technology, Bangalore offers a non-MOOC "Combinatorics" taught by L. Sunil Chandran (http://nptel.ac.in/courses/106108051).

Prerequisites. Calculus. Discrete Mathematics would give a head start.

Textbook. *Enumerative Combinatorics* by Richard Stanley [8]. However, the course under Going Further has its own free online textbook (http://algo.inria.fr/flajolet/Publications/book.pdf).

Going Further. For much more on generating functions and their asymptotic analysis, along with the considerable advantages of a MOOC, see the Coursera entry "Analytic Combinatorics" by Michael Sedgewick of Princeton University (https://www.coursera.org/course/ac). In addition to the introductory course above, the Complex Analysis course would be useful background.

Probability ✓

John Tsitsiklis and Patrick Jaillet, Massachusetts Institute of Technology "Introduction to Probability—The Science of Uncertainty" (edX, Winter 2014: https://www.edx.org/course/mitx/mitx-6-041x-introduction-probability-1296)

"Probabilistic modeling and the related field of statistical inference are the keys to analyzing data and making scientifically sound predictions… The course covers all of the basic probability concepts,

including: multiple discrete or continuous random variables, expectations, and conditional distributions... laws of large numbers... the main tools of Bayesian inference methods... an introduction to random processes (Poisson processes and Markov chains)"

Commentary. This course has been taught at MIT for more than half a century, not by the Math Department but in Electrical Engineering and Computer Science. Thus, instead of theorems, it promises an intuitive "but still rigorous and mathematically precise" approach. It differs from the usual first course in probability by devoting time to statistical inference and stochastic processes, which are both useful in computational biology. The homework and exams are suitably challenging (and time-consuming). For a foretaste of the content, one can find the complete set of lecture notes and exams on the MIT OpenCourseWare site (http://ocw.mit.edu/courses/electrical-engineering-and-computer-science/6-041-probabilistic-systems-analysis-and-applied-probability-spring-2006/index.htm).

Evaluation.

Course Level	Hours per Week	Course Grade				Student Grade	Curve
		Lectures	Homework	Assessment	Overall		Pass/"A"
Intermediate	12	95	97	97	96	94	60/—

Prerequisites. Calculus.

Textbook. The instructor's popular textbook [9] is made available for online page-at-a-time viewing. See also the free online book by Charles Grinstead and J. Laurie Snell of Dartmouth College (http://www.dartmouth.edu/%7Echance/teaching%5Faids/books%5Farticles/probability%5Fbook/book.html).

Alternatives. For probability taught by a mathematician (though still without a heavy emphasis on theorems), the video course from Harvard recommended in the previous catalog is still a viable option (http://itunes.apple.com/us/course/statistics-110-probability/id502492375). Though not a MOOC, the iTunes U version comes complete with homework and exams, and Joe Blitzstein is an outstanding blackboard lecturer in the best mathematical tradition. See also the previous catalog for additional alternatives.

Going Further. Again, the previous catalog lists a number of resources for going further in probability, in particular stochastic processes. The latter are also covered in depth in the MIT OpenCourseWare graduate course in "Discrete Stochastic Processes" (http://ocw.mit.edu/courses/electrical-engineering-and-computer-science/6-262-discrete-stochastic-processes-spring-2011).

Statistics 💻

(See listing in Data Sciences Department.)

Logic 💻✓

Michael Genesereth, Stanford University "Introduction to Logic" (Coursera, Fall 2014: https://www.coursera.org/course/intrologic)

"This course is a basic introduction to Logic. It shows how to formalize information in [the] form of logical sentences. It shows how to reason systematically with this information to produce all logical conclusions and only logical conclusions. And it examines logic technology and its applications—in mathematics, science, engineering, business, law, and so forth."

Commentary. Logic has obvious relevance to all of science, and formal logic, on which this course focuses, has particular application to computer science. This includes not only the use of propositional logic in computer hardware and languages, but also the importance of predicate and higher-order logics

to mathematical foundations. This course is a very technical introduction that uses a unique interactive online platform to teach proof systems. It is also somewhat unusual in the form of predicate logic it teaches, which uses the standard syntax but a restricted semantics (Herbrand rather than Tarski semantics, for the cognoscenti) that is better suited to pedagogy and for common use. This relational (as opposed to first-order) logic is also the basis of the Prolog programming language, often associated with artificial intelligence applications, in which a program consists of a database of declarative premises and computation is done as a form of deductive proof.

Evaluation.

Course Level	Hours per Week	Course Grade				Student Grade	Curve
		Lectures	Homework	Assessment	Overall		Pass/"A"
Intermediate	8	94	95	92	93	92.4	63/—

Personal Note. The author spent several years in a logic programming R&D group in industry and still prefers Prolog on occasion, in part because it is well suited to building grammars that are useful for biological sequences and computational linguistics, but also because its underlying relational model allows for rapid prototyping of typical database-driven applications. An extension called constraint logic programming is also good for certain optimization problems. Alas, this beautiful language never really managed to break into the mainstream, but it has its adherents.

Prerequisites. Basic familiarity with set theory.

Textbook. The course is self-contained. However, a book on logic tailored for computer science by the University of Pennsylvania's Jean Gallier (who taught this author) is now available in a reasonably priced Dover edition [10], as well as free online (http://www.cis.upenn.edu/~jean/gbooks/logic.html). An advanced philosophical text can be found at the Open Logic Project (http://openlogicproject.org).

Alternatives. Greg Restall and Jen Davoren of The University of Melbourne teach the course "Logic: Language and Information" Parts 1 and 2, also on Coursera (https://www.coursera.org/course/logic1 and https://www.coursera.org/course/logic2). This is a gentler and more protracted introduction than the course above that teaches the conventional form of first-order logic, as generally used in math and philosophy, but also has an explicit introduction to the Prolog language and covers many applications of logic.

Going Further. For more details on logic programming, see the neat set of course notes by Frank Pfenning of Carnegie Mellon University (http://www.cs.cmu.edu/~fp/courses/lp/lectures/lp-all.pdf). Or, just download a free version like SWI-Prolog (http://www.swi-prolog.org) and start experimenting.

Theory of Computation ✓

Jeff Ullman, Stanford University "Automata" (Coursera, Fall 2013: https://www.coursera.org/course/automata)

> *"This course covers finite automata, context-free grammars, Turing machines, undecidable problems, and intractable problems (NP-completeness)."*

Commentary. This course, carried over from the previous catalog (when it was one of the first Coursera offerings) is a rigorous introduction to the theory of computation, despite the more narrow scope of the instructor's course title. The subject is essential for anyone hoping to flirt with the theoretical end of computational biology, and familiarity with intractability is fundamental for designers of algorithms. The instructor is a legend in the field. Be warned: The course accelerates in difficulty throughout, and the final exam is formidable.

Evaluation. (Note: Student Grade does not take into account optional programming assignments.)

Course Level	Hours per Week	Course Grade				Student Grade	Curve
		Lectures	Homework	Assessment	Overall		Pass/"A"
Intermediate	8	93	95	93	92	85.8	50/—

Prerequisites. Data Structures or an equivalent second course in computer science. Discrete Mathematics is helpful.

Textbook. The course is built around *Introduction to Automata Theory, Languages, and Computation* by John Hopcroft, Rajeev Motwani, and the instructor [11] but its use is optional. A standard text by Michael Sipser of the Massachusetts Institute of Technology, *An Introduction to the Theory of Computation* [12], is awfully pricey, but detailed notes from his 2012 set of classroom lectures are available online (http://web.mit.edu/~holden1/www/coursework/math/18.404/main.pdf). John Savage of Brown University has posted a free version of his well-regarded book entitled *Models of Computation* (http://cs.brown.edu/~jes/book).

Alternatives. For those desiring a somewhat less rigorous introduction, see Sebastian Wernicke's "Intro to Theoretical Computer Science" on Udacity (https://www.udacity.com/course/cs313). It focuses on what is perhaps the most essential topic for bioinformatics, intractability. Moreover, it is taught by a practicing computational biologist and has a strong algorithmic focus. The University of California at Davis has a very comprehensive but non-MOOC course on "Theory of Computation" taught by Dan Gusfield, who has done work in computational biology algorithms (https://itunes.apple.com/us/itunes-u/theory-computation-fall-2011/id585297167); homeworks, exams, and solutions are also online (http://www.cs.ucdavis.edu/~gusfield/cs120f11). See the previous catalog for additional alternatives, especially Harry Lewin's marvelous course at Harvard entitled "Introduction to Formal Systems and Computation" (http://itunes.apple.com/WebObjects/MZStore.woa/wa/viewPodcast?id=429428100).

Linear Programming

Sriram Sankaranarayanan and Shalom Ruben, University of Colorado, Boulder "Linear and Integer Programming" (Coursera, TBA: https://www.coursera.org/course/linearprogramming)

> *"This course will cover the very basic ideas in optimization. Topics include the basic theory and algorithms behind linear and integer linear programming along with some of the important applications. We will also explore the theory of convex polyhedra using linear programming."*

Commentary. Linear programming and its more computationally challenging cousin, integer programming, are basic frameworks for optimization that arise in a number of fields relevant to computational biology. Recently they have found application in such varied areas as gene regulatory networks [13], haplotype assembly [14], flux balance analysis in metabolic networks [15], and protein inference in shotgun proteomics [16]. This course offers multiple tracks, varying in their emphasis on theory versus application, with or without programming assignments.

Prerequisites. Algorithms and/or Theory of Computation.

Textbook. None is required. Thomas Ferguson of the University of California, Los Angeles has a very concise online text (http://www.math.ucla.edu/~tom/LP.pdf), and an early edition of a weightier book by Princeton's Robert Vanderbei is freely available online (http://www.princeton.edu/~rvdb/LPbook).

Alternatives. The non-MOOC "Applied Optimization" from Purdue recommended in the previous catalog remains an alternative (http://www.networkmaths.ie/videos/list_videos.php?course=opt-2), as do other resources listed there.

Going Further. Stanford has a MOOC by Stephen Boyd on "Convex Optimization," a generalization of linear programming (https://class.stanford.edu/courses/Engineering/CVX101/Winter2014/about). The course Discrete Optimization in the Computer Science Department goes much more deeply into integer programming as well as combinatorial optimization.

Numerical Methods

Nathan Kutz, University of Washington "Scientific Computing" (Coursera, TBA: https://www.coursera.org/course/scientificcomp)

> *"This course is a survey of numerical solution techniques for ordinary and partial differential equations. Emphasis will be on the application of numerical schemes to practical problems in the engineering and physical sciences. Apply advanced MATLAB routines and toolboxes to solve problems. Review and practice graphical techniques for information presentation and learn to create visual illustrations of scientific results."*

Commentary. Although this course is titled Scientific Computing by the instructor, it focuses on numerical methods, an important skill set for those who will actually need to solve differential equations and other formulations that have no easy closed form solution. This applies to a lot of real world mathematical and systems biology. This course is recommended for its practical computing component, which uses either MATLAB or Octave, a free (or by donation) alternative. For a somewhat more traditional mathematical approach, see the alternative below.

Prerequisites. Differential Equations, Linear Algebra, Introduction to Computer Science.

Textbook. *Data-Driven Modeling & Scientific Computation*, by the instructor [17]. Notes and/or books provided online by their authors include those by Douglas Arnold of the University of Minnesota (http://www.ima.umn.edu/~arnold/597.00-01) and L. Ridgeway Scott of the University of Chicago (http://people.cs.uchicago.edu/~ridg/newna/s14parisnna.html).

Alternatives. The course recommended in the previous catalog, "Numerical Methods" by Autar Kaw of the University of South Florida, remains an alternative for those less committed to actual programming (https://www.udemy.com/numericalmethodsguy or http://nm.mathforcollege.com), although it still makes use of mathematical software. It has been ported to the Udemy platform, which hosts a number of paid courses, but this one is free. If that framework proves cumbersome, just revert to the alternate link above; both formats involve a mix of videos and texts. See the previous catalog for a more complete commentary.

Going Further. See the Scientific Computing course in the Computer Science Department. Also, for an even more mathematical and extensive treatment of the topic, see the non-MOOC "Numerical Methods of Ordinary and Partial Differental Equations" by G.P. Raja Sekhar of the Indian Institute of Technology, Kharagpur (http://nptel.ac.in/courses/111105038).

Dynamical Systems

Michael Pilant, Texas A&M University "Dynamical Systems and Chaos" (Texas A&M, 2004: http://www.math.tamu.edu/~mpilant/math614)

> *"Discrete maps; continuous flows; dynamical systems; Poincare maps; symbolic dynamics; chaos, strange attractors; fractals; computer simulation of dynamical systems."*

Commentary. This carryover from the previous catalog is not a MOOC and should be considered an elective for mathematically talented students interested in a deep understanding of dynamical systems modeling in biology. It is an individual effort by a math professor, and also provides a wealth of ancillary

web resources. (From the main page, click "Video Lectures" on the left, and then "Archival Videos" at the top.)

Prerequisites. Differential Equations. Complex Analysis and Linear Algebra are highly recommended.

Textbook. *CHAOS: An Introduction to Dynamical Systems* by Kathleen Alligood, Tim Sauer, and James Yorke [18]. Note, however, that Edward Scheinerman of Johns Hopkins University has made freely available his 1996 *Invitation to Dynamical Systems* (http://www.ams.jhu.edu/~ers/invite).

Alternatives. See the previous catalog for alternatives. Of particular note is a non-MOOC but very effectively taught course called "Introduction to Linear Dynamical Systems" by Stephen Boyd of Stanford (http://see.stanford.edu/see/courseinfo.aspx?coll=17005383-19c6-49ed-9497-2ba8bfcfe5f6). Unlike their nonlinear brethren, linear dynamical systems are generally not chaotic and in fact are amenable to analyses (via linear algebra) that can be very useful in engineering, for instance in control systems. It may be of more practical utility than some of the sexier topics in the recommended course above.

Signal Processing

Richard Baraniuk, Rice University "Discrete Time Signals and Systems" (edX, Winter, 2014: https://www.edx.org/course/ricex/ricex-elec301x-discrete-time-signals-1032)

> *"This course will teach students to analyze discrete-time signals and systems in both the time and frequency domains. Students will learn convolution, discrete Fourier transforms, the z-transform, and digital filtering."*

Commentary. As noted in the previous catalog, understanding feedback systems, filters, convolution, and the like is potentially important in areas such as systems biology and neuroinformatics, while Fourier analysis is at the foundation of crystallographic structure determination and biomedical imaging. In fact, signal processing has recently found a number of applications in genomic and proteomic data analysis [19-21]. The course uses MATLAB, which is rather expensive for individuals, but as with several other MOOCs the organizers have arranged for a MATLAB interpreter to be available for free through the edX interface on a temporary basis.

Personal Note. The author's first journal publication in the field of bioinformatics used signal processing techniques for DNA sequence analysis [22]. Even beyond the algorithmic methods afforded by this approach, thinking of the cell itself as a time-varying system in the signal processing sense provides a distinctive framework for modeling and analysis, with well-established theory and tools on which to draw. This subject probably still has a lot more to offer to the field of computational biology.

Prerequisites. Calculus, Complex Analysis, Linear Algebra.

Textbook. The instructor has a set of notes on OpenStax, an online educational resource site he founded (http://cnx.org/content/col10064/latest). The first alternative course below also has an associated free online text, which is especially stylish (http://www.sp4comm.org).

Alternatives. Coursera has "Digital Signal Processing" by Paolo Prandoni and Martin Vetterli of the École Polytechnique Fédérale de Lausanne (https://www.coursera.org/course/dsp), which starts out somewhat faster than the course above. MIT OpenCourseWare has "Signals and Systems" by Dennis Freeman (http://ocw.mit.edu/courses/electrical-engineering-and-computer-science/6-003-signals-and-systems-fall-2011), which however is not a MOOC. Another approach is to take Circuits and Electronics in the Neuroscience Department, which may also be of interest in systems biology and includes some coverage of signal processing. See the previous catalog for additional alternatives.

Going Further. The previous catalog also has recommendations for further work in stochastic signal processing and linear dynamical systems.

Information Theory

Raymond Yeung, The Chinese University of Hong Kong "Information Theory" (Coursera, Winter 2014: https://www.coursera.org/course/informationtheory)

> *"This course is an introduction to information theory, which emphasizes fundamental concepts as well as analytical techniques. Specific topics include: Information Measures, The I-Measure, Zero-Error Data Compression, Weak Typicality, Strong Typicality, Discrete Memoryless Channels, etc."*

Commentary. Information theory arises at various points in computational biology, including machine learning and motif analysis, and ought to be a concern for anyone interested in the transmission of genetic information [23] and the systems view of cell signaling and communication [24]. However, this course is not for the mathematically timid.

Prerequisites. Probability and general mathematical sophistication.

Testbook. The course follows the instructor's popular textbook [25], a preprint of which he has made freely available online (http://iest2.ie.cuhk.edu.hk/~whyeung/book2/main2_draft.pdf). For advanced topics, Mark Wilde of McGill University has posted his book *From Classical to Quantum Shannon Theory* (http://arxiv.org/abs/1106.1445).

Alternatives. Paul Penfield and Seth Lloyd of the Massachusetts Institute of Technology have a course entitled "Information and Entropy" on MIT OpenCourseWare (http://ocw.mit.edu/courses/electrical-engineering-and-computer-science/6-050j-information-and-entropy-spring-2008). See the previous catalog for other alternatives.

Going Further. David Mackay of Cambridge University has a non-MOOC "Course on Information Theory, Pattern Recognition, and Neural Networks" that takes the topic much further down the machine learning path (http://videolectures.net/course_information_theory_pattern_recognition). Another branch in the road would be communication theory, which is comprehensively covered in a pair of graduate courses entitled "Principles of Digital Communications" Parts 1 and 2, taught by Lizhong Zheng, Robert Gallagher, and David Forney at the Massachusetts Institute of Technology on MIT OpenCourseWare (http://ocw.mit.edu/courses/electrical-engineering-and-computer-science/6-450-principles-of-digital-communications-i-fall-2006 and http://ocw.mit.edu/courses/electrical-engineering-and-computer-science/6-451-principles-of-digital-communication-ii-spring-2005). The subject of communications leads naturally to another major theoretical branch, that of cryptography. Stanford's Dan Boneh teaches a two part-course in this topic (https://www.coursera.org/course/crypto and https://www.coursera.org/course/crypto2), as does Johathan Katz of the University of Maryland, College Park (https://www.coursera.org/course/cryptography), which is part of an entire series on "Cybersecurity" (https://www.coursera.org/specialization/cybersecurity/7).

Personal Note. On the subject of cryptography, those with the opportunity should visit Bletchley Park, the British code-breaking center where computing pioneer Alan Turing worked during the Second World War (http://www.bletchleypark.org.uk). Visitors can see the office in "Hut 8" where he worked, as it was then, as well as functioning versions of the ur-computers that he helped to develop. Bletchley is an intensely atmospheric place, heartily recommended for those interested in the history of computing (and it should be remembered that Turing went on to work on applications of computing to biology after the war, up to the time of his tragic death).

Game Theory ✓

Matthew Jackson, Kevin Leyton-Brown, and Yoav Shoham, Stanford University and the University of British Columbia "Game Theory" (Coursera, Fall 2013: https://www.coursera.org/course/gametheory)

“Popularized by movies such as ‘A Beautiful Mind,’ game theory is the mathematical modeling of strategic interaction among rational (and irrational) agents. Beyond what we call 'games' in common language, such as chess, poker, soccer, etc., it includes the modeling of conflict among nations, political campaigns, competition among firms, and trading behavior in markets... The course will provide the basics: representing games and strategies, the extensive form (which computer scientists call game trees), Bayesian games (modeling things like auctions), repeated and stochastic games, and more.”

Commentary. As noted in the previous catalog, game theory has long been used in the study of evolutionary dynamics, and also bears on modeling and network theory. Scientists in any quantitative field probably ought to be familiar with such basic ideas as the prisoners’ dilemma, Pareto optimality, and Nash equilibria, or indeed with any field that has produced eight Nobel prizes.

Evaluation.

Course Level	*Hours per Week*	*Course Grade*				*Student Grade*	*Curve*
		Lectures	*Homework*	*Assessment*	*Overall*		*Pass/”A”*
Intermediate	8	88	93	92	90	93.9	70/90

Prerequisites. Some Calculus and Probability. General mathematical sophistication.

Textbook. The instructors have a textbook called *Essentials of Game Theory* [26] that is under $10 as an e-book. Thomas Ferguson of the University of California, Los Angeles also has a free online text with a traditional mathematical approach (http://www.math.ucla.edu/~tom/Game_Theory/Contents.html).

Alternatives. See the previous catalog for several alternatives.

Going Further. The follow-on to this course by the same instructors extends to advanced applications in social choice, mechanism design, and auctions (https://www.coursera.org/course/gametheory2). It includes a proof of Arrow’s Theorem, which famously demonstrated why there can be no perfect voting mechanism. The latter topic also figures in Coursera’s “Making Better Group Decisions: Voting, Judgement Aggregation and Fair Division” by Eric Pacuit of the University of Maryland, College Park (https://www.coursera.org/course/votingfairdiv). Also on Coursera, Michael Genesereth of Stanford teaches “General Game Playing” (https://www.coursera.org/course/ggp), a completely different take on strategy games involving algorithm discovery in artificial intelligence style, and Tom Morley of the Georgia Institute of Technology teaches “Games without Chance: Combinatorial Game Theory” (https://www.coursera.org/course/cgt), for yet another mathematical structure dealing with strategy.

Graph Theory

L. Sunil Chandran, Indian Institute of Science, Bangalore “Graph Theory” (NPTEL: http://nptel.ac.in/courses/106108054)

“In computer science, graph theory is used extensively. The intension of this course is to introduce the subject of graph theory to computer science students in a thorough way... While the course will cover all elementary concepts such as coloring, covering, hamiltonicity, planarity, connectivity and so on, it will also introduce the students to some advanced concepts.”

Commentary. Graph theory has always been important in the study of algorithms and in network analysis, and more recently it has been applied in arenas such as omic interactions and brain connectivity maps. While much of what is needed in this regard can be obtained from network-oriented courses in the Systems Biology Department, the material in this advanced course would afford more in-depth mathematical analyses. It is not a MOOC, and may be heavy going without notes since the instructor’s handwriting is a bit casual. This course, like others in this catalog from the Indian Institutes of Technology (IIT) and of Science (IISc), is part of the extensive offerings of the Indian National

Programme on Technology Enhanced Learning (NPTEL) described more fully in the previous catalog (http://nptel.ac.in).

Textbook. The instructor recommends *Graph Theory* by Reinhard Diestel [27], among others. It is available as a free preview from the author (http://diestel-graph-theory.com).

Computer Science Department

Computer science is an obvious fit to the MOOC paradigm, and there has been a profusion of courses. In the following curriculum, the first three courses together should approximate a typical lower-level core sequence for computer science programs. Another approach now being taken, though, is to package a series of courses from the same institution into such a core sequence. Coursera, for example, has what they call a Specialization in "Fundamentals of Computing" from Rice University, which puts together a coordinated series of courses comprising "An Introduction to Interactive Programming in Python," "Principles of Computing," and "Algorithmic Thinking," to be followed by a comprehensive "Capstone Exam" (https://www.coursera.org/specialization/fundamentalscomputing/9). At the same time, edX has similarly packaged what they call an XSeries in "Foundations of Computer Science" from the Massachusetts Institute of Technology (https://www.edx.org/xseries). This includes the first course below plus its extension "Introduction to Computational Thinking and Data Science" followed by "Software Construction in Java" parts 1 and 2, and finally a series of "Computation Structures" courses covering "Digital Circuits," "Programmable Architectures," and "Computer Systems Organization." These courses require fees for both individual and cumulative certificates, but should be available for free without certification if edX stays true to form. While it is too soon to favor these particular products over the established courses below, the trend toward coordinated MOOCs from single institutions is a welcome one and may become the preferred option very soon.

Introduction to Computer Science 💻

Eric Grimson, John Guttag, and Ana Bell, Massachusetts Institute of Technology "Introduction to Computer Science and Programming Using Python" (edX, Summer 2014: https://www.edx.org/course/mitx/mitx-6-00-1x-introduction-computer-1841)

> *"This subject is aimed at students with little or no programming experience. It aims to provide students with an understanding of the role computation can play in solving problems. It also aims to help students, regardless of their major, to feel justifiably confident of their ability to write small programs that allow them to accomplish useful goals. The class will use the Python programming language."*

Commentary. This recommendation is carried over from the previous catalog, where further commentary can be found. A major reason this course is recommended is that it teaches Python, commonly used in bioinformatics.

Textbook. *Introduction to Computation and Programming Using Python* by the instructor John Guttag [28]. Other possibilities include the free online textbook *Python for Informatics* by Charles Severance of the University of Michigan (http://www.pythonlearn.com/book.php), who also teaches one of the alternative courses listed below.

Personal Note. The author's initial exposure to computers was in his first semester at this institution (though in a different course) and involved late nights sitting at a keypunch machine generating thick stacks of IBM punch cards, loading the job decks in a large feed hopper, waiting for output at a clattering line printer, and then repeating the process until the program compiled and ran. MOOCs are better.

Alternatives. On Coursera, introductory courses using Python are also taught by Stanford's Nick Parlante (https://www.coursera.org/course/cs101), Jennifer Campbell and Paul Gries of the University of Toronto (https://www.coursera.org/course/programming1), Joe Warren, Scott Rixner, John Greiner, and Stephen Wong of Rice University (https://www.coursera.org/course/interactivepython), and Charles Severance of the University of Michigan (https://www.coursera.org/course/pythonlearn). For

other languages, edX offers alternatives including an eclectic self-paced course by Harvard's David Malan (https://www.edx.org/course/harvardx/harvardx-cs50x-introduction-computer-1022) that teaches C, PHP, Javascript, and SQL; an "Introduction to Computing with Java" by Ting-Chuen Pong of the Hong Kong University of Science and Technology (https://www.edx.org/course/hkustx/hkustx-comp102x-introduction-computing-1690); and a two-part "Introduction to Computer Science" by Deepak Phatak of the Indian Institute of Technology, Bombay that makes use of C and C++ (https://www.edx.org/course/iitbombayx/iitbombayx-cs101-1x-introduction-1447 and https://www.edx.org/course/iitbombayx/iitbombayx-cs101-2x-introduction-1448). See the previous catalog for a discussion of programming languages for bioinformatics.

Going Further. The continuation of the recommended MIT course, "Introduction to Computational Thinking and Data Science" (https://www.edx.org/course/mitx/mitx-6-00-2x-introduction-computational-1505), by the same instructors and using the same textbook, covers a miscellany of topics including plotting, stochastic programs, probability and statistics, random walks, Monte Carlo simulations, modeling data, optimization problems, and clustering. This sampling might be a suitable termination for those going no further in computer science proper, mainly for the additional practice in coding, but otherwise the courses immediately below or the remainder of the MIT XSeries described above would be the appropriate continuation.

Data Structures

Kevin Wayne and Robert Sedgewick, Princeton University "Algorithms: Part I" and "Algorithms: Part II" (Coursera, Winter/Spring 2014: https://www.coursera.org/course/algs4partI and https://www.coursera.org/course/algs4partII)

> [Part 1] *"[E]lementary data structures, sorting, and searching. Topics include union-find, binary search, stacks, queues, bags, insertion sort, selection sort, shellsort, quicksort, 3-way quicksort, mergesort, heapsort, binary heaps, binary search trees, red-black trees, separate chaining and linear probing hash tables, Graham scan, and kd-trees."* [Part 2] *"[G]raph and string-processing algorithms. Topics include depth-first search, breadth-first search, topological sort, Kosaraju-Sharir, Kruskal, Prim, Dijkistra, Bellman-Ford, Ford-Fulkerson, LSD radix sort, MSD radix sort, 3-way radix quicksort, multiway tries, ternary search tries, Knuth-Morris-Pratt, Boyer-Moore, Rabin-Karp, regular expression matching, run-length coding, Huffman coding, LZW compression, and the Burrows-Wheeler transform."*

Commentary. Although labeled Algorithms, these courses cover data structures in some depth and together are typical of a particularly thorough second course in computer science; in fact, over a quarter of all Princeton undergraduates take the classroom version. Java is required, though not a deep knowledge of it, making this an opportunity for students of bioinformatics to pick up another relevant language with a little extra self-study. One major caveat is that Princeton, for reasons all its own, does not issue certificates or any other acknowledgement of participation, which may be a showstopper for some online learners with specific career goals.

Prerequisites. Introduction to Computer Science and basic familiarity with Java, which can be acquired by preliminary self-study or with the help of an online tutorial such as John Purcell's "Java for Complete Beginners" on Udemy (https://www.udemy.com/java-tutorial) or Cay Horstmann's "Intro to Programming: Problem Solving with Java" on Udacity (https://www.udacity.com/course/cs046).

Textbook. The course is self-contained, but the instructors have an optional textbook [29] with an elaborate web site (http://algs4.cs.princeton.edu/home). Pat Morin of Carleton University has an open content textbook (http://opendatastructures.org).

Alternatives. This material roughly corresponds to the Berkeley course in Data Structures by Jonathan Shewchuk that was listed in the previous catalog (and whose course title we retain for this entry)

(http://webcast.berkeley.edu/playlist#c,d,Computer_Science,-XXv-cvA_iAlnI-BQr9hjqADPBtujFJd or http://www.youtube.com/view_play_list?p=-XXv-cvA_iAlnI-BQr9hjqADPBtujFJd). The Berkeley course explicitly teaches Java from scratch and is a somewhat more general second course in computer science, but it is not a MOOC. See also other non-MOOC alternatives listed there.

Going Further. MIT OpenCourseWare has the non-MOOC "Advanced Data Structures" by Erik Demaine (http://ocw.mit.edu/courses/electrical-engineering-and-computer-science/6-851-advanced-data-structures-spring-2012). As a point of interest, Demaine is also known for his work in computational origami, described in a talk at the U.S. National Institutes of Health entitled "Geometric Folding Algorithms: Linkages, Origami, Polyhedra" (http://videocast.nih.gov/Summary.asp?File=17645). Note the obvious connection to protein folding and biological self-assembly.

Machine Structures

Gaetano Borriello and Luis Ceze, University of Washington "The Hardware/Software Interface" (Coursera, TBA: https://www.coursera.org/course/hwswinterface)

> *"This course examines key computational abstraction levels below modern high-level languages; number representation, assembly language, introduction to C, memory management, the operating-system process model, high-level machine architecture including the memory hierarchy, and how high-level languages are implemented. We will develop students' sense of "what really happens" when software runs—and that this question can be answered at several levels of abstraction, including the hardware architecture level, the assembly level, the C programming level and the Java programming level."*

Commentary. This is an important course for those who need to get the most out of their hardware. The exposure to C and assembly language will itself be useful to bioinformatics developers who might need to tune the performance of compute-intensive applications, but even more important are the general insights this course will provide in how to make the best use of memory, cycles, and bandwidth.

Prerequisites: Data Structures. Exposure to C or Java.

Textbook. Recommended but not required is the costly *Computer Systems: A Programmer's Perspective* by Randal Bryant and David O'Hallaron [30]. Although not tied to the course, an alternative might be *Programming from the Ground Up* by Jonathan Bartlett [31], which is available online under a GNU Free Documentation License (http://programminggroundup.blogspot.com).

Alternatives. The name of this listing is again borrowed from the previous catalog's non-MOOC recommendation, still an option, which was stylishly taught by Dan Garcia at the University of California, Berkeley (http://webcast.berkeley.edu/playlist#c,d,Computer_Science,-XXv-cvA_iDHtKXLFJbDG-i6L9oDr5X9 or http://www.youtube.com/view_play_list?p=-XXv-cvA_iDHtKXLFJbDG-i6L9oDr5X9). See the previous catalog for other alternatives as well.

Going Further. Standard computer science curricula have upper-level courses in compilers and in computer architecture that would be natural follow-ons for those interested. Coursera has "Compilers" by Stanford's Alex Aiken (https://www.coursera.org/course/compilers) and "Computer Architecture" by Princeton's David Wentzlaff (https://www.coursera.org/course/comparch). Consider also the graduate course in "Performance Engineering of Software Systems" taught by Saman Amarasinghe and Charles Leiserson of the Massachusetts Institute of Technology on MIT OpenCourseWare (http://ocw.mit.edu/courses/electrical-engineering-and-computer-science/6-172-performance-engineering-of-software-systems-fall-2010) for more insights on code optimization and squeezing performance out of hardware.

Algorithms 💻✓

Tim Roughgarden, Stanford University "Algorithms: Design and Analysis, Part 1" and "Algorithms: Design and Analysis, Part 2" (Coursera, Spring/Summer 2014: https://www.coursera.org/course/algo and https://www.coursera.org/course/algo2) (Stanford Online, Spring/Summer 2014: http://online.stanford.edu/courses/algorithms-spring-2014 and http://online.stanford.edu/course/algorithms-design-and-analysis-part-2-su-2014)

> [Part 1] *"Asymptotic analysis including big-oh notation. Divide-and-conquer algorithms for sorting, counting inversions, matrix multiplication, and closest pair… Running time analysis of divide-and-conquer algorithms. The master method. Introduction to randomized algorithms, with a probability review. QuickSort… More on randomized algorithms and probability. Computing the median in linear time. A randomized algorithm for the minimum graph cut problem… Graph primitives. Depth- and breadth-first search. Connected components in undirected graphs. Topological sort in directed acyclic graphs. Strongly connected components in directed graphs… Dijkstra's shortest-path algorithm. Introduction to data structures. Heaps and applications… Further data structures. Hash tables and applications. Balanced binary search trees."* [Part 2] *"The greedy algorithm design paradigm. Applications to optimal caching and scheduling. Minimum spanning trees and applications to clustering. The union-find data structure. Optimal data compression… The dynamic programming design paradigm. Applications to the knapsack problem, sequence alignment, shortest-path routing, and optimal search trees… Intractable problems and what to do about them. NP-completeness and the P vs. NP question. Solvable special cases. Heuristics with provable performance guarantees. Local search. Exponential-time algorithms that beat brute-force search."*

Commentary. Compared to the Data Structures courses above, which also cover algorithms, these courses are considered upper-level and stress analysis over implementation, even though they include hefty programming assignments. The instructor has an unintimidating, indeed rather breezy style, and the syllabus is not as rigidly organized as many other treatments of the topic area, but in the end it is effective. The material is largely presented in handwritten form, as on a whiteboard, and thankfully it is legible if not elegant. The programming assignments can be done in the language of your choice since they are graded by whether you correctly return some numerical answer to a problem so compute-intensive as to mandate the "correct" implementation (unless you happen to have access to a supercomputer and are able to brute-force it).

Evaluation. (Part 1 only)

Course Level	*Hours per Week*	*Course Grade*				*Student Grade*	*Curve*
		Lectures	*Homework*	*Assessment*	*Overall*		*Pass/"A"*
Introductory	8	91	95	94	93	96.8	70/—

Prerequisites. Data Structures.

Textbook. None is required. A standard text is *Introduction to Algorithms* by Thomas Cormen, Charles Leiserson, Ronald Rivest, and Clifford Stein of the Massachusetts Institute of Technology [32]. It is expensive, but a nice set of lecture notes were extracted from it in 2003 by David Mount of the University of Maryland (http://www.cs.umd.edu/~mount/451/Lects/451lects.pdf).

Alternatives. For theory-oriented students, a much more mathematical approach without programming assignments can be found in "Analysis of Algorithms" by Robert Sedgewick of Princeton University (https://www.coursera.org/course/aofa). The non-MOOC recommended in the previous catalog, "Introduction to Algorithms" by Charles Leiserson and Erik Demaine on MIT OpenCourseWare (http://ocw.mit.edu/courses/electrical-engineering-and-computer-science/6-046j-introduction-to-algorithms-sma-5503-fall-2005) is more traditionally organized and is a prototype for such courses. A

version of this course at videolectures.net has the videos conveniently synched with slides in a side-by-side display (http://videolectures.net/mit6046jf05_introduction_algorithms). Berkeley Webcast has a non-MOOC course entitled "Efficient Algorithms and Intractable Problems" taught by Satesh Rao (http://webcast.berkeley.edu/playlist#c,s,Fall_2013,-XXv-cvA_iBapEvcNwRuTJVt6BPpcMaJ or http://www.youtube.com/view_play_list?p=-XXv-cvA_iBapEvcNwRuTJVt6BPpcMaJ). Other alternatives are detailed in the previous catalog.

Going Further. See the corresponding section under Introduction to Algorithms in the previous catalog for graduate level (non-MOOC) courses.

Operating Systems

Anthony Joseph and John Canny, University of California, Berkeley "Operating Systems and Systems Programming" (Berkeley Webcast, Fall 2013: http://webcast.berkeley.edu/playlist#c,d,Computer_Science,-XXv-cvA_iDrt_oPWfQ4-fjHm2KSSOPq) or https://www.youtube.com/view_play_list?p=-XXv-cvA_iDrt_oPWfQ4-fjHm2KSSOPq)

> *"Basic concepts of operating systems and system programming. Utility programs, subsystems, multiple-program systems. Processes, interprocess communication, and synchronization. Memory allocation, segmentation, paging. Loading and linking, libraries. Resource allocation, scheduling, performance evaluation. File systems, storage devices, I/O systems. Protection, security, and privacy."*

Commentary. Knowledge of operating systems and systems programming can be useful if not essential for bioinformatics practitioners at the IT end of the spectrum, for instance those who are the local lab sysadmins or who manage core facilities. This upper-level Berkeley course is well taught and fairly complete (unlike some Berkeley Webcasts). However the course is oriented toward group projects, which of course are irrelevant to an outside audience.

Prerequisites. Machine Structures. Discrete Mathematics helpful.

Textbook. *Operating Systems Concepts* by Abraham Silberschatz, Peter Galvin, and Greg Gagne [33]. A detailed set of notes based on the book was done by John Bell of the University of Illinois, Chicago (http://www.cs.uic.edu/~jbell/CourseNotes/OperatingSystems/index.html).

Alternatives. "Introduction to Linux" by Jerry Cooperstein of the Linux Foundation on edX (https://www.edx.org/course/linuxfoundationx/linuxfoundationx-lfs101x-introduction-1621) may not be as general as the course above, but it has the advantages of a MOOC and Linux is so pervasive that this course may be preferred by many.

Software Engineering

Balaji Srinivasan and Vijay Pande, Stanford University "Startup Engineering" (Coursera, Summer 2013: https://www.coursera.org/course/startup)

> *"Bridges the gap between academic computer science and production software engineering. Fast-paced introduction to key tools and techniques (command line, dotfiles, text editor, distributed version control, debugging, testing, documentation, reading code, deployments), featuring guest appearances by senior engineers from successful startups and large-scale academic projects. Over the course of the class, students will build a command line application, expose it as a web service, and then link other students' applications and services together to build an HTML5 mobile app. General principles are illustrated through modern Javascript and the latest web technologies, including Node, Backbone, Coffeescript, Bootstrap, Git, and Github."*

Commentary. This intensive 12-week course covers the bases of software engineering as it is purportedly practiced in Silicon Valley, emphasizing command line programming with a large suite of web technologies and standalone tools *du jour*. It is unique in alternating lessons in hard-core computing with discussions of the business aspects of startups, and involves the actual web deployment of a student "product." The course feels like drinking from a fire hose but total immersion should put you well on your way to being capable of deploying major bioinformatics applications.

Prerequisites. Introduction to Computer Science. Significant programming experience. Exposure to HTML, CSS, and Javascript helpful.

Textbook. For the entrepreneurial side of the course, the course notes by Stanford's Peter Thiel are recommended (http://blakemasters.tumblr.com/peter-thiels-cs183-startup). A good nuts-and-bolts software engineering book by Ivan Marsic of Rutgers, updated as of 2012, has been made freely available by him online (http://www.ece.rutgers.edu/~marsic/books/SE).

Alternatives. Another approach to software engineering is evident in the course recommended in the previous catalog, which is now available on edX. It is "Engineering Software as a Service" parts 1 and 2 by Berkeley's Armando Fox and David Patterson (https://www.edx.org/course/uc-berkeleyx/uc-berkeleyx-cs169-1x-engineering-1377 and https://www.edx.org/course/uc-berkeleyx/uc-berkeleyx-cs169-2x-engineering-1379), which teaches agile programming methodologies using Ruby on Rails. This course seems to pay more attention to design patterns and frameworks, project management, and other high-level concerns, whereas the Stanford course above may appeal more to down-in-the-weeds coders. "Pattern-Oriented Software Architectures for Concurrent and Networked Software" by Douglas Schmidt of Vanderbilt University goes further with patterns and frameworks in the context of object-orientation and middleware (https://www.coursera.org/course/posasoftware). See also the previous catalog.

Going Further. Udacity offers specialized software engineering courses that include "Software Testing" by John Regehr of the University of Utah (https://www.udacity.com/course/cs258) and "Software Debugging" by Andreas Zeller of Saarland University (https://www.udacity.com/course/cs259).

Human-Computer Interaction 💻

Scott Klemmer, University of California, San Diego "Human-Computer Interaction" (Coursera, Fall 2013: https://www.coursera.org/course/hciucsd)

> *"You'll learn several techniques for rapidly prototyping and evaluating multiple interface alternatives—and why rapid prototyping and comparative evaluation are essential to excellent interaction design. You'll learn how to conduct fieldwork with people to help you get design ideas. How to make paper prototypes and low-fidelity mock-ups that are interactive—and how to use these designs to get feedback from other stakeholders like your teammates, clients, and users. You'll learn principles of visual design so that you can effectively organize and present information with your interfaces. You'll learn principles of perception and cognition that inform effective interaction design. And you'll learn how to perform and analyze controlled experiments online."*

Commentary. Bioinformatics applications and even major online resources are often sadly lacking in attention to good ergonomics. Do your users a favor and absorb these principles of interface design.

Prerequisites. Programming experience.

Personal Note. The author has served on a number of external Advisory Boards for organizations that deploy major bioinformatics web resources, and each of those organizations has greatly improved its product by employing a human-computer interaction specialist. Programmers as a breed tend to feel that they are natural-born interface designers, but it is the exception rather than the rule.

Web Development

Steve Huffman "Web Development" (Udacity: https://www.udacity.com/course/cs253)

> *"Introduction to HTTP and Web Applications… Getting and processing user input… Databases and permanent state… User authentication and access control… Web applications as services, using APIs… Scaling, caching, optimizations… Building a successful web application…"*

Commentary. This is an appropriate course for those who want to field their web applications without having to become full-fledged software engineers. Note that the commentary above for the course Human-Computer Interaction still applies. Note that Udacity charges for access to the full set of course materials and for a certificate, but as of the moment the lectures and notes are available for free.

Textbook. The course has web-based notes. Also, the Free Technology Academy has an online book, *Introduction to Web Applications Development* by Carles Mateu (http://ftacademy.org/materials/fsm/4).

Alternatives. There are more substantive courses in this space that may be preferred by established programmers, including Coursera's "Web Application Architectures" by Greg Heilman of the University of New Mexico, which teaches the Ruby on Rails framework and covers a fair bit of software engineering practice (https://www.coursera.org/course/webapplications). The course from Harvard recommended in the previous catalog, "Building Dynamic Websites" by David Malan (http://cs75.tv), was updated in 2012, though it is not a MOOC. See the previous catalog for other alternatives.

Going Further. The Software Engineering course in this Department is also oriented to web applications, but is far and away more extensive.

Mobile Computing

Frank Bentley and Ed Barrett, Massachusetts Institute of Technology "Building Mobile Experiences" (edX, Winter 2014: https://www.edx.org/course/mitx/mitx-21w-789x-building-mobile-1310)

> *"Mobile devices are changing the ways that we interact with each other and information in the world. This course will take you from a domain of interest, through generative research, design, usability, implementation and field evaluation of a novel mobile experience. You'll finish the course with a working, field-tested application suitable for release in the app store as well as a deep understanding of human interaction with mobile devices and services."*

Commentary. Mobile computing has recently seen an influx of applications in the arenas of structural bioinformatics [34], health informatics [35], and cheminformatics [36]. Because building a mobile app requires choosing sides between Android and Apple, an advanced course is listed here that is agnostic between the two, but requires previous experience in either Java for Android or Objective C for iOS. More elementary courses providing the necessary background are listed under Alternatives below.

Prerequisites. Java for Android or Objective C for iOS.

Textbook. *Building Mobile Experiences*, by the instructors [37]. The book is reasonably priced, but in any case all the necessary readings from it are made available online through the course.

Alternatives. Coursera offers a Specialization from the University of Maryland, College Park and Vanderbilt University on the general subject of "Mobile Cloud Computing with Android" (https://www.coursera.org/specialization/mobilecloudcomputing/2) that includes three substantial courses and a project. Taking the entire series would obviate the course recommended above, or taking just the initial course in the series, "Programming Mobile Applications for Android Handheld Systems" could serve as more than adequate introduction. On the iOS side, there are several non-MOOC alternatives including one taught by Paul Hegarty of Stanford on iTunes U, "Developing iOS 7 Apps for iPhone and iPad" (https://itunes.apple.com/us/course/developing-ios-7-apps-for/id733644550).

Embedded Systems

Jonathan Valvano and Ramesh Yerraballi, University of Texas at Austin "Embedded Systems—Shape the World" (edX, Winter 2014: https://www.edx.org/course/utaustinx/utaustinx-ut-6-01x-embedded-systems-1172)

> *"This is a hands-on, learn-by-doing course that shows you how to build solutions to real-world problems using embedded systems. Each student will purchase a Texas Instruments TM4C123 microcontroller kit and a few electronic components... The course uses a bottom-up approach to problem-solving building gradually from simple interfacing of switches and LEDs to complex concepts like display drivers, digital to analog conversion, generation of sound, analog to digital conversion, graphics, interrupts, and communication. We will present both general principles and practical tips for building circuits and programming the microcontroller in the C programming language. You will develop debugging skills using oscilloscopes, logic analyzers, and software instrumentation."*

Commentary. Those interested in laboratory instrumentation and automation should consider this course, which may sound intimidating but is a required freshman course at UT Austin for electrical engineers and some biomedical engineers. While "embedded systems" here refers to CPUs in equipment other than computers, the use of electronics embedded *in vivo*, and especially in the brain, is an increasingly important therapeutic arena with novel computational opportunities.

Prerequisites. Programming experience.

Textbook. *Embedded Systems: Introduction to Arm® Cortex(TM)-M Microcontrollers*, self-published by the first instructor, is optional [38].

Alternatives. A broader view of this general theme is offered in Berkeley's "Cyber-Physical Systems" by Edward Lee, Sanjit Seshia, and Jeff Jensen (https://www.edx.org/course/uc-berkeleyx/uc-berkeleyx-eecs149-1x-cyber-physical-1629), which encompasses medical devices and systems.

Going Further. The next step after programming microcontrollers is actually developing chips, which is by no means an unrealistic goal today. The course "VLSI CAD: Logic to Layout" by Rob Rutenbar of the University of Illinois at Urbana-Champaign covers tools for creating Application Specific Integrated Circuit (ASIC) and System on Chip (SoC) designs (https://www.coursera.org/course/vlsicad). ASICs, Graphics Processing Units (GPUs), and Field Programmable Gate Arrays (FPGAs) have been used to supercharge a wide variety of bioinformatics applications, such as short-read mapping [39], multiple sequence alignment [40], and phylogenetic reconstruction [41].

Scientific Computing

Randall LeVeque, University of Washington "High Performance Scientific Computing" (Coursera, Spring 2014: https://www.coursera.org/course/scicomp)

> *"Programming-oriented course on effectively using modern computers to solve scientific computing problems arising in the physical/engineering sciences and other fields. Provides an introduction to efficient serial and parallel computing using Fortran 90, OpenMP, MPI, and Python, and software development tools such as version control, Makefiles, and debugging."*

Commentary. Those who intend to use computers exclusively for their own science rather than for deploying applications, and whose science is of the number-crunching variety, can take this course instead of the Machine Structures and Software Engineering courses above and get right down to business. Note, however, that the term "scientific computing" is often taken to refer to a particular set of methods, including simulation and numerical analysis, that are typically applied to scientific problems at scale. For a greater emphasis on this sense of scientific computing, consider the alternative below, or

take the Numerical Methods course in the Mathematics Department in combination with this one from the same institution.

Personal Note. This course and the alternative below may be the only opportunity for students to be exposed to the venerable Fortran language, which the author learned in his first computer course (in fact, the very first course in the MIT catalog then and now, Civil Engineering 1.00), when the GOTO statement was still in full flower. Today's Fortran is not only structured but also object-oriented and concurrent. It maintains a firm foothold in certain scientific fields (including computational chemistry), particularly for supercomputing and linear algebra applications, so it is of more than historical interest.

Prerequisites. Programming and debugging experience. Calculus. Linear Algebra. Numerical Methods would be a plus.

Textbook. None is required.

Alternatives. Although not a MOOC, Berkeley's Philip Colella begins with similar material in "Software Engineering for Scientific Computing" (http://webcast.berkeley.edu/playlist#c,s,Fall_2013,-XXv-cvA_iB8Arh4Szxk275Cu4uRxVt4 or http://www.youtube.com/view_play_list?p=-XXv-cvA_iB8Arh4Szxk275Cu4uRxVt4). His course includes more about methodologies for calculation and simulation in what he calls the "dominant algorithmic motifs found in scientific computing: dense and sparse linear algebra, structured and unstructured grid methods, N-body problems, FFT [Fast Fourier Transform]." The course requires some experience in C/C++. For a preview and insight into the instructor's philosophy see http://www.youtube.com/watch?v=ngmqL48RG3c. For another skills-oriented course, the UC Irvine Open Chemistry initiative (see Chemistry Department) has "Scientific Computing Skills" (http://ocw.uci.edu/openchem/chem5.html) that requires chemistry background and uses Mathematica.

Prerequisites. Experience in C++.

Parallel Computing

Wen-mei Hwu, University of Illinois at Urbana-Champaign "Heterogeneous Parallel Programming" (Coursera, Winter 2014: https://www.coursera.org/course/hetero)

> *"This course introduces concepts, languages, techniques, and patterns for programming heterogeneous, massively parallel processors… It covers heterogeneous computing architectures, data-parallel programming models, techniques for memory bandwidth management, and parallel algorithm patterns."*

Commentary. This course focuses on GPU programming in CUDA and OpenCL, for those who may be interested in implementing fast parallel algorithms (possibly even on their own desktop computers). Not covered is MapReduce/Hadoop programming in the cloud, for which students should consult the Data Science Department listings.

Prerequisites. Programming experience in C/C++.

Textbook. *Programming Massively Parallel Processors,* by David Kirk and the instructor, is optional [42]. As an alternative, Norm Matloff of the University of California, Davis has an open textbook *Programming on Parallel Machines* (http://heather.cs.ucdavis.edu/parprocbook).

Alternatives. Udacity has an "Intro to Parallel Programming" MOOC along similar lines (https://www.udacity.com/course/cs344) taught by David Luebke (who helped found NVIDIA Research) and John Owens of the University of California, Davis. See the previous catalog for other alternatives.

Programming Languages 💻✓

Dan Grossman, University of Washington "Programming Languages" (Coursera, Winter 2013: https://www.coursera.org/course/proglang)

> *"Investigate the basic concepts behind programming languages, with a strong emphasis on the techniques and benefits of functional programming. Use the programming languages ML, Racket, and Ruby in ways that will teach you how the pieces of a language fit together to create more than the sum of the parts. Gain new software skills and the concepts needed to learn new languages on your own."*

Commentary. This course is a gem. The instructor is lucid, engaging, and runs epic terminal sessions practically without a slip. The programming exercises are tough but fair, while serving important pedagogic goals. Learning ML, Racket, and Ruby would alone be worth the price of admission, but there are many important general principles conveyed in the process. Despite the avowed emphasis on functional programming, object-oriented concepts are also well covered.

Evaluation.

Course Level	*Hours per Week*	*Course Grade*				*Student Grade*	*Curve*
		Lectures	*Homework*	*Assessment*	*Overall*		*Pass/"A"*
Intermediate	10	97	98	96	98	94.7	70/—

Personal Note. The Racket language taught in this course is the latest incarnation, via Scheme, of the legendary LISP programming language, the second-oldest high-level programming language (after Fortran). LISP was the mainstay of the AI community from its inception (see Artificial Intelligence, below), and the author not only did serious coding with it but for a while even helped to market LISP machines that implemented the language in hardware. As the archetype of functional programming, LISP has always been at the foundation of programming language theory, strongly influencing such "researchy" languages as Haskell and Clojure, but it also left its residue in hacker culture, for instance in the Emacs editor. You know you've been around the block if you can define CAR and CDR, but you are truly old school if you also remember what those acronyms stand for, and why.

Prerequisites. Introduction to Computer Science. Data Structures.

Textbook. None is required. Scott Smith at Johns Hopkins University has posted a free online textbook (http://www.cs.jhu.edu/~scott/pl/book/dist), and the last-mentioned course under Alternatives below also has an associated online textbook.

Alternatives. "Paradigms of Computer Programming" by Peter Van Roy of the Université Catholique de Louvain (https://www.edx.org/course/louvainx/louvainx-louv1-01x-paradigms-computer-1203) is another very good course in this vein on edX. It has just a tad more of a theoretical emphasis, for instance stressing invariant programming as a basis for iteration and recursion, but since it uses a pedagogical generic programming system (Mozart) it lacks the side benefit of teaching several practical as well as "theoretical" languages like the recommended course above. Shriram Krishnamurthi of Brown University has several editions of his "Programming Languages" course online, including assignments (http://cs.brown.edu/courses/cs173/2012), but it is not a MOOC and so should be a last resort.

Functional Programming 💻✓

Martin Odersky, École Polytechnique Fédérale de Lausanne "Functional Programming Principles in Scala" (Coursera, Fall 2013: https://www.coursera.org/course/progfun)

> *"This course introduces the cornerstones of functional programming using the Scala programming language. Functional programming has become more and more popular in recent years because it promotes code that's safe, concise, and elegant. Furthermore, functional programming makes it easier*

to write parallel code for today's and tomorrow's multiprocessors by replacing mutable variables and loops with powerful ways to define and compose functions."

Commentary. Although functional programming is covered in the Programming Languages listing above, this course is a useful extension. It teaches Scala, a relatively new language that neatly integrates object orientation with functional programming and includes support for concurrency, running on the Java Virtual Machine and by many accounts improving on Java. It has been adopted by a number of web startups, including Twitter, LinkedIn, and Coursera itself. Scala has a relatively steep learning curve, but its power, versatility, varied features, and sophisticated underpinnings make this course essentially an advanced tutorial in programming languages, with a well-integrated set of programming assignments. The instructor is the developer of Scala, and his lectures are both precise and effective.

Evaluation.

Course Level	Hours per Week	Course Grade				Student Grade	Curve
		Lectures	Homework	Assessment	Overall		Pass/"A"
Intermediate	10	94	93	97	95	97.1	60/80

Prerequisites. Solid programming experience. Programming Languages would be excellent preparation, but is by no means essential.

Textbook. The instructor's textbook is the standard reference and is highly recommended [43], though not required.

Going Further. Coursera also offers the follow-on course to this one, "Principles of Reactive Programming" (https://www.coursera.org/course/reactive). It features aspects of Scala that bear on concurrency for web services and distributed systems.

Databases 💻

(See listing in Data Sciences Department.)

Computer Networks 💻✓

David Wetherall, Arvind Krishnamurthy, and John Zahorjan, University of Washington "Computer Networks" (Coursera, Winter 2014: https://www.coursera.org/course/comnetworks)

"This course introduces the fundamental problems of computer networking, from sending bits over wires to running distributed applications. For each problem, we explore the design strategies that have proven valuable in practice. Topics include error detection and correction, multiple-access, bandwidth allocation, routing, internetworking, reliability, quality of service, naming, content delivery, and security."

Commentary. An understanding of the networks on which we depend is important for everyone, but particularly for bioinformatics practitioners who may very well find themselves moving around extremely large datasets. There are optional hands-on exercises in addition to the extensive homework and relatively challenging exams.

Evaluation.

Course Level	Hours per Week	Course Grade				Student Grade	Curve
		Lectures	Homework	Assessment	Overall		Pass/"A"
Intermediate	6	93	91	89	92	87.4	50/?

Prerequisites: Introduction to Computer Science.

Textbook. *Computer Networks*, an updated classic by Andrew Tanenbaum and the instructor [44], is recommended but not required. It is not cheap, and there are several free alternatives. One is by Ivan Marsic of Rutgers University (http://www.ece.rutgers.edu/~marsic/books/CN), and another by Olivier Bonaventure of the Université Catholique de Louvain (http://cnp3bis.info.ucl.ac.be).

Alternatives: Stanford has a similar MOOC, "Introduction to Computer Networking" by Philip Levis and Nick McKeown (https://class.stanford.edu/courses/Engineering/Networking/Winter2014/about).

Going Further. Coursera covers an advanced topic in this area, "Software-Defined Networking" by Nick Feamster of the Georgia Institute of Technology (https://www.coursera.org/course/sdn).

Artificial Intelligence 💻✓

Dan Klein, University of California, Berkeley "Artificial Intelligence" (edX, Winter 2013: https://www.edx.org/course/uc-berkeleyx/uc-berkeleyx-cs188-1x-artificial-579)

> *"CS188.1x focuses on Behavior from Computation. It will introduce the basic ideas and techniques underlying the design of intelligent computer systems. A specific emphasis will be on the statistical and decision–theoretic modeling paradigm. By the end of this course, you will have built autonomous agents that efficiently make decisions in stochastic and in adversarial settings."*

Commentary. This course was recommended in the last catalog when it was on iTunes U, but it has now been partially ported to edX. It is a very well produced treatment of heuristic search in all its many variations. The programming assignments (in Python) are stimulating and just plain fun. The second part of the course, yet to appear, will cover reasoning and learning, and thus may overlap more with other courses in the Data Sciences Department.

Evaluation.

Course Level	*Hours per Week*	*Course Grade*				*Student Grade*	*Curve*
		Lectures	*Homework*	*Assessment*	*Overall*		*Pass/"A"*
Intermediate	10	97	98	96	97	92	50/80

Personal Note. The author worked in AI during its boom in the 1980s, building expert systems based on semantic networks in an industrial R&D setting. While the subsequent "AI Winter" deflated much of the hype surrounding the field, in fact AI spawned Machine Learning, Natural Language Processing, and many other threads of modern computer science, while contributing greatly to bioinformatics in its earlier days. In particular, The International Society for Computational Biology arose out of the meeting series Intelligent Systems for Molecular Biology (ISMB), which in turn was founded by three AI workers: Larry Hunter, Jude Shavlik, and the author [45]. For more of the AI perspective on bioinformatics, see a recent talk by Larry Hunter at a meeting of the American Association for Artificial Intelligence, entitled "Building a Mind for Life," at http://videolectures.net/aaai2013_hunter_building_mind.

Prerequisites: Knowledge of object-oriented programming, recursion, Python, data structures.

Textbook. *Artificial Intelligence: A Modern Approach* by Stuart Russell and Peter Norvig [46] (though the course is largely self-contained). It is pricey, but is by far the dominant book in the field and covers much more material than does the MOOC.

Alternatives. A version of the Artificial Intelligence course that gave rise to the MOOC movement, taught by Google's Peter Norvig and Stanford's Sebastian Thrun in 2011, is now available through the latter's startup, Udacity (https://www.udacity.com/course/cs271). MIT OpenCourseWare has "Artificial Intelligence" (http://ocw.mit.edu/courses/electrical-engineering-and-computer-science/6-034-artificial-intelligence-fall-2010) taught by AI pioneer Patrick Winston and featuring interactive demonstrations. See the previous catalog for other alternatives.

Going Further. Those impatient for part 2 of the course may want to peek at the non-MOOC version on iTunes U (http://itunes.apple.com/WebObjects/MZStore.woa/wa/viewPodcast?id=496298636). A more in-depth examination of planning can be found in "Artificial Intelligence Planning" from Gerhard Wickler and Austin Tate of the University of Edinburgh (https://www.coursera.org/course/aiplan).

Machine Learning 💻✓

(See listing in Data Sciences Department.)

Natural Language Processing 💻

Michael Collins, Columbia University "Natural Language Processing" (Coursera, Spring 2013: https://www.coursera.org/course/nlangp)

> *"In this course you will study mathematical and computational models of language, and the application of these models to key problems in natural language processing. The course has a focus on machine learning methods, which are widely used in modern NLP systems: we will cover formalisms such as hidden Markov models, probabilistic context-free grammars, log-linear models, and statistical models for machine translation."*

Commentary. Natural Language Processing (NLP) technology can be considered the "high end" of text mining and is important to the extraction of information from the biological literature. For instance, finding the genes or proteins identified in publications is a prototypical challenge for named-entity recognition techniques from NLP. As noted elsewhere, NLP also tends to use statistical learning techniques, particularly hidden Markov models, which are both important in bioinformatics and neglected in the introductory Machine Learning courses.

Personal Note. The author did a Master's degree in computer science at Penn (in the same department in which the instructor above did his PhD) with a concentration in computational linguistics. That exposure determined the author's future research program, which has been based on the notion that grammars and parsing are relevant to describing gene and macromolecular structure, and in particular RNA folding, where the stochastic context-free grammars taught in this course are now widely used. Thus, NLP-related technologies can apply to much more than text mining in biology.

Prerequisites. Calculus, Probability, programming experience.

Textbook. None required. As noted in the previous catalog, the book *Natural Language Processing with Python* by Steven Bird, Ewan Klein, and Edward Loper [47], which is freely available online (http://www.nltk.org/book), is an excellent resource for self-study in conjunction with the powerful open source supporting library for NLP and text analytics called NLTK (Natural Language Toolkit, http://www.nltk.org). Marcus Kracht, now at the University of Beilefeld, made beautiful lecture notes for courses he taught at the University of California, Los Angeles including an "Introduction to Computational Linguistics" (http://wwwhomes.uni-bielefeld.de/mkracht/html/compling-intro.pdf) as well as an "Introduction to Probability Theory and Statistics for Linguistics" (http://wwwhomes.uni-bielefeld.de/mkracht/html/statistics.pdf).

Alternatives. Coursera also lists an NLP course from Dan Jurafsky and Christopher Manning of Stanford, recommended in the previous catalog (https://www.coursera.org/course/nlp). However, as of this writing it has still not been scheduled, despite the lectures being available in preview form. Some students who have looked at both courses have expressed a preference for the one from Stanford, but the coverage looks very similar.

Going Further. The Johns Hopkins Center for Language and Speech Processing (CLSP) has an advanced seminar series and Summer School with online videos (http://videolectures.net/jhclsp).

Robotics

Frank Park, Seoul National University "Robot Mechanics, Planning, and Control" Parts 1 and 2 (edX, Spring/Summer 2014: https://www.edx.org/course/snux/snux-snu446-345-1x-robot-mechanics-1529 and https://www.edx.org/course/snux/snux-snu446-345-2x-robot-mechanics-1700)

> *"This course provides a rigorous introduction to hand grasp analysis, kinematics of open and closed chains, trajectory generation, motion planning, and control of robots. The emphasis is not on the latest research trends and technological innovations in robotics, content that risks becoming obsolete in a matter of years. Rather, the focus is on learning the fundamental concepts and core principles that underlie robotics as a scientific discipline. The intent is to help students acquire a unified set of analytical tools for the modeling, planning, and control of robots, together with a reliable physical intuition that recognizes the unique and interdisciplinary nature of robotics; in short, content that will serve as a reliable foundation for whatever trends may appear later, and remain relevant to both the practitioner and researcher."*

Commentary. Beginning with the highly automated sequencing centers built for the final push in the human genome sequencing effort, robotics has had a significant role in biological "Big Science." More recently this has extended to structural genomics [48] and epigenetics [49], and of course automation has long been important in high-throughput screening for drug discovery. This course offers the opportunity to learn control and planning principles that are likely to underlie more and more future biological data harvesting, and may also be relevant to certain aspects of the neurosciences.

Prerequisites. Calculus, Linear Algebra, Differential Equations.

Textbook. Lecture notes are provided. The course notes by Franz Hover of the Massachusetts Institute of Technology for his course "Design of Electromechanical Robotic Systems" are extensive (http://ocw.mit.edu/courses/mechanical-engineering/2-017j-design-of-electromechanical-robotic-systems-fall-2009/course-text).

Alternatives. Coursera has "Control of Mobile Robots" by Magnus Egerstedt of the Georgia Institute of Technology (https://www.coursera.org/course/conrob), and edX offers "Autonomous Navigation for Flying Robots" by Jürgen Sturn and Daniel Cremers of the Technische Universität München (https://www.edx.org/course/tumx/tumx-autonavx-autonomous-navigation-1658). Mobile robots are all the rage, what with autonomous cars and helicopters, though they are perhaps less relevant to biology than more conventional industrial automation. Also, Stanford's Oussama Khatib has an "Introduction to Robotics" on iTunes U (https://itunes.apple.com/us/itunes-u/introduction-to-robotics/id384233063).

Discrete Optimization

Pascal Van Hentenryck, The University of Melbourne "Discrete Optimization" (Coursera, Spring 2014: https://www.coursera.org/course/optimization)

> *"This class is an introduction to discrete optimization and exposes students to some of the most fundamental concepts and algorithms in the field. It covers constraint programming, local search, and mixed-integer programming from their foundations to their applications for complex practical problems in areas such as scheduling, vehicle routing, supply-chain optimization, and resource allocation."*

Commentary. The Linear Programming course listed in the Mathematics Department treats the optimization of continuous variables, though it also touches on the discrete case in its coverage of integer programming. This course goes much further in its approach to discrete problems, which feel a lot more like computer science than applied math, requiring clever algorithmic solutions. The lectures

are entertaining but substantive, delivered by a pioneer of modern constraint programming systems. The programming assignments are challenging and much more free form than is typical of computational MOOCs, allowing students very wide latitude and relatively little guidance.

Prerequisites. Programming skills, Algorithms, Linear Algebra.

Textbook. None is required.

Alternatives. See the previous catalog.

Computer Graphics

Ravi Ramamoorthi, University of California, Berkeley "Foundations of Computer Graphics" (edX, Fall 2013: https://www.edx.org/course/uc-berkeleyx/uc-berkeleyx-cs-184-1x-foundations-1003)

> *"Students will understand the concepts of 3D graphics, and develop programs that create images of a 3D scene with lighting, using both real-time OpenGL and GLSL programming, as well as offline raytracing. The focus is on foundations and writing your own programs, rather than learning use of ... specific software"*

Commentary. This is a fairly brisk introduction but will serve to get one started in a field that bears on bioinformatics in areas such as the depiction of biological structures [50], animations of cellular processes [51], and general scientific visualization.

Prerequisites. C/C++ programming skills. Linear Algebra helpful.

Textbook. Besides OpenGL reference books, *Fundamentals of Computer Graphics* by Peter Shirley, Michael Ashikhmin, and Steve Marschner [52] is recommended but not required.

Alternatives. The previous catalog offered a number of alternatives, some of which appear to delve more into theory and others that cover GPUs, but none of them are MOOCs, so that their programming exercises are unlikely to be helpful. Udacity has a course by Eric Haines in "Interactive 3D Graphics" (https://www.udacity.com/course/cs291) that uses WebGL for the programming assignments.

Going Further. The previous catalog also lists a number of advanced non-MOOC computer graphics courses from the University of California, Davis, and they have now added a new one, "Ray Tracing for Global Illumination" by Nelson Max (https://itunes.apple.com/us/itunes-u/ray-tracing-for-global-illumination/id565677048).

Image Processing

Guillermo Sapiro, Duke University "Image and Video Processing: From Mars to Hollywood with a Stop at the Hospital" (Coursera, Winter 2014: https://www.coursera.org/course/images)

> *"The course will start with an introduction to the basics of image formation and the fundamental concepts that translate a physical scene into a digital image. We will then describe the underlying concepts of image compression, the enabling technology that makes it possible for images to be sent from Mars and videos to be stored in our mobile phones. We will cover the most fundamental tools in image enhancement, showing how simple tools can significantly improve images. Both geometric and non-geometric tools as well as spatial and non-spatial operations will be presented. Details on image segmentation will be provided, one of the most fundamental and useful problems in image processing."*

Commentary. Despite the instructor's whimsical title, this is a red-meat technical course in a topic that is important in areas including biomedical imaging, omic technologies such as microarrays, certain next-generation sequencing platforms, and high-content screening that involves image processing of cell-based assays. Brain imaging and virus structural analysis are covered in the final week.

Prerequisites. Differential Equations, Linear Algebra, and Signal Processing helpful.

Textbook. *Digital Image Processing* by Rafael Gonzalez and Richard Woods [53] is suggested, but it only covers half the course and is expensive. The alternative Coursera course below is self-contained.

Alternatives. Coursera also has a similar course, "Fundamentals of Digital Image and Video Processing" by Aggelos Katsaggelos of Northwestern University (https://www.coursera.org/course/digital). The University of California at Davis has a non-MOOC "Image Processing and Analysis" by Owen Carmichael (https://itunes.apple.com/us/itunes-u/image-processing-analysis/id458753849).

Going Further. Nikos Paragios and Pawan Kumar of the École Centrale Paris teach "Discrete Inference and Learning in Artificial Vision" (https://www.coursera.org/course/artificialvision) on Coursera, addressing machine learning approaches to image analysis. A particular application of image processing related to biology is "Biometrics," taught by Phalguni Gupta of the Indian Institute of Technology, Kanpur (http://nptel.ac.in/courses/106104119). For other applications, see Biomedical Imaging in the Translational Sciences Department.

Computer Science Seminars

(See seminars at http://www.youtube.com/playlist?list=PL9s5HIfOWP3Mf-vearKDcXavZtiv2uk9T)

Data Science Department

This new department serves to bring together courses that would otherwise be listed primarily under the Computer Science and Mathematics Departments, but which together form a coherent foundation for approaching large datasets of the sort seen in bioinformatics. These subjects include Databases, of course, but we also place here various forms of Machine Learning and Statistics, as well as cross-listing a number of courses for their relevance to various forms of data mining and analytics.

Introduction to Data Science 💻✓

Bill Howe, University of Washington "Introduction to Data Science" (Coursera, Summer 2013: https://www.coursera.org/course/datasci)

> *"Commerce and research is being transformed by data-driven discovery and prediction. Skills required for data analytics at massive levels – scalable data management on and off the cloud, parallel algorithms, statistical modeling, and proficiency with a complex ecosystem of tools and platforms – span a variety of disciplines and are not easy to obtain through conventional curricula. Tour the basic techniques of data science, including both SQL and NoSQL solutions for massive data management (e.g., MapReduce and contemporaries), algorithms for data mining (e.g., clustering and association rule mining), and basic statistical modeling (e.g., linear and non-linear regression)."*

Commentary. The subject matter for this course is foundational to current practice in bioinformatics. The delivery is rather informal, and the first iteration of the course hit a number of snags, especially as regards the programming assignments, which used Python, SQL, and R. There is also a Kaggle competition, mining of the raw Twitter feed (which arrives at a truly alarming rate), and optional assignments using Amazon Web Services. As the instructor points out, data mining from the web can be a messy process, and the experience is worthwhile even when somewhat ragged.

Evaluation.

Course Level	*Hours per Week*	*Course Grade*				*Student Grade*	*Curve*
		Lectures	*Homework*	*Assessment*	*Overall*		*Pass/"A"*
Introductory	6	85	84	82	85	93.6	75/?

Prerequisites. Databases and programming experience.

Textbook. *Mining of Massive Datasets* by Jeffrey Ullman and Anand Rajaraman [54] is recommended but not required. See the Big Data course below for further description of it.

Alternatives. This course should be seen as an overview that provides a hands-on sampling of many aspects of data science. An alternative is "Intro to Data Science" by Dave Holtz on Udacity (https://www.udacity.com/course/ud359). Another approach, requiring a significantly greater investment of time, would be the comprehensive Data Science course listed immediately below. Yet another path would be to pursue a selection of the more in-depth courses in the remainder of this Department's listing after taking this course as an introduction.

Data Science 💻

Brian Caffo, Jeff Leek, and Roger Peng, Johns Hopkins University "Data Science" (Coursera, Spring/Summer 2014: https://www.coursera.org/specialization/jhudatascience/1)

"Formulate context-relevant questions and hypotheses to drive data scientific research… Identify, obtain, and transform a data set to make it suitable for the production of statistical evidence communicated in written form… Build models based on new data types, experimental design, and statistical inference."

Commentary. This is actually a collection of short courses, comprising a Coursera Specialization, which run in three concurrent blocks of three courses each. They include "The Data Scientist's Toolbox," "R Programming," and "Getting and Cleaning Data" in the first block; "Exploratory Data Analysis," "Reproducible Research," and "Statistical Inference" in the second block; and "Regression Models," "Practical Machine Learning," and "Developing Data Products" in the third block. These may be followed by a "capstone" project to create a "usable/public data product." The courses can also be taken individually. Together they constitute a fairly comprehensive course of study in data science at an intermediate level.

Prerequisites. Programming experience.

Textbook. See individual courses in the Specialization.

Alternatives. Udacity also has a data science track with introductory study in computer science and statistics, then courses "Intro to Data Science," "Exploratory Data Analysis," "Intro to Hadoop and MapReduce," and "Data Wrangling with MongoDB" (https://www.udacity.com/courses#!/data-science).

Data Analytics

Dimitris Bertsimas and Allison O'Hair, The Massachusetts Institute of Technology "The Analytics Edge" (edX, Spring 2014: https://www.edx.org/course/mitx/mitx-15-071x-analytics-edge-1416)

"In this course, you will learn how to use data and analytics to give an edge to your career and your life. We will examine real world examples of how analytics have been used to significantly improve a business or industry. These examples include Moneyball, eHarmony, the Framingham Heart Study, Twitter, IBM Watson, and Netflix. Through these examples and many more, we will teach you the following analytics methods: linear regression, logistic regression, trees, text analytics, clustering, visualization, and optimization."

Commentary. Once again, this course overlaps with the previous ones, but is listed primarily because it offers a very different viewpoint. Arising as it does in MIT's famed Sloan School of Management, it provides a business perspective on data mining, which in such a context is generally termed data analytics and is considered an aspect of Operations Research. Otherwise, the tools are much the same, starting with the use of the R statistical programming language.

Prerequisites. Calculus.

Textbook. None is required.

Alternatives. Data analytics from an economic perspective is offered in Coursera's "Core Concepts in Data Analysis" from Boris Mirkin of the Higher School of Economics of the Russian National Technical University (https://www.coursera.org/course/datan). For yet another take on data analytics that is completely out of left field, consider the edX offering "Sabermetrics 101: Introduction to Baseball Analytics" by Andy Andres of Boston University (https://www.edx.org/course/bux/bux-sabr101x-sabermetrics-101-1558).

Big Data ✓

Gautam Shroff, Indian Institute of Technology, Delhi "Web Intelligence and Big Data" (Coursera, Fall 2012: https://www.coursera.org/course/bigdata)

"Introduction and Overview… Look: Search, Indexing and Memory… Listen: Streams, Information and Language, Analyzing Sentiment and Intent… Load: Databases and their Evolution, Big Data Technology and Trends… Programming: Map-Reduce… Learn: Classification, Clustering, and Mining, Information Extraction… Connect: Reasoning: Logic and its Limits, Dealing with Uncertainty… Programming: Bayesian Inference for Medical Diagnostics… Predict: Forecasting, Neural Models, Deep Learning, and Research Topics… Data Analysis: Regression and Feature Selection."

Commentary. This course provides a taste of technologies, methods and issues surrounding the maintenance and mining of big data, as well as cloud computing. Again it overlaps significantly with courses above, but it is listed separately because it provides a framework to follow closely the excellent recommended textbooks, described below. Following these books assiduously lends an otherwise introductory course a solid base of theory, covering important topics like locality-sensitive hashing, Bloom filters, and latent semantic indexing.

Evaluation.

Course Level	*Hours per Week*	*Course Grade*				*Student Grade*	*Curve*
		Lectures	*Homework*	*Assessment*	*Overall*		*Pass/"A"*
Intermediate	8	85	87	85	85	90.7	?/80

Prerequisites. Databases and programming experience.

Textbook. *Mining of Massive Datasets* by Jeffrey Ullman and Anand Rajaraman [54] (http://infolab.stanford.edu/~ullman/mmds.html) and *Introduction to Information Retrieval* by Christopher Manning, Prabhakar Raghavan, and Hinrich Schütze [55] (http://nlp.stanford.edu/IR-book/information-retrieval-book.html), both of which are available from Stanford in free online versions at the links indicated.

Databases

Jennifer Widom, Stanford University "Introduction to Databases" (Stanford Online, Winter 2014: https://class.stanford.edu/courses/Engineering/db/2014_1/about or Coursera, self-study: https://www.coursera.org/course/db)

"This course covers database design and the use of database management systems for applications. It includes extensive coverage of the relational model, relational algebra, and SQL. It also covers XML data including DTDs and XML Schema for validation, and the query and transformation languages XPath, XQuery, and XSLT. The course includes database design in UML, and relational design principles based on dependencies and normal forms. Many additional key database topics from the design and application-building perspective are also covered: indexes, views, transactions, authorization, integrity constraints, triggers, on-line analytical processing (OLAP), JSON, and emerging 'NoSQL' systems."

Commentary. This course is offered live at the Stanford Online site, which uses the edX platform, or as a self-study course on Coursera. Aside from providing an interesting head-to-head comparison of the competing learning environments, the material in this course is *sine qua non* for bioinformatics.

Prerequisites. Introduction to Computer Science.

Textbook. None is required, but the lectures are keyed to four popular textbooks.

Alternatives. See the previous catalog. However, given that this course is quite comprehensive as well as being available for self-study, there should be little need for alternatives.

Metadata

Jeffrey Pomerantz, University of North Carolina at Chapel Hill "Metadata: Organizing and Discovering Information" (Coursera, Summer 2014: https://www.coursera.org/course/metadata)

> *"Metadata—or data about data—describes real and digital objects, so that those objects may be organized now and found later… Metadata is a tool that enables the information age functions performed by humans as well as those performed by computers. Metadata is important to many fields, particularly Computer Science; but this course is not purely a Computer Science course. This course approaches Metadata from the perspective of Information Science, which is a broad interdisciplinary field that studies how people create and manage information."*

Commentary. A number of fields lay claim to the term "information" and library science is one of them. This course presents that perspective, offering a high-level view of metadata schemas and standards. It will be of interest to those concerned with such standards as the Dublin Core or the "minimum information" schemas widely used in bioinformatics.

Prerequisites. Knowledge of HTML or HTML5.

Textbook. None is required, but *Introduction to Metadata* by Murtha Baca [56] is recommended.

Statistics

Mine Çetinkaya-Rundel, Duke University "Data Analysis and Statistical Inference" (Coursera, Spring 2014: https://www.coursera.org/course/statistics)

> *"This course introduces you to the discipline of statistics as a science of understanding and analyzing data. You will learn how to effectively make use of data in the face of uncertainty: how to collect data, how to analyze data, and how to use data to make inferences and conclusions about real world phenomena."*

Commentary. There is a plethora of online alternatives for this subject (see below), but this one appears to be both reasonably rigorous and closely tied to a free online textbook, below. The course has labs and a final project that use R and RStudio.

Textbook. *OpenIntro Statistics* is a free online textbook (http://www.openintro.org/stat/textbook.php) co-authored by the instructor along with David Diez of Google and Christopher Barr of Harvard.

Alternatives. Coursera has a number of alternatives. A similar course by Alison Gibbs and Jeffrey Rosenthal of the University of Toronto is entitled "Statistics: Making Sense of Data" (https://www.coursera.org/course/introstats). Princeton offers an introductory "Statistics One" by Andrew Conway (https://www.coursera.org/course/stats1). All the courses above use R, which is fairly standard in bioinformatics applications, but for those who may want to use the SAS package (more associated with clinical trials and the pharma industry) there is "Passion Driven Statistics" by Lisa Dierker of Wesleyan University (https://www.coursera.org/course/pdstatistics). edX offers the three-part "Introduction to Statistics" series from Berkeley ("Descriptive Statistics," "Probability," and "Inference") by Ani Adhikari and Philip Stark (https://www.edx.org/course/uc-berkeleyx/uc-berkeleyx-stat2-1x-introduction-1138, https://www.edx.org/course/uc-berkeleyx/uc-berkeleyx-stat2-2x-introduction-685, and https://www.edx.org/course/uc-berkeleyx/uc-berkeleyx-stat2-3x-introduction-825). Also on edX is "Explore Statistics with R" by Andreas Montelius, Peter Lönnerberg, Mikael Huss, and Matilda Utbolt of the Karolinska Institute (https://www.edx.org/course/kix/kix-kiexplorx-explore-statistics-r-1524). Finally, Udacity has an "Introduction to Statistics" (https://www.udacity.com/course/st101) by Sebastian Thrun and another course "Statistics" by Sean

Laraway and Ronald Rogers of San Jose State University (https://www.udacity.com/course/st095). Still other alternatives are listed in the previous catalog.

Going Further. The course Epidemiology and Biostatistics in the Translational Sciences Department provides another application of statistics with its own unique character and conventions. See also the Statistical Learning course in the Data Sciences Department and Mathematical Biostatistics immediately below.

Mathematical Biostatistics 💻✓

Brian Caffo, Johns Hopkins University "Mathematical Biostatistics Boot Camp" Parts 1 and 2 (Coursera, Spring 2014: https://www.coursera.org/course/biostats and https://www.coursera.org/course/biostats2)

> *[Part 1] "This course puts forward key mathematical and statistical topics to help students understand biostatistics at a deeper level... Topics include probability, random variables, distributions, expectations, variances, independence, conditional probabilities, likelihood and some basic inferences based on confidence intervals." [Part 2] "This class presents fundamental concepts in data analysis and statistical inference, focusing on one and two independent samples. Students having taken this class should be able to summarize samples, perform relevant hypothesis tests and perform a collection of two sample comparisons. Classical non-parametric methods and discrete data analysis methods are discussed."*

Commentary. The fundamentals of statistics are no different for biology than for any other field, but inevitably the exact material covered and emphasized as well as the examples used are flavored by the domain, so this course is listed as an alternative to the one immediately above. In "boot camp" fashion it moves along at a faster clip than the previous course, and is somewhat less polished, but may be preferred by those who are reviewing or extending their knowledge of statistics rather than seeing it for the first time. The R language is used for exercises.

Evaluation. (Part 1 only)

Course Level	*Hours per Week*	*Course Grade*				*Student Grade*	*Curve*
		Lectures	*Homework*	*Assessment*	*Overall*		*Pass/"A"*
Introductory	8	87	90	92	89	100.0	?/90

Prerequisites. Calculus and familiarity with set theory.

Textbook. The MOOC points to the "Methods in Biostatistics I" course resource on the Johns Hopkins School of Public Health Open Courseware site, which in turn lists additional textbooks and resources (http://ocw.jhsph.edu/index.cfm/go/viewCourse/course/MethodsInBiostatisticsI/coursePage/index).

Alternatives. Besides the general Statistics course immediately above, the Epidemiology and Biostatistics course in the Translational Sciences Department provides an introduction to statistics tailored to clinical trials and public health studies.

Statistical Programming 💻✓

Roger Peng, Johns Hopkins University "Computing for Data Analysis" (Coursera, Winter 2013: https://www.coursera.org/course/compdata)

> *"In this course you will learn how to program in R and how to use R for effective data analysis. You will learn how to install and configure software necessary for a statistical programming environment, discuss generic programming language concepts as they are implemented in a high-level statistical language. The course covers practical issues in statistical computing which includes programming in R, reading data into R, creating informative data graphics, accessing R packages, creating R packages*

with documentation, writing R functions, debugging, and organizing and commenting R code. Topics in statistical data analysis and optimization will provide working examples."

Commentary. As was evident in the Statistics listing above, most courses in this Department dealing in statistics offer some training with the R language. This entry is offered as a more or less "pure play" course in R, short and to the point, for those who don't feel the need for the rest of the statistics material. It is essentially one of the components of the Johns Hopkins Specialization in Data Science described above, and is taken by first-year graduate students in Biostatistics at that institution.

Evaluation.

Course Level	*Hours per Week*	*Course Grade*				*Student Grade*	*Curve*
		Lectures	*Homework*	*Assessment*	*Overall*		*Pass/"A"*
Introductory	4	88	87	85	88	99.0	?/90

Prerequisites. Basic knowledge of statistics and programming experience.

Textbook. There are any number of R and S language books, several of which are listed in the course description. An exhaustive list can be found on the R Project web site, along with the freely available manual (http://www.r-project.org).

Alternatives. Jeff Leek, a colleague of the instructor above at Johns Hopkins, offers a more wide-ranging course that provides a conceptual introduction and covers not only R but Rstudio, markdown, version control, git, and Github (https://class.coursera.org/datascitoolbox). It is also part of the Data Science Specialization on Coursera, and so would be subsumed under the Data Science course above, as would a number of topics in this Department.

Machine Learning ✓

Andrew Ng, Stanford University "Machine Learning" (Coursera, Fall 2013: https://www.coursera.org/course/ml)

"This course provides a broad introduction to machine learning, datamining, and statistical pattern recognition. Topics include: (i) Supervised learning (parametric/non-parametric algorithms, support vector machines, kernels, neural networks). (ii) Unsupervised learning (clustering, dimensionality reduction, recommender systems, deep learning). (iii) Best practices in machine learning (bias/variance theory; innovation process in machine learning and AI)."

Commentary. Although there are a number of alternative courses of similar quality available (see below), this instructor is one of the co-founders of Coursera and merits pride of place. The course is nicely constructed conceptually, using linear regression as a foundation and elaborating other methods from there. There is a well-placed emphasis on practical advice over theory, and the programming assignments (using Octave) work well, largely thanks to extensive documentation.

Evaluation.

Course Level	*Hours per Week*	*Course Grade*				*Student Grade*	*Curve*
		Lectures	*Homework*	*Assessment*	*Overall*		*Pass/"A"*
Introductory	8	94	90	93	93	100.0	80/—

Prerequisites. Introduction to Computer Science.

Textbook. None is required. The instructor has elsewhere posted a full set of course notes for the Stanford version of the course, which has somewhat more detail than the material in the MOOC (http://see.stanford.edu/see/materials/aimlcs229/handouts.aspx).

Alternatives. Coursera also offers a course of the same name from Pedro Domingos of the University of Washington (https://www.coursera.org/course/machlearning). It has somewhat broader coverage, treating decision trees, rule induction, and even learning theory, and may be preferred by some for that reason. The course by CalTech's Yaser Abu-Mostafa that was suggested in the previous catalog is now offered as an edX entry (https://www.edx.org/course/caltechx/caltechx-cs1156x-learning-data-1120), and is also still available directly from CalTech for self-study (http://work.caltech.edu/telecourse.html). Finally, see the Statistical Learning course below for yet another alternative with a different perspective on the field.

Going Further. Purdue University hosted a "Machine Learning Summer School" in 2011 (http://www.youtube.com/playlist?list=PL2A65507F7D725EFB) from which over sixty full-length talks are available on specialized topics. Also see the Going Further section under the Statistical Learning course below.

Statistical Learning 💻✓

Trevor Hastie and Rob Tibshirani, Stanford University "Statistical Learning" (Stanford Online, Winter 2014: https://class.stanford.edu/courses/HumanitiesScience/StatLearning/Winter2014/about)

> *"This is an introductory-level course in supervised learning, with a focus on regression and classification methods. The syllabus includes: linear and polynomial regression, logistic regression and linear discriminant analysis; cross-validation and the bootstrap, model selection and regularization methods (ridge and lasso); nonlinear models, splines and generalized additive models; tree-based methods, random forests and boosting; support-vector machines. Some unsupervised learning methods are discussed: principal components and clustering (k-means and hierarchical)."*

Commentary. Machine learning was independently "invented" by the computer science and statistics communities, and while the Machine Learning course above presents the former perspective, this one is taught by statisticians. As such, there is more emphasis on models of data and their characterization, as well as on methods such as the bootstrap, lasso, random forests, and boosting. While the two approaches overlap considerably, it is well worth taking Statistical Learning as a follow-on to Machine Learning for the sake of the additional perspective, as reinforcement for the basic methodologies, and because it is taught using R rather than Octave (the former language being more commonly used in bioinformatics). The statistical approach also provides more of a mathematical foundation, though this course purports not to be "math-heavy." The instructors are pioneers in statistical learning, and co-authors of the standard math-heavy text in the field (see below). The instructors teach as a team, and they have a wonderfully relaxed rapport, particularly when they interview their illustrious mentors and involve their own students. Despite the detailed demonstrations in R, no major programming assignments are set, and the assessments are not very stringent.

Evaluation.

Course Level	*Hours per Week*	*Course Grade*				*Student Grade*	*Curve*
		Lectures	*Homework*	*Assessment*	*Overall*		*Pass/"A"*
Introductory	6	97	84	85	92	96	50/—

Prerequisites. Statistics, Linear Algebra, and programming experience.

Textbook. *An Introduction to Statistical Learning* by Gareth James, Daniela Witten, and the instructors [57], is available as a free online text (http://www-bcf.usc.edu/~gareth/ISL). This is a simplified version of the instructors' (with Jerome Friedman) classic *The Elements of Statistical Learning* [58], also available as a free download (http://statweb.stanford.edu/~tibs/ElemStatLearn). Both books, and the course itself, make use of gene expression data in examples.

Going Further. Computational biologists will want to supplement these introductory courses with self-study in certain methods that have proven useful in bioinformatics, such as Expectation Maximization (EM) and especially Hidden Markov Models (HMMs), which are ubiquitous in the field. Many of these additional statistical learning methods relevant to bioinformatics are covered in the "Graphical Models" and "Natural Language Processing" courses below; see the previous catalog for other suggestions along these lines.

Graphical Models 💻✓

Daphne Koller, Stanford University "Probabilistic Graphical Models" (Coursera, Spring 2013: https://www.coursera.org/course/pgm)

> *"Probability theory gives us the basic foundation to model our beliefs about the different possible states of the world, and to update these beliefs as new evidence is obtained. These beliefs can be combined with individual preferences to help guide our actions, and even in selecting which observations to make. While probability theory has existed since the 17th century, our ability to use it effectively on large problems involving many inter-related variables is fairly recent, and is due largely to the development of a framework known as Probabilistic Graphical Models (PGMs). This framework, which spans methods such as Bayesian networks and Markov random fields, uses ideas from discrete data structures in computer science to efficiently encode and manipulate probability distributions over high-dimensional spaces, often involving hundreds or even many thousands of variables."*

Commentary. This course is very energetically taught by the other co-founder of Coursera and its most visible proponent, Daphne Koller. Probabilistic graphical models are particularly relevant to complex interconnected biological datasets, making this an important elective. The course covers a lot of ground at a fast pace; in the words of the instructor "this is a very challenging class (the Stanford version is notorious also)" and involves "a ton of work." In addition to a Basic Track that includes the problem sets and exams, students can opt for an Advanced Track that includes a set of programming assignments using MATLAB or Octave that can be quite formidable, though they are fairly well documented.

Evaluation. (Advanced Track)

Course Level	*Hours per Week*	*Course Grade*				*Student Grade*	*Curve*
		Lectures	*Homework*	*Assessment*	*Overall*		*Pass/"A"*
Intermediate	12	93	92	91	92	90.3	70/—

Prerequisites. Machine Learning and programming experience. Probability would be very helpful.

Testbook. *Probabilistic Graphical Models* by the instructor is optional (and massive, at 1280 pages) [59].

Neural Networks 💻

Geoffrey Hinton, University of Toronto "Neural Networks for Machine Learning" (Coursera, Fall 2012: https://www.coursera.org/course/neuralnets)

> *"Neural networks use learning algorithms that are inspired by our understanding of how the brain learns, but they are evaluated by how well they work for practical applications such as speech recognition, object recognition, image retrieval and the ability to recommend products that a user will like. As computers become more powerful, Neural Networks are gradually taking over from simpler Machine Learning methods. They are already at the heart of a new generation of speech recognition devices and they are beginning to outperform earlier systems for recognizing objects in images. The course will explain the new learning procedures that are responsible for these advances, including*

effective new procedures for learning multiple layers of non-linear features, and give you the skills and understanding required to apply these procedures in many other domains."

Commentary. Neural networks, which were very popular at the time bioinformatics was first gathering momentum but then fell out of fashion for a decade or so, are back again with a vengeance, based on recent successes in areas like speech recognition. The instructor is one of the pioneers of the field, having co-invented backpropagation, Boltzmann machines, and other innovations. This course includes challenging programming assignments requiring MATLAB, Octave, or Python.

Prerequisites. Calculus, Linear Algebra, Machine Learning, Probabiity, and programming experience.

Natural Language Processing

(See listing in Computer Science Department.)

Computer Graphics

(See listing in Computer Science Department.)

Visualization

Katy Börner, Indiana University "Information Visualization MOOC" (Indiana University, 2014: http://ivmooc.cns.iu.edu)

"This course provides an overview about the state of the art in information visualization. It teaches the process of producing effective visualizations that take the needs of users into account. Among other topics, the course covers: Data analysis algorithms that enable extraction of patterns and trends in data… Major temporal, geospatial, topical, and network visualization techniques… Discussions of systems that drive research and development."

Commentary. The Cyberinfrastructure for Network Science Center at Indiana University has fielded this MOOC as an independent effort for several years running, under the leadership of Katy Börner (although a PhD student, Scott Weingart, is teaching the 2014 edition). The course covers visual mapping of temporal, geospatial, and topical information as well as network analysis. For visualization techniques specific to biological data, see the Current Topics in Visualization entry below instead, but this course may provide a useful perspective. For a taste, see scimaps.org, which this group implemented.

Current Topics in Data Science

Various Speakers, National Institute of Allergy and Infectious Diseases, U.S. National Institutes of Health "Data Science: Unlocking the Power of Big Data" (NIH Videocast, 2013: http://videocast.nih.gov/summary.asp?Live=12232&bhcp=1)

"The primary goal of the festival is to explore data science as it relates to scientific research. Experts will discuss how to extract useful and meaningful information from raw data as it relates to bioinformatics…"

Commentary. This can be considered a fairly high-level introduction to current topics in the field, in this case as they relate to biomedical data. The agenda for this "Bioinformatics Festival" is at https://respond.niaid.nih.gov/conferences/bioinformatics2013/Pages/Agenda.aspx.

Going Further. For the nitty-gritty on current algorithms in the field, see Current Topics in Big Data, below.

Current Topics in Big Data

Various Speakers, MMDS (Modern Massive Data Sets) Foundation "Workshop on Algorithms for Modern Massive Data Sets" (Stanford University, Summer 2012: http://stanford.edu/group/mmds/mmds2012.html)

> *"The goals of this series of workshops are to explore novel techniques for modeling and analyzing massive, high-dimensional, and nonlinearly-structured scientific and internet data sets; and to bring together computer scientists, statisticians, mathematicians, and data analysis practitioners to promote the cross-fertilization of ideas."*

Commentary. This biennial meeting has a very extensive set of talks on multiple aspects of big data, from the perspectives of databases, algorithms, linear algebra, statistics, machine learning, and numerous applications, including a tutorial by Rick Stevens of the Argonne National Laboratory and the University of Chicago on "The Biological, Algorithmic and Computational Challenges of Systems Biology." This, the fourth meeting in the series, was the first to be made available as video, but watch for the 2014 meeting at Berkeley.

Prerequisites. Big Data, Machine Learning, Linear Algebra, Statistics, Algorithms.

Current Topics in Machine Learning

Various Speakers, NIPS 2011 Conference Workshop "Big Learning: Algorithms, Systems, and Tools for Learning at Scale" (GoogleTechTalks, 2011: http://www.youtube.com/playlist?list=PL53DF7722DC0A0742)

> *"[The aim of the workshop was to] bring together parallel system builders in industry and academia, machine learning algorithms experts, and end users to identify the key challenges, opportunities, and myths of Big Learning."*

Commentary. The reach of Big Data is also extending into the machine learning community. There have been several Big Learning workshops now, associated with the annual NIPS (Neural Information Processing Systems) machine learning meetings. More workshop information is available at http://biglearn.org/2011, including an agenda so that viewers can better choose from among the numerous, rather technical talks. Several additional talks are available from another NIPS 2011 workshop on "Learning Semantics" (http://www.youtube.com/playlist?list=PLDAE29CD90F0512CC).

Current Topics in Visualization

Various Speakers "Fourth International Meeting on Visualizing Biological Data" (VIZBI, 2013: http://vizbi.org/Videos)

> *"Visualization is increasingly important in life science as data grows rapidly in volume and complexity. The VIZBI initiative is an international conference series bringing together researchers developing and using computational visualization to address a broad range of biological research areas; the conference also attracts participation from medical illustrators, graphic designers, and graphic artists. VIZBI is held annually in March, alternating between Europe and the USA… The conference featured 21 invited talks that reviewed the state-of-the-art and challenges in visualizing data from genomes, transcripts, proteins, cells, organisms, and populations."*

Commentary. This annual meeting is probably the best available introduction to the important topic of visualization in biology. Over 100 lectures are now available from past meetings; use the convenient filters on the page indicated to select different years or topics, among which are "Genome Data,"

"Transcript Data," "Proteins & Complexes," "Cellular Systems," "Organism Data," and "Populations & Evolution."

Data Science Seminars

(See seminars at http://www.youtube.com/playlist?list=PL9s5HIfOWP3Mfy0K-XK1fxf49NrWwk9M-)

Chemistry Department

Chemistry is now a full-fledged department (rather than being lumped under "Other") because of the importance of background in this field for biochemistry, structural biology, metabolomics, and especially drug discovery. A plethora of MOOCs have appeared in this arena, as can be seen below. In addition, the University of California, Irvine has initiated an exceptionally ambitious program called Open Chemistry (http://ocw.uci.edu/openchem) that is already well on the way to posting videos (though not MOOCs) of their entire four-year curriculum of courses under an open Creative Commons license.

Introduction to Chemistry

Dorian Canelas, Duke University "Introduction to Chemistry" (Coursera, Winter 2014: https://www.coursera.org/course/chem99)

> *"This is an introductory course for students with limited or no background in chemistry; chemical problem solving will be emphasized. The goal of the course is to prepare students for further study in the chemistry needed for many science, health, and policy professions. Topics include introductions to atoms, molecules, ions, the periodic table, stoichiometry, chemical reactions, bonding, thermochemistry, and gas laws."*

Commentary. Those with adequate high-school preparation in the subject will want to proceed directly to the next course, unless they are looking only for a quick refresher with no plans to go further in the topic.

Prerequisites. None.

General Chemistry

John Steven Hutchinson, Rice University "Chemistry: Concept Development and Application" Parts 1 and 2 (Coursera, Spring/Fall 2014: https://www.coursera.org/course/genchem1 and https://www.coursera.org/course/genchem2)

> [Part 1] *"The Atomic Molecular Theory… Atomic Masses and Molecular Formulae… The Structure of an Atom… The Electron Shell Model of an Atom… Quantum Electron Energy Levels in an Atom… Electron Orbitals and Electron Configurations in Atoms… Covalent Bonding and Electron Pair Sharing… Molecular Structures… Energy and Polarity of Covalent Chemical Bonds… Bonding in Metals and Metal-Non-Metals Salts… Molecular Geometry… Measuring Energy Changes in Chemical Reactions… Reaction Energy and Bond Energy"* [Part 2] *"Ideal Gas Law and the Kinetic Molecular Theory… Phase Transitions and Phase Equilibrium… Phase Equilibrium and Solutions… Reaction Rate Laws and Reaction Kinetics… Gas Reaction Equilibrium and Equilibrium Constants… Strengths of Acids… Buffers… Second Law of Thermodynamics… Free Energy and Thermodynamic Equilibrium."*

Commentary. This comprehensive, two-semester course uses a pedagogic method called the "Concept Development Approach" that emphasizes scientific reasoning from experimental observations as opposed to straightforward presentation of the concepts.

Prerequisites. Introduction to Chemistry or equivalent.

Textbook. A free online textbook is made available (http://cnx.org/content/col11444/latest). See also the free textbook associated with the Solid State Chemistry course below.

Alternatives. The University of California, Irvine has a rigorous sequence of non-MOOC video courses in its Open Chemistry program taught by Amanda Brindley (http://ocw.uci.edu/openchem/chem1a.html),

Donald Blake (http://ocw.uci.edu/openchem/chem1b.html), and Ramesh Arasasingham (http://ocw.uci.edu/openchem/chem1c.html). MIT's OpenCourseWare also has a non-MOOC "Principles of Chemical Science" taught by Catherine Drennan and Elizabeth Vogel Taylor, with excellent notes (http://ocw.mit.edu/courses/chemistry/5-111-principles-of-chemical-science-fall-2008).

Laboratory Chemistry

John Dolhun, Massachusetts Institute of Technology "Chemistry Laboratory Techniques" (MIT OpenCourseWare, Winter 2012: http://ocw.mit.edu/courses/chemistry/5-301-chemistry-laboratory-techniques-january-iap-2012)

> *"This course is an intensive introduction to the techniques of experimental chemistry and gives first year students an opportunity to learn and master the basic chemistry lab techniques for carrying out experiments."*

Commentary. A major shortcoming of online education is the lack of hands-on laboratory experiences, something that is discussed at length under the Laboratory Biology course in the Biology Department. In the case of chemistry, the MIT freshman "boot camp" in laboratory skills may be the closest available simulacrum. Students take this course by special admission in order to obtain a two-tiered qualification to work in chemistry research labs at MIT. Extensive notes are provided in addition to demonstration videos selected from an even larger library (http://ocw.mit.edu/resources/res-5-0001-digital-lab-techniques-manual-spring-2007/videos). However, the most intriguing resource is a recruiting video that MIT made recording the experiences of one recent class in the style of an MTV reality show, documenting their personal travails while interspersing reasonably cogent explanations of the experiments (http://ocw.mit.edu/high-school/chemistry/chemistry-lab-boot-camp). It succeeds in capturing the atmosphere of a teaching lab, if not of a research lab, and may help to bring the online student tantalizingly close to the actual hands-on experience.

Prerequisites. General Chemistry.

Introduction to Physical Chemistry 💻

Patrick O'Malley, Michael Anderson, and Jonathan Agger, Manchester University "Introduction to Physical Chemistry" (Coursera, Summer 2014: https://www.coursera.org/course/physicalchemistry)

> *"The course covers the key concepts of three of the principal topics in first-year undergraduate physical chemistry: thermodynamics, kinetics and quantum mechanics. These three topics cover whether or not reactions occur, how fast they go and what is actually going on at the sub-atomic scale."*

Commentary. This MOOC will go a bit deeper into the physical aspects of chemistry that, for various reasons, may form a useful part of the computational biologist's toolkit. It promises to offer a "virtual interactive practical" in each of the three major topics indicated above.

Prerequisites. General Chemistry, Calculus.

Textbook. None is required.

Thermodynamics 💻✓

Christopher Cramer, University of Minnesota "Statistical Molecular Thermodynamics" (Coursera, Winter 2014: https://www.coursera.org/course/thermodynamics)

> *"Quantized molecular energy levels and their use in the construction of molecular and ensemble partition functions is described. Thermodynamic state functions, their dependence on the partition function, and their relationships with one another (as dictated by the three Laws of Thermodynamics)*

are all examined in detail. Analysis and demonstration takes place primarily in the context of ideal and real gases."

Commentary. This well-crafted course offers more detail on those aspects of thermodynamics most likely to be of interest to computational types, *e.g.,* energy, phase changes, equilibria, partition functions, entropy, etc. There is also a lot of good practice at integral calculus within a theoretical context (as opposed to the usual analytic geometry exercises). The demonstrations are great fun, particularly the highly exothermic ones.

Evaluation.

Course Level	*Hours per Week*	*Course Grade*				*Student Grade*	*Curve*
		Lectures	*Homework*	*Assessment*	*Overall*		*Pass/"A"*
Intermediate	10	98	96	94	96	90.0	?/80

Prerequisites. Introduction to Physics, General Chemistry, Calculus.

Textbook. *Physical Chemistry: A Molecular Approach* by Donald McQuarrie and John Simon [60] is well-aligned with the MOOC, but not required. A more classical treatment of the subject can be found in a free online version of *Thermodynamics and Chemistry* by Howard DeVoe of the University of Maryland (http://www2.chem.umd.edu/thermobook).

Alternatives. Uday Gaitonde, Upendra Bhandarkar, and M.D. Atrey of the Indian Institute of Technology, Bombay, teach "Thermodynamics" on edX (https://www.edx.org/course/iitbombayx/iitbombayx-me209x-thermodynamics-1384). Among non-MOOC offerings, UC Irvine Open Chemistry has a statistical mechanics series with a course in "Thermodynamics and Chemical Dynamics" by Reginald Penner (http://ocw.uci.edu/openchem/chem131c.html) and MIT OpenCourseWare has "Thermodynamics and Kinetics" by Keith Nelson and Moungi Bawendi, which comes with a fine set of notes (http://ocw.mit.edu/courses/chemistry/5-60-thermodynamics-kinetics-spring-2008).

Personal Note. The author took the classroom version of the last-mentioned alternative course as an undergraduate, and while it was tough going, the methods and ideas conveyed have been echoed in so many other contexts and fields of study as to leave no doubt of the course's value. Consider the classic analogy between physical and informational entropy, as well as the widespread use of thermodynamic concepts in machine learning, *e.g.,* energy landscapes, ensemble methods, Boltzmann machines, and simulated annealing. Another recurring theme is the use of conceptual machines, *viz.,* Carnot's Theorem, which says that no engine can be more efficient than a Carnot engine, and the Church-Turing Thesis, which suggests that no digital computer can be more powerful than a Turing machine. Thermodynamics seems to consistently provide other fields with powerful and highly functional metaphors.

Solid State Chemistry

Michael Cima, Massachusetts Institute of Technology "Introduction to Solid State Chemistry" (edX, Fall 2013: https://www.edx.org/course/mitx/mitx-3-091x-introduction-solid-state-1751)

"[This] is a first-year course where chemical principles are explained by examination of the properties of materials. The electronic structure and chemical bonding of materials is related to applications and engineering systems throughout the course... The class will cover the relationship between electronic structure, chemical bonding, and atomic order, and characterization of atomic arrangements in crystalline and amorphous solids: metals, ceramics, semiconductors, and polymers (including proteins). There will be topical coverage of organic chemistry, solution chemistry, acid-base equilibria, electrochemistry, biochemistry, chemical kinetics, diffusion, and phase diagrams."

Commentary. This is an alternative introductory chemistry course especially well suited to engineers, given its focus on materials, but it also touches on biopolymers.

Textbook. The course uses a free online version of a textbook by Bruce Averill and Patricia Eldredge [61] (https://archive.org/details/fwk-archive-20121229-1790).

Personal Note. The author took the classroom version of this course from the late August Witt, a truly old-school Austrian chemist who had studied with Madame Curie and was a champion saber fencer. He taught this course with great flair to roughly half of all MIT undergraduates over more than two decades.

Alternatives. For the same target audience, "General Chemistry for Engineers" is a non-MOOC video course from Larry Brown of Texas A&M University (https://itunes.apple.com/us/itunes-u/chem-107-general-chemistry/id394433517).

Crystallography

Cora Lind, University of Toledo "Introduction to Crystallography" (Argonne National Laboratory, 2010: http://www.aps.anl.gov/Xray_Science_Division/Powder_Diffraction_Crystallography/Introduction_to_Crystallography or YouTube: https://www.youtube.com/playlist?list=PLBEB2F9103DBA52D1)

> *"Historical Introduction… Lattices, Planes, Reciprocal Lattice… Bragg's Law… Crystal Symmetry and Space Groups… Observing the Reciprocal Lattice… Intensity Data Collection… Data Reduction and Structure Factors… Superposition of Waves, more Structure Factors, Fourier Series… Phase Problem, Patterson Function, Direct Methods, Molecular Replacement… Structure Completion, Fourier Methods, Least Squares… Derived Results… Advanced Topics."*

Commentary. The Argonne National Lab, which hosts a great deal of protein crystallography at their advanced beamlines, has posted these non-MOOC lectures as an introduction to x-ray crystallography. However, while the methodologies described are used in protein structure determination, the instructor works on small molecules and this course does not cover protein structure determination proper. The videos are fuzzy but thankfully the slides are online; for the last few lectures only slides are available.

Textbook. None is used in the course above. *Biomolecular Crystallography: Principles, Practice, and Application to Structural Biology* by Bernhard Rupp [62] is well-reviewed, and will provide an appropriate biological slant. If the cost proves prohibitive, try its author's interactive web tutorial "Crystallography 101" (http://www.ruppweb.org/Xray/101index.html).

Alternatives. 2014 is the International Year of Crystallography, celebrating the founding of the field just over a hundred years ago by William and Lawrence Bragg. As part of the festivities the Royal Institute has erected a web site with videos and other resources, albeit at a very introductory level (http://richannel.org/collections/2013/crystallography). In default of a video course covering biomolecular crystallography, consider the web tutorial from the Department of Crystallography and Structural Biology in the Institute of Physical Chemistry "Rocasolano" (IQFR) of the Spanish National Research Council (http://www.xtal.iqfr.csic.es/Cristalografia/index-en.html).

Going Further. Those who want to pursue the mathematics behind crystallography (applied geometry and group theory) can get more than their fill in "Symmetry, Structure and Tensor Properties of Materials" by Bernhardt Wuensch of the Massachusetts Institute of Technology on MIT OpenCourseware (http://ocw.mit.edu/courses/materials-science-and-engineering/3-60-symmetry-structure-and-tensor-properties-of-materials-fall-2005). Another important mathematical aspect of crystallography is covered by Stanford's Brad Osgood in his "The Fourier Transform and Its Applications" (http://see.stanford.edu/see/courseinfo.aspx?coll=84d174c2-d74f-493d-92ae-c3f45c0ee091). Once again, however, none of these courses are MOOCs and there are few if any references to biomolecules.

Introductory Organic Chemistry

Jeffrey Moore, Michael Evans, and Nicholas Llewellyn, University of Illinois at Urbana-Champaign "Introductory Organic Chemistry" Parts 1 and 2 (Coursera, TBA: https://www.coursera.org/course/orgchem1a and https://www.coursera.org/course/orgchem1b)

> *"This course surveys structural chemistry of organic compounds with an emphasis on electronic structure, conformation and stereochemistry. Concepts and models are developed to build intuition about the stability and reactivity of organic compounds. These concepts will prepare students for a mechanistic-based approach to learning organic reactivity. Emphasis will also be placed on developing problem-solving skills unique to organic chemistry."*

Commentary. Computational biologists will benefit from a deeper understanding of the nature of the chemical bond and the molecular basis of biological systems. The ability to do some elementary bond-flipping will help tremendously with biochemistry and thus with pathway biology and metabolomics, while an understanding of organic stereochemistry, functional groups, hydrophobic interactions, etc. is the starting point in the study of structural biology and thus structural bioinformatics. Needless to say, the subject is *sine qua non* for working in drug discovery.

Prerequisites. General Chemistry.

Textbook. None is indicated. Of interest is the Virtual Textbook of Organic Chemistry at Michigan State University (http://www2.chemistry.msu.edu/faculty/reusch/VirtTxtJml/intro1.htm).

Alternatives. Since these courses appear to be offered infrequently by Coursera, the non-MOOC course listed in the previous catalog by Yale's Michael McBride can still be considered for asynchronous study (http://oyc.yale.edu/chemistry/chem-125a), as can the beginning course by James Nowick in the UC Irvine Open Chemistry series (http://ocw.uci.edu/openchem/chem51a.html).

Intermediate Organic Chemistry

Jeffrey Moore, Michael Evans, and Nicholas Llewellyn, University of Illinois at Urbana-Champaign "Intermediate Organic Chemistry" Parts 1 and 2 (Coursera, Spring 2013: https://www.coursera.org/course/orgchem2a and https://www.coursera.org/course/orgchem2b)

> *"This course covers intermediate topics on the structure and reactivity of organic compounds with an emphasis on electronic structure, pericyclic reactions and the chemistry of heteroaromatic compounds. Mechanistic concepts and models of reactivity are developed to build intuition about how organic compounds undergo their chemical transformations. An emphasis will be placed on developing problem-solving skills using frontier molecular orbitals to prepare students to think critically about the organic chemistry of living systems (e.g., nucleic acids, aromatic and heteroaromatic amino acids, etc.)."*

Commentary. Going the next step with organic chemistry may be overkill for most computational biologists but is included here for those interested in computational chemistry or drug discovery.

Personal Note. As an undergraduate, the author stopped after one course in organic chemistry, little dreaming that he would eventually end up in the pharmaceutical industry. In pharma R&D, chemists have something of an advantage on project teams in that it is easier for them to learn the necessary biology than it is for biologists to learn the necessary chemistry. Consider taking that second course in organic while you have momentum, if pharma or biotech might be in your future.

Prerequisites. Introductory Organic Chemistry.

Alternatives. As introduced above, there are follow-on organic chemistry courses from Yale (http://oyc.yale.edu/chemistry/chem-125b) and a pair of courses from UC Irvine Open Chemistry

(http://ocw.uci.edu/openchem/chem51b.html and http://ocw.uci.edu/openchem/chem51c.html) by David Van Vranen and James Nowick, respectively.

Biochemistry

(See listing in Biology Department.)

Chemical Biology

Motonari Uesugi, Kyoto University "The Chemistry of Life" (edX, Spring 2014: https://www.edx.org/course/kyotoux/kyotoux-001x-chemistry-life-858)

> *"The goal of this course is to develop skills for generating new ideas at the interface between chemistry and biology by analyzing pioneering studies."*

Commentary. Chemical biology should not be confused with biochemistry or biological chemistry, though they clearly overlap. Chemical biology is concerned with the use of chemistry to understand and manipulate biological systems, and is sometimes described as "chemistry-initiated biology." With no prerequisite for organic chemistry, this course is at the introductory end of the spectrum.

Prerequisites. Introduction to Chemistry, Introduction to Biology.

Textbook. None is required. *Chemical Biology*, edited by Deniz Ekinci, is an open access text published by Intech (http://www.intechopen.com/books/chemical-biology) that is rather more advanced and might be an appropriate follow-on.

Going Further. UC Irvine Open Chemistry has an upper-level "Introduction to Chemical Biology" by Gregory Weiss (http://ocw.uci.edu/openchem/chem128.html) that requires Organic Chemistry.

Medicinal Chemistry ✓

Erland Stevens, Davidson College "Medicinal Chemistry: The Molecular Basis of Drug Discovery" (edX, Spring 2014: https://www.edx.org/course/davidsonx/davidsonx-001x-medicinal-chemistry-1220)

> *"This course explores how to bring a drug from concept to market, and how a drug's chemical structure relates to its biological function. The course opens with an introduction to the drug approval process. This introduction combines the social, economic, and ethical aspects of drug discovery. Topics include how diseases are selected for treatment, the role of animal testing, and the costs of various discovery phases. The course then focuses on the scientific side of drug discovery. Topics include how drugs interact with biological molecules, drug absorption and elimination, and the discovery of weakly active molecules and their optimization into viable drugs."*

Commentary. This topic is the foundation of the process of drug discovery, from screening to a molecule that is safe and effective in humans. The course provides a good overview of all the factors bearing on drug action, pharmacokinetics, lead optimization, and more, and would be a good starting point for anyone with an eye for industry. The first part of the course should be straightforward for quantitative types, but the chemistry starts getting a little more serious at about the halfway point, starting with drug metabolism. Extensive use is made of JSdraw, made available for the course as a web platform, for depicting chemical structures.

Evaluation.

Course Level	*Hours per Week*	*Course Grade*				*Student Grade*	*Curve*
		Lectures	*Homework*	*Assessment*	*Overall*		*Pass/"A"*
Intermediate	6	94	90	92	93	103	70/—

Personal Note. Perhaps more than any other single course, this is the one the author wishes he had taken before going into the pharmaceutical industry. With the trend to compound screening in academia and an increasingly general concern there with going beyond tool compounds to practical therapeutics, study of this topic should be of interest to a much wider audience now than a decade ago.

Prerequisites. Introduction to Chemistry, Introduction to Biology, Introduction to Organic Chemistry.

Analytical Chemistry

Vicki Colvin, Rice University "Analytical Chemistry/Instrumental Analysis" (Coursera, Winter 2014: https://www.coursera.org/course/analyticalchem)

> *"Overview of instrumental analysis and basic chemistry review… Atomic spectroscopies and the analysis of metals… Calibration, QA/QC, and improving instrumental analysis… The basic principles of chromatography… Gas chromatography… Liquid chromatography… Vibrational spectroscopies… Electronic and optical sensors."*

Commentary. Why might a computational biologist take an interest in analytical chemistry? For one thing, because of its focus on instrumentation, which often provides the raw data for analysis in fields such as proteomics (even though this course primarily deals with inorganic analysis). Note that the instructor also teaches the course below on nanotechnology.

Prerequisites. General Chemistry.

Going Further. UC Irvine Open Chemistry has an upper-level course in "Organic Spectroscopy" taught by James Nowick (http://ocw.uci.edu/openchem/chem203.html).

Nanotechnology

Vicki Colvin, Rice University "Nanotechnology: The Basics" (Coursera, Fall 2013: https://www.coursera.org/course/nanotech)

> *"Nanotechnology is an emerging area that engages almost every technical discipline—from chemistry to computer science—in the study and application of extremely tiny materials. This short course allows any technically savvy person to go one layer beyond the surface of this broad topic to see the real substance behind the very small."*

Commentary. The field of nanotechnology continues to grow so quickly that it must soon overlap computational biology in some way or other. One likely intersection is in personalized and predictive medicine, especially in cancer, where a "bio-nano-info convergence" has been predicted [63]. This very brief overview of nanotechnology looks to be a worthwhile investment of time.

Going Further. Coursera also offers "Nanotechnology and Nanosensors" from Hossam Haick of the Technion (Israel Institute of Technology) (https://www.coursera.org/course/nanosensors), a much deeper dive into the field.

Prerequisites. Introduction to Chemistry, Physics.

Cheminformatics

David Wild and Abhik Seal, Indiana University "Introducing Cheminformatics" (Wikispaces: http://i571.wikispaces.com)

> *"The class covers basic techniques of cheminformatics, including representation of 2D and 3D chemical structures, chemical structure databases, chemical structures on the web and in the scholarly literature,*

as well as reviewing applications and algorithms such as cluster and diversity analysis, QSAR, predictive modeling, and data mining."

Commentary. There are no MOOCs covering cheminformatics, and in fact surprisingly few academic programs dedicated to the field. One of those few is run by Indiana University's David Wild, the co-editor of the *Journal of Cheminformatics* (http://www.jcheminf.com), who maintains a cheminformatics education portal (http://icep.wikispaces.com) that includes the material for this, his introductory course. The course is a Wiki-style outline with interspersed videos and numerous links to resources, and in default of a full-fledged MOOC it is the best available introduction a body of methods that are very important to drug discovery.

Prerequisites. Introduction to Chemistry, Introduction to Computer Science.

Computational Chemistry

Christopher Cramer, University of Minnesota "Computational Chemistry" (University of Minnesota, Spring 2014: http://pollux.chem.umn.edu/8021)

> *"Modern theoretical methods used in study of molecular structure, bonding, and reactivity. Concepts and practical applications. Semiempirical, ab initio, and density functional calculations of molecular electronic structure. Theoretical determination of molecular structure and spectra; relationship to experimental techniques. Molecular mechanics. Structure determination of large systems. Study of molecular properties and reactivity by theoretical methods. Computational tools for theoretical determination and lab for hands-on experience. Critical assessment of reliability of methods and theoretical work in literature."*

Commentary. This course covers the theoretical foundations of the methods for molecular modeling that are important in structural biology and drug discovery, though the course itself deals with the quantum and statistical mechanical underpinnings and does not much extend to biological topics. These lectures and class materials are being used at the University of Minnesota in a "flipped classroom" (i.e., one for which the students watch lectures online and then meet for interactive tutorial sessions) at the time of this writing. The instructor taught the Thermodynamics MOOC listed above, but he has no immediate plans to convert this course into a MOOC, though the videos and class materials will remain accessible online (personal communication).

Prerequisites. Both classical and quantum mechanics. The latter is touched on in the Introduction to Physical Chemistry listed above, though the "Quantum Principles" course expected soon from the University of California, Irvine (http://ocw.uci.edu/openchem/chem131a.html) will provide more thorough coverage of the topic, as does "Introductory Quantum Chemistry" by K.L. Sebastian of the Indian Institute of Science, Bangalore (http://nptel.ac.in/courses/104108057).

Alternatives. Gerbrand Ceder and Nicola Marzari of the Massachusetts Institute of Technology offer "Atomistic Computer Modeling of Materials" (http://ocw.mit.edu/courses/materials-science-and-engineering/3-320-atomistic-computer-modeling-of-materials-sma-5107-spring-2005), and a much briefer "Introduction to Molecular Dynamics" is available from Alejandro Strachan of Purdue University (http://nanohub.org/resources/5838#series). Neither of these are MOOCs, and unfortunately they don't cover biological applications, nor does the recommended course above. Some sense of such applications can be gotten from a pair of recent seminars from University of Chicago faculty: Gregory Voth's 2013 talk entitled "Molecular Modeling: A Window to the Biochemical World" (https://www.youtube.com/watch?v=w2-B54lbYjA) and Benoit Roux's 2012 talk on "Using Molecular Dynamics Simulations to Advance our Understanding of Complex Biological Systems" (https://www.youtube.com/watch?v=vN4RKPNOsEc).

Biology Department

The Biology Department has seen some much-needed additions to its curriculum, though they tend to be relatively narrow electives, such as Epigenetics and Apoptosis. Many other new courses that formerly would have been housed in this department have been placed in one of the new Departments, such as Neurosciences, Translational Sciences, and especially Computational Biology. There are still gaps in upper level courses in biology, notably in Structural Biology and Developmental Biology (though a stopgap is offered for the latter), and only non-MOOC courses are available for others. These deficiencies are partially offset by Seminars, including ones devoted to the important topics of Cell Signaling and Gene Regulation.

Introduction to Biology

Eric Lander, Massachusetts Institute of Technology "Introduction to Biology—The Secret of Life" (edX, Summer 2014: https://www.edx.org/course/mitx/mitx-7-00x-introduction-biology-secret-1768)

> *"As a student, you will first focus on the structure and function of macromolecules such as DNA, RNA and proteins. You will discover how changes in the structure of some of these macromolecules alters their functions and what the implications of such changes have on human health. As you continue in the course, you will apply an understanding of heredity and information flow within cells to human health and disease and will learn about molecular biological techniques and their potential to impact our changing world."*

Commentary. This, the edX version of the MIT introductory biology course recommended in the previous virtual catalog, is of similarly high quality and of course has the advantages of a MOOC. It is extensive in scope, with an estimated effort of 12 hours per week for 14 weeks. The course gets rave reviews from students (one calling it "life-changing"), especially for the online "experiments."

Personal Note. It was an early classroom version of this course that decided the author on majoring in biology, after having flirted with physics and several other fields. What made the biggest impression was discovering the extent to which biology could be quantitative and model-based, rather than simply descriptive. This realization was brought home in an introductory lecture by Salvador Luria on chemostats, when equations unexpectedly started appearing on the blackboard. Luria had been James Watson's PhD advisor and had just won his own Nobel Prize for work with the physicist Max Delbrück demonstrating Darwinian inheritance in bacteria using a sophisticated statistical model. While the knowledge conveyed in today's MOOC version of this course is unimaginably more advanced, it traces its lineage back to the new mindset that workers like Luria brought to biology. Take this MOOC and imagine how it will have evolved in a few more decades.

Prerequisites. High school biology and chemistry. Coursera offers a "Preparation for Introductory Biology: DNA to Organisms" by Adrienne Williams and Diane O'Dowd from the University of California, Irvine (https://www.coursera.org/course/introbiology), which is very brief indeed, and available for self-study by pressing the "Preview Lectures" button. See the previous catalog for other alternatives.

Textbook. The MIT site for the course indicates that any introductory biology textbook will serve. OpenStax has a free online text (http://cnx.org/contents/185cbf87-c72e-48f5-b51e-f14f21b5eabd), as does Annenberg Learner (http://www.learner.org/courses/biology/index.html). The latter resource, called *Rediscovering Biology*, is targeted to high-school biology teachers who would like to deepen their knowledge of the field, and comes with videos, case studies, and other useful tools, glossily packaged.

Alternatives. The MIT OpenCourseware version of this course is still a viable alternative (and perhaps even a preferred one for asynchronous self-study), as it has many other features of synchronous MOOCs

such as modularized structure with interactive quizzes, problem sets, exams, and additional helpful features (http://ocw.mit.edu/courses/biology/7-01sc-fundamentals-of-biology-fall-2011). While this course is excellent preparation for molecular biology with a genomic orientation, a more traditional syllabus is available in non-MOOC form from the University of California, Berkeley via their first year series (http://webcast.berkeley.edu/playlist#c,d,Biology,-XXv-cvA_iB5270cxdfbmEgITBs3bxxB and http://webcast.berkeley.edu/playlist#c,d,Biology,-XXv-cvA_iDb6YNfcoMsrhn9xzaGlPBE or http://www.youtube.com/view_play_list?p=-XXv-cvA_iB5270cxdfbmEgITBs3bxxB and http://www.youtube.com/view_play_list?p=-XXv-cvA_iDb6YNfcoMsrhn9xzaGlPBE). Finally, the Carnegie Mellon Open Learning Initiative has a non-video but interactive web course "Modern Biology" (http://oli.cmu.edu/courses/free-open/biology-course-details).

Going Further. There is a lot of biology that is not included in this rather specialized catalog, but which may yet be amenable to computational approaches. The closest thing to an ecology course is the Evolutionary Biology listing below, which has several lectures on that topic. A taste of botany can be had in Coursera's "What a Plant Knows (and Other Things You Didn't Know About Plants)" by Daniel Chamovitz and Aviva Katz of Tel Aviv University (https://www.coursera.org/course/plantknows), as well as in the Berkeley series mentioned above.

Laboratory Biology

Michael Meighan, University of California, Berkeley "General Biology Laboratory" (Berkeley Webcasts, Spring 2014: http://webcast.berkeley.edu/playlist#c,d,Biology,-XXv-cvA_iDULy3RrvlIJNhsTBHVdprw or http://www.youtube.com/view_play_list?p=-XXv-cvA_iDULy3RrvlIJNhsTBHVdprw)

> [Laboratory] *"General introduction to cell structure and function, molecular and organism genetics, animal development, form and function."*

Commentary. One of the major gaps in the MOOC movement is the lack of a means of providing students with hands-on laboratory experience, a hallmark of on-campus education. Unlike the case with computer science, meaningful online labs are hard to imagine with biology. Quite frankly, auditing this course is a poor substitute, but it at least offers some of the flavor of bench work. It is meant to accompany the general biology course at Berkeley, and consists of the preparatory and review lectures for the actual laboratory sessions, covering such topics as microbiological techniques, enzyme kinetics, PCR and sequencing, genetic complementation testing, and even a little bioinformatics. Online students may find it an exercise in frustration to simply watch this course, which is not even a MOOC, but no curriculum would be complete without at least a mention of "wet work."

Personal Note. The author spent a solid ten years before the bench, doing everything from fly genetics to tissue culture to recombinant DNA. How much value did this add to a subsequent career in "dry" computational biology? In all honesty, probably not ten years' worth, but some exposure to the process of experimentation and the mechanics of gathering biological data is useful in all sorts of ways. It facilitates communication with bench scientists, for instance on project teams in industry. It makes Materials and Methods sections of papers much more accessible, which can be important in data interpretation. It enhances understanding of sources of error and tradeoffs in experimental design. Most importantly, working with living organisms, isolated cells, or indeed any biological material helps one to develop a better sense of the complexities behind the data, and of the difficulties in acquiring it.

Prerequisites. Introduction to Biology.

Alternatives. A comprehensive "Microscopy Course" is available from iBiology, covering everything from the fundamentals to the most exotic technologies (http://www.ibiology.org/ibioeducation/taking-courses/ibiology-microscopy-course.html), though again it may be frustrating to viewers without access to a scope. The biotech supplier Bio-Rad Laboratories has a series of online tutorials that give close-up

views of the actual hands-on manipulations for a variety of lab procedures (http://www.bio-rad.com/en-us/education/support/tutorials), and they provide corresponding lab kits for educators. There are a number of interactive "virtual labs" available online, but most are rather cheesy animations at a very elementary level; the best of these is probably the award-winning set produced by the Howard Hughes Medical Institute (http://www.hhmi.org/biointeractive/virtual-lab-series), which has great visuals but is still completely scripted. The online Classical Genetics Simulator (http://www.cgslab.com) does at least allow for some latitude in experimental design, but it requires an instructor to set up a virtual class at some cost. In the final analysis, this might be the one area where spending some money on tuition for a live lab course, for instance at a local community college or extension campus, would be a worthwhile investment for online learners.

Going Further. In this arena, the ability for online students to go further will likely depend on future innovations on the part of online educators. Some ideas might be derived from the synthetic biology (http://biobuilder.org) and neuroscience (https://www.backyardbrains.com) communities, as well as from the pioneering approach to structural biology taken by David Baker with the FoldIt game platform (http://www.fold.it). Another model might be the approach to translational research advocated by Atul Butte of Stanford (see, e.g., http://videocast.nih.gov/summary.asp?live=10529), who points out that the wealth of experimental data already online can be the starting point for surprisingly low-cost hypothesis-driven research conducted by way of outsourcing, through web-based suppliers and contract research organizations (CROs). The commoditization of laboratory research suggests that there may be opportunities to take at least some aspects of laboratory education online in a cost-effective way.

Biochemistry

Kevin Ahern, Oregon State University "Biochemistry Free and Easy" (Oregon State: http://www.youtube.com/playlist?list=PLlnFrNM93wqwSmTn3kXZTdKeRXv-p6NOk)

> *"Introduction… Buffers… Amino Acids… Protein Structure… Protein Purification… Hemoglobin… Enzymes… Enzyme Mechanisms & Regulation… Allostery & Regulation… Blood Clotting… Carbohydrates… Signaling… Energy & Metabolic Control… Glycolysis… Gluconeogenesis… Glycogen Metabolism"*

Commentary. A full-fledged MOOC in this subject is still sadly lacking, but this video series is a noteworthy substitute, especially for the textbook, described below.

Prerequisites. Introduction to Chemistry, Introduction to Biology.

Textbook. Associated with the course is a free online textbook entitled *Biochemistry Free & Easy* (http://biochem.science.oregonstate.edu/biochemistry-free-and-easy) that is out of the ordinary in several respects. First, as a former professional science writer, the instructor's prose is highly accessible, and the book is densely illustrated and better laid out than most online-only texts. Second, the PDF contains links to the videos, allowing for close coordination; this is helpful, because the resolution of the slides in the videos is less than ideal. Third, the text is available for the iPad to be read in iBooks, with working links, if you can spare the several hundred megabytes of space required. Finally, the book is liberally sprinkled with boxes containing humorous song lyrics, limericks, and other doggerel related to biochemistry topics; depending on your nerd index, this could be either a bonus or a deal killer. An alternative is a free online version of the classic text by Lubert Stryer, available on PubMed for search only (http://www.ncbi.nlm.nih.gov/books/NBK21154), though it can be read continuously (if laboriously) by simply searching on successive chapter subheads in double quotes. See also the free resource from Carnegie Mellon described under Alternatives, below.

Personal Note. Perhaps more than any other artifact, biologists tend to have a special relationship with their first biochemistry textbook. It is one of the more challenging subjects in the curriculum, and the

information has a longer shelf life than many other fields of biology so that the textbook generally becomes a reference for years to come. The author learned from the first edition of Lehninger, an oddly elongated slab of a book in a brown canvas cover, that was one of the first of the lavishly illustrated scientific mega-textbooks that are now ubiquitous. "Lehninger" has since evolved to a sixth edition, bearing his name in the title only and no longer among the authors [64]. It costs well over $200, and many other textbooks for core disciplines are over $100, which will doubtless be off-putting to many online learners. That is why every effort has been made in this catalog to identify free resources, such as open access textbooks, course notes that are particularly polished, or PDFs of older editions that have been made available online. (However, since many textbook PDFs are easily found online that violate copyright restrictions, only those that are clearly sanctioned are listed here, generally with links from the academic home pages of the authors.) Nevertheless, for the most up-to-date and convenient versions of key textbooks, which today are often multimedia extravaganzas, even frugal online learners may want to spring for the real thing in judiciously chosen cases—and biochemistry may well be one of them.

Alternatives. If any aspects of the course above give one pause, a more conventional alternative is the (non-video) online self-study course from Carnegie Mellon University's Open Learning Initiative (http://oli.cmu.edu/courses/free-open/biochemistry-course-details). It has in-line assessments as well as extensive animations and Jmol-based activities. Also, "Biochemistry I" is taught by S. Dasgupta of the Indian Institute of Technology, Kharagpur (http://nptel.ac.in/video.php?subjectId=102105034), which is video-based but not a MOOC. See the previous catalog for other alternatives.

Genetics

Rosie Redfield, University of British Columbia "Useful Genetics" (Parts 1 and 2) (Coursera, Fall 2013/Winter 2014: https://www.coursera.org/course/usefulgenetics and https://www.coursera.org/course/usefulgenetics2)

> [Part 1] *"Introduction to DNA, genes and chromosomes and the relationships between human populations… The causes and immediate consequences of mutations… How mutations that change gene activity or function affect the properties of organisms. How mutations cause cancer… Sex determination and genes on sex chromosomes. How natural genetic variation is studied, and how it differs from classical alleles. Heritability and genome-wide association studies. Genetic variation for cancer risks… Kinds of DNA typing and genome analysis, and what can be learned from them about health risks, personal attributes and ancestry."* [Part 2] *"How genes and chromosomes are transmitted through the generations (including the molecular mechanisms of mitosis and meiosis)… Using genetic crosses as a research tool to investigate how genes work and what they do. Sex-linkage, pedigree analysis, and hypothesis testing… More about heritability and GWAS. Inbreeding in humans, crops and livestock, and evolution. Hybrids and genetically modified organisms… Polyploidy and aneuploidy, chromosome rearrangements, and genome evolution… The origin of life, mitochondrial genes and mutations, genetic mosaicism, fetal DNA in mothers, epigenetic inheritance, and other topics students may suggest."*

Commentary. The title "Useful Genetics" reflects a concern with teaching those aspects of the topic relevant to "understanding real-world issues, both personal and societal," but don't be fooled: this is a thorough introduction to genetics at the university level. The instructor is very personable, and her bright blue hair should tip you off that this is not your parents' genetics. Indeed, she has elsewhere described the curriculum design philosophy and made a case for revamping traditional approaches to the "canon" of genetics so as to better stimulate scientific thinking in students [65].

Textbook. The course uses several open-source textbooks, including *Open Genetics* by the University of Alberta's Michael Deyholos (https://era.library.ualberta.ca/public/view/item/uuid:405c04c1-1b18-

4943-bacb-5e128e890f03) and *OpenStax Biology*, a general biology text with detailed coverage of many topics in genetics (http://openstaxcollege.org/textbooks/biology).

Personal Note. The author was on the Genetics faculty of the University of Pennsylvania School of Medicine in the early days of the Human Genome Project, at the midway point between when he took undergraduate genetics and the present day. There were quantum leaps in the subject area content in each of the intervening periods, the first brought on largely by sequencing technology and the second by the completion of the human genome and further technological advances. Even so, the conceptual framework of genetics, developed long before Watson and Crick, has been resilient and remains one of the wonders of modern science. No field of biology is more closely allied to informatics, and it is useful for students to study the Mendelian foundations and understand how genetics was done at a time when a gene was more a notional object than a database entry. The principles of modeling and abstraction remain important even in the midst of a superabundance of sequence data—perhaps more so.

Prerequisites. Introduction to Biology.

Alternatives. The course Evolutionary Genetics in the Evolutionary Biology Department is a less comprehensive introduction to genetics that has a narrower focus on evolution. Udacity has a more rudimentary "Tales from the Genome: Introduction to Genetics for Beginners" taught in collaboration with 23andMe (https://www.udacity.com/course/bio110). See also the previous catalog for additional alternatives from Berkeley Webcasts.

Going Further. The Broad Institute has put up an excellent "Primer on Medical and Population Genetics" featuring eleven lectures by their senior staff (http://www.broadinstitute.org/scientific-community/science/programs/medical-and-population-genetics/primers/primer-medical-and-pop). Medical genetics is also well covered in the Genomic Medicine course in the Translational Sciences Department. Another important specialization is within the psychological context, as found in Coursera's "Introduction to Human Behavioral Genetics" by Matt McGue of the University of Minnesota (https://www.coursera.org/course/behavioralgenetics).

Molecular Biology

Thomas Alber, Qiang Zhou, and Qing Zhong, University of California, Berkeley "Molecular Biology: Macromolecular Synthesis and Cellular Function" (iTunes U, Fall 2009: http://itunes.apple.com/WebObjects/MZStore.woa/wa/viewPodcast?id=354820440)

> *"Molecular biology of prokaryotic and eukaryotic cells and their viruses. Mechanisms of DNA replication, transcription, translation. Structure of genes and chromosomes. Regulation of gene expression. Biochemical processes and principles in membrane structure and function, intracellular trafficking and subcellular compartmentation, cytoskeletal architecture, nucleocytoplasmic transport, signal transduction mechanisms, and cell cycle control."*

Commentary. This upper-level Berkeley course in their Biochemistry and Molecular Biology track, carried over from the previous catalog, is a thorough introduction to basic cellular information processing and as such is important background for bioinformatics. It is not a MOOC, and in fact only exists as an archive on iTunes U, but unfortunately Berkeley has not recorded video for the more recent offerings of this course, only audio. See the previous catalog for a detailed commentary on how to use the videos most effectively.

Prerequisites. Introduction to Biology. Biochemistry.

Textbook. *The Molecular Biology of the Cell*, by Bruce Alberts, Alexander Johnson, Julian Lewis, Martin Raff, Keith Roberts, and Peter Walter [66]. This old warhorse has been the standard for three decades, but the 5th edition from 2007 is probably about due for an update. Given that and the expense, one may

as well use the 4th edition from 2002, as suggested by the instructor, which is available as a free online text at PubMed (http://www.ncbi.nlm.nih.gov/books/NBK21054).

Going Further. The course "Eukaryotic Gene Expression" by P.N. Rangarajan of the Indian Institute of Science, Bangalore (http://nptel.ac.in/courses/104108056), is described in more detail in the previous catalog. Also see the Gene Regulation Seminars.

Cell Biology

Randy Schekman, Kunxin Luo and David Drubin, University of California, Berkeley "Cell Biology" (iTunes U, Spring 2009: http://itunes.apple.com/itunes-u/molecular-cell-biology-130/id354820424)

> *"This course is aimed at conveying an understanding of how cellular structure and function arise as a result of the properties of cellular macromolecules. An emphasis will be placed on the dynamic nature of cellular organization and will include a description of physical properties of cells (dimensions, concepts of free energy, diffusion, biophysical properties). Students will be introduced to quantitative aspects of cell biology and a view of cellular function that is based on integrating multiple pathways and modes of regulation (systems biology)."*

Commentary. Another upper-level Berkeley course carried over from the previous catalog, this one in their Cell and Developmental Biology track, offers a different take on the cell that is geared to current systems biology. Again, it is not a MOOC, and Berkeley has not produced any more recent video, but the course does now have the additional attraction of having been taught by a future Nobel laureate. See the previous catalog for additional notes.

Prerequisites. Introduction to Biology. Biochemistry.

Textbook. Same as Molecular Biology, above. Also, the *Nature* education site has a nice introductory e-book (http://www.nature.com/scitable/ebooks/cntNm-14749010).

Alternatives. There is now a "flipped classroom" course in this topic on the iBiology resource (http://www.ibiology.org/ibioeducation/taking-courses/cell-biology-flipped-course.html) that was used last year to teach seniors at the University of California, Davis. However, the ancillary class materials are only available to those who register as educators.

Developmental Biology

Various Speakers, GEM4 Summer School "Cellular and Molecular Mechanics with a Focus on Developmental Biology" (Massachusetts Institute of Technology, 2012: http://www.youtube.com/playlist?list=PLFn7fvIP7CbNOixWHdZ0AZrnVg2fDOhu5)

> *"Objectives [are to] educate researchers and graduate students about the fundamentals of cell and molecular biomechanics, and to provide an intense learning experience, and to facilitate interactions among engineers, biologists and clinicians. The goals are to help train a new generation of researchers with in-depth knowledge of mechanics and biology and to help engineers and biologists apply biomechanical approaches in biomolecular, cellular, tissue-level, animal model studies."*

Various Speakers "From Darwin to Evo-Devo" (Technion/Israel Institute of Technology, 2009: http://www.youtube.com/playlist?list=PL540284C7D74BC8F4)

> *"Marking the exact 150th anniversary of the publication of Charles Darwin's* On the Origin of the Species*, Technion hosted an international two-day scientific symposium in November 2009... Internationally renowned scientists who work at the interface of evolution and other life sciences presented their cutting-edge research in sessions that included Systems Biology; Evolutionary Ecology; Molecular Biology; and Evo-Devo."*

Commentary. Neither of the two entries above are MOOCs nor are they even standard courses in developmental biology, but in the absence of such a course they are offered as a stopgap because they cover two of the most active research areas in this field. The first involves the study of mechanical forces and motions at a cellular and tissue level, important in morphogenesis and physiology generally, while the second represents a synthesis of evolutionary and developmental biology ("evo-devo" for short). Only part of the first course is focused on developmental biology, and students could limit themselves to the lectures with "development" in their title. Most of the videos in the second are related to evo-devo either directly or indirectly, but once again they could be cherry-picked.

Textbook. These short courses have no assigned textbooks. The standard text in the field is by Scott Gilbert of Swarthmore College (a former grad school classmate of the author), which is available online at PubMed for search only (http://www.ncbi.nlm.nih.gov/books/NBK9983).

Personal Note. The author studied developmental biology for his PhD at Johns Hopkins, which had a storied history in the field, so the lack of an authentic MOOC in this area is a particular regret. The molecular biology of development was in its infancy then, and the courses above actually reflect the preoccupation at the time with comparative embryology and observable aspects of morphogenesis and organogenesis, now ushered into the 21st century by new interdisciplinary approaches. These more quantitative methods, together with the contributions of the genome and genomics, have made developmental biology amenable to computational analyses that were undreamt of then.

Alternatives. Students with no previous exposure at all to developmental biology may wish to first look at some basic lectures by Jason Pellettieri of Keene State College, who is developing an "Online Developmental Biology" course (https://www.youtube.com/channel/UCsBEiPyjWjev4OE4t-Y_7WQ). As of this writing only three lectures are available, but they nicely introduce the history of embryology as well as development in two important model organisms, *Drosophila* and *C. elegans*.

Going Further. See the Seminars in Developmental Biology for more molecular aspects of the field.

Evolutionary Biology

(See listing in Evolutionary Biology Department.)

Cancer Biology

Various Speakers, Center for Cancer Research, National Cancer Institute, U.S. National Institutes of Health "Translational Research in Clinical Oncology (TRACO)" (NIH Videocast, 2013: http://videocast.nih.gov/PastEvents.asp?c=53)

> *"TRACO is designed to provide an overview of general principles of cancer biology and treatment, epidemiology, mechanisms of resistance, metastasis, use of preclinical models, and identification of novel molecular targets."*

Commentary. The National Cancer Institute has been teaching this course in cancer biology to its postdoctoral and clinical fellows each year for over a decade. Though not a MOOC, it has the advantage of being absolutely current. The link above lists all past offerings in the series in reverse chronological order, so be sure to start with the first lecture in the current session and work forward by date. The course description can be found at http://ccr.cancer.gov/careers/courses/traco, where an agenda is given together with slide sets for most of the lectures.

Textbook. The course has no textbook, but the *Nature* education site has an e-book introducing the biology of cancer (http://www.nature.com/scitable/ebooks/cntNm-16550193)

Genomics 💻✓

John Hogenesch and John Isaac Murray, University of Pennsylvania "Experimental Genome Science" (Coursera, Fall 2013: https://www.coursera.org/course/genomescience)

> *"This course serves as an introduction to the main laboratory and theoretical aspects of genomics and is divided into themes: genomes, genetics, functional genomics, systems biology, single cell approaches, proteomics, and applications. We start with the basics, DNA sequencing and the genome project, then move to high throughput sequencing methods and applications. Next we introduce principles of genetics and then apply them in clinical genetics and other large-scale sequencing projects. In the functional genomics unit, we start with RNA expression dynamics, analysis of alternative splicing, epigenomics and ChIP-seq, and metagenomics. Model organisms and forward and reverse genetics screens are then discussed, along with quantitative trait locus (QTL) and eQTL analysis. After that, we introduce integrative and single cell genomics approaches and systems biology. Finally, we conclude by introducing… proteomic approaches."*

Commentary. This course is closely based on the long-established core course in the Penn Graduate Group in Genomics and Computational Biology, and in fact the material is used with Penn students in a "flipped classroom." Much of the value comes from close reading of papers assigned from the current literature, which are very well chosen; summaries and critiques of these are graded by peers, using complicated answer keys that often seem rather arbitrary.

Evaluation.

Course Level	*Hours per Week*	*Course Grade*				*Student Grade*	*Curve*
		Lectures	*Homework*	*Assessment*	*Overall*		*Pass/"A"*
Intermediate	8	90	85	90	88	76.2	45/—

Personal Note. The author confesses to some degree of bemusement about his mediocre grade in this MOOC, since he was co-Principal Investigator on the original National Science Foundation training grant that laid out the first bioinformatics curriculum for this program at Penn. Much of the assessment for this course was based on Coursera's system of peer review, whereby short essays or other submitted material are graded by 3-5 anonymous fellow students. The redundancy is designed to wash out the effects of any grading extremes, and peer review assessments sometimes also provide highly prescriptive grading rubrics (as did this course) to the same end. Many peer review items also allow for written feedback from the peer graders. Predictably, the system is occasionally plagued by "trolls" who post abusive or nonsensical comments as well as wildly outlying grades, and depending on the course these may or may not be rooted out by the teaching staff. Nevertheless, the system works surprisingly well overall, and especially when leeway is granted to peer graders the written feedback is generally conscientious and even illuminating.

Prerequisites. Molecular Biology.

Textbook. None is required. While the MOOC itself is heavily methods-oriented, a general text that is available free online at PubMed is *Genomes* by Terence Brown, though it is from 2002 and obviously dated (http://www.ncbi.nlm.nih.gov/books/NBK21128). The *Nature* education site has an excellent collection of up-to-date articles (http://www.nature.com/scitable/topic/genomics-19).

Alternatives. For what is, in effect, the computational version of this course, see Computational Genomics in the Computational Biology Department.

Proteomics

Various Speakers "Proteomics: Everything You Always Wanted to Know but Were Afraid to Ask" (Broad Institute, 2012: http://www.broadinstitute.org/partnerships/education/broade/proteomics-everything-you-always-wanted-know-were-afraid-ask)

> *"The focus of the workshop is on the most important technologies and experimental approaches used in modern mass spectrometry (MS)-based proteomics… 1) the essentials of the technology; 2) the most important classes of proteomics experiments and the specific sample requirements for each; 3) the software for proteomic data analysis and its proper use; 4) quantitative MS approaches applicable to global proteome and post-translational modification (PTM) analyses, including metabolic labeling methods, chemical labeling approaches and label-free methods; and 5) the basics of targeted, hypothesis-driven MS using multiple reaction monitoring MS."*

Commentary. This is not a MOOC, but a two-day workshop that is tutorial in nature and provides a compressed introduction to an important technology posing unique bioinformatics challenges. The five lectures listed above are followed by five additional ones focusing on applications and analysis, including statistical methods and annotation tools. A full set of slides is provided.

Textbook. While putatively focused on neuroscience, about half the chapters in the 2010 collection *Neuroproteomics* edited by Oscar Alzate are general background, and most importantly the text is freely available online at PubMed (http://www.ncbi.nlm.nih.gov/books/NBK56018).

Going Further. For a considerably more comprehensive treatment, Sanjeeva Srivastava of the Indian Institute of Technology, Bombay teaches "Proteomics: Principles and Techniques," which is not a MOOC but does have online quizzes and homework assignments (http://nptel.ac.in/courses/102101007). The instructor was associated for several years with the Proteomics course at the Cold Spring Harbor Laboratory.

Epigenetics

Marnie Blewitt, University of Melbourne "Epigenetic Control of Gene Expression" (Coursera, Summer 2014: https://www.coursera.org/course/epigenetics)

> *"This course will give an introduction to the fundamentals of epigenetic control. We will examine epigenetic phenomena that are manifestations of epigenetic control in several organisms, with a focus on mammals. We will examine the interplay between epigenetic control and the environment and finally the role of aberrant epigenetic control in disease."*

Commentary. The epigenetic code lends whole new levels of complexity to the study of gene regulation, and associated technologies have produced notable new sources of big data in biology that are only manageable and conceivably understandable by computational means. That makes this course important background for computational biologists, and the instructor does an admirable job of unweaving the complexities.

Evaluation.

Course Level	Hours per Week	Course Grade				Student Grade	Curve
		Lectures	Homework	Assessment	Overall		Pass/"A"
Intermediate	8	95	95	90	93	96.4	?/80

Prerequisites. Introduction to Biology, Genetics.

Textbook. None is required, but *Epigenetics* by Danny Reinberg and Thomas Jenuwein is recommended [67]. A new edition is due at the end of the year, at twice the price (the field is fast-moving). The *Nature*

education site has a nicely-done free e-book (http://www.nature.com/scitable/ebooks/chromatin-in-eukaryotic-regulation-16549786), while for a sampling of more advanced topics, InTech publishes a series of open access collections including *Epigenetics and Epigenomics* edited by Christopher Payne (http://www.intechopen.com/books/epigenetics-and-epigenomics), *Chromatin Remodelling* edited by Danuta Radzoch (http://www.intechopen.com/books/chromatin-remodelling), and *Methylation—From DNA, RNA and Histones to Diseases and Treatment* edited by Anica Dricu (http://www.intechopen.com/books/methylation-from-dna-rna-and-histones-to-diseases-and-treatment).

Immunology

Alma Moon Novothy, Rice University "Fundamentals of Immunology" Parts 1 and 2 (edX, Summer/Fall 2014: https://www.edx.org/course/ricex/ricex-bioc372-1x-fundamentals-immunology-1846 and https://www.edx.org/course/ricex/ricex-bioc372-2x-fundamentals-immunology-1574)

> *"This course will present the fundamentals of both innate and adaptive immunity, emphasizing functional interactions among cells and organs. We will cover signaling, pathogen recognition and the division of labor among myeloid, lymphoid and supporting cells."*

Commentary. This two-part course provides a biologist's-eye view of this complex topic, for those interested in gaining entry to the field of immunoinformatics. The presentation is unpretentious in the extreme, with the instructor often resorting to flimsy hand-made props, cartoons, and folksy analogies (*e.g.,* referring to the Fc region of a pathogen-bound antibody as a "kick me" sign). While this may not appeal to traditionalists, the down-to-earth approach probably will help the beginning student over the terminological barriers and other complexities of what is often an arcane subject.

Prerequisites. Introduction to Biology.

Textbook. None is required. PubMed has search-only online access to the 2001 edition of *Immunobiology* by Charles Janeway Jr., Paul Travers, Mark Walort, and Mark Shlomchik (http://www.ncbi.nlm.nih.gov/books/NBK10757).

Alternatives. As noted in the previous catalog, there are several non-MOOC video series available, including a shorter one presented from a medical perspective by Harris Goldstein of Albert Einstein Medical College (http://www.youtube.com/playlist?list=PL5703ABB5D07584D7) and another from a molecular and evolutionary standpoint by Gregory Beck of the University of Massachusetts (http://itunes.apple.com/us/itunes-u/intro-to-immunology-biol-378/id476313031). Also, Anjali Karande, Dipankar Nandi, and R. Manjunath of the Indian Institute of Science, Bangalore teach a non-MOOC "Essentials in Immunology" (http://nptel.ac.in/courses/104108055).

Going Further. Coursera has a course on "Vaccines" by Paul Offit of the University of Pennsylvania (https://www.coursera.org/course/vaccines).

Virology ✓

Vincent Racaniello, Columbia University "Virology I: How Viruses Work" (Coursera, Fall 2013/Winter 2014: https://www.coursera.org/course/virology)

> *"This introductory virology course emphasizes the common reactions that must be completed by all viruses for successful reproduction within a host cell and survival and spread within a host population. The molecular basis of alternative reproductive cycles are presented with examples drawn from a set of representative animal and human viruses, although selected bacterial viruses will be discussed."*

Commentary. The instructor co-authored a well-regarded virology textbook (see below), and his course was already popular on iTunes U long before he ported it to Coursera. He did a post-doc and made key discoveries with David Baltimore, as reflected in the very detailed molecular biology in this course. The slides are excellent, with illustrations drawn from the instructor's textbook, and the format is very effective by virtue of exploring the field by theme rather than reciting a virus-by-virus litany.

Evaluation.

Course Level	*Hours per Week*	*Course Grade*				*Student Grade*	*Curve*
		Lectures	*Homework*	*Assessment*	*Overall*		*Pass/"A"*
Intermediate	8	95	90	94	92	91.6	70/85

Textbook. *Principles of Virology* by S. Jane Flint, Lynn Enquist, the instructor, and Anna Marie Skalka [68].

Personal Note. The author did a post-doc studying the hepatitis B virus, just after the sequencing of its genome and the recognition of its kinship to retroviruses. Going through this MOOC brought home how much more is known now about the complex life cycle of this virus, and many others. What was most striking about revisiting the subject was the degree to which the molecular biology of the cell can be illuminated by studying the machinations of viruses, simply because they have evolved a profusion of distinctive offensive and defensive strategies that impinge on many different aspects of cell function, right down to the fine details of transcription and translation. Take this course for that reason alone.

Prerequisites. Molecular Biology.

Going Further. Part 2 of this course builds on the molecular biology to cover disease processes caused by viruses and host defense systems (https://www.coursera.org/course/virology2). A less technical overview of HIV is available in Coursera's "AIDS" by Kimberley Sessions Hagen of Emory University (https://www.coursera.org/course/aids).

Apoptosis ✓

Barbara Conradt, Ludwig-Maximilians-Universität München "Programmed Cell Death" (Coursera, Summer 2013: https://www.coursera.org/course/pcd)

> *"In this course, you will learn about the conserved, molecular machinery that eliminates cells through programmed cell death type I, also referred to as 'apoptosis'. Through apoptosis many cells are eliminated from our body during development and throughout adult life, and deregulated apoptosis can lead to various diseases such as cancer and auto-immune diseases… Introduction to programmed cell death and apoptosis… The BCL-2 family of proteins… Apaf proteins… Caspases… Mechanisms that regulate apoptosis… Non-apoptotic functions of components of the central apoptosis machinery… Diseases caused by deregulated apoptosis."*

Commentary. The instructor was personally involved in the elucidation of key genes and pathways in apoptosis, and her presentation of the material closely recapitulates the process of discovery throughout the field, describing the state of knowledge at each step of the way and what led to the next experiment. For this reason the course is doubly useful as a description of an important cellular phenomenon and as an account of scientific discovery over time from bench level. Especially important is the way it elucidates the critical role of model organisms in modern biology.

Evaluation.

Course Level	*Hours per Week*	*Course Grade*				*Student Grade*	*Curve*
		Lectures	*Homework*	*Assessment*	*Overall*		*Pass/"A"*
Intermediate	8	96	92	95	94	91.4	?/80

Prerequisites. Genetics, Molecular Biology.

Current Topics in Genomics

Various Speakers, National Human Genome Research Institute of the U.S. National Institutes of Health "The Genomics Landscape a Decade After the Human Genome Project" (GenomeTV, Spring 2013: http://www.youtube.com/playlist?list=PL1ay9ko4A8slWhqrCPupJ3GRPRp6bGiE8)

> *"The [symposium] looked at the accomplishments of the decade with an eye to what is on the horizon. The date of the symposium was significant, occurring in the month that the HGP was announced 10 years ago, and coinciding with the date 60 years ago when James Watson and Francis Crick's article describing DNA's double-helical structure was published. The symposium is timed with both historic achievements in mind."*

Commentary. This symposium features a day's worth of relatively short talks by prominent workers in genome studies including Sarah Tishkoff, David Kingsley, Claire Fraser, Jeff Botkin, David Williams, Kevin Davies, Nancy Cox, Ewan Birney, Levi Garraway, Dan Roden, David Botstein, and Francis Collins.

Current Topics in Immunology

Various Speakers "Immunology Interest Group" (Center for Cancer Research, National Cancer Institute, U.S. National Institutes of Health: https://ccrweb.cancer.gov/CCRLectures/iigoutside.aspx and click on "IIG Seminar Series")

> *"The Immunology Interest Group (IIG) organizes activities designed to promote information exchange and interactions among NIH scientists interested in the field of immunology, broadly defined."*

Commentary. Weekly seminars by visiting scientists to the NIH are archived by this interest group in a web conference format, going back to 2010. The recordings often have a lot of conference setup dead time that can be skipped over. Note that alternative links to this group's archives given on various NIH pages are flaky. As a fallback, the generally reliable NIH Videocast archive has more conventional videos of the same series (http://videocast.nih.gov/PastEvents.asp?c=28), but unfortunately stops at the end of 2011 .

Developmental Biology Seminars

(See seminars at http://www.youtube.com/playlist?list=PL9s5HIfOWP3Nzb82cs82Fo3xROot3Ab_-)

Cell Signaling Seminars

(See seminars at http://www.youtube.com/playlist?list=PL9s5HIfOWP3MvvVohCMZUpTvnjgQ20eeO)

Gene Regulation Seminars

(See seminars at http://www.youtube.com/playlist?list=PL9s5HIfOWP3O6RCxy5inkYF2oLzFaYNiQ)

Structural Biology Seminars

(See seminars at https://www.youtube.com/playlist?list=PL9s5HIfOWP3MwRhhdjt6dlH9wdu-KS-uh)

Computational Biology Department

This new department, as is the case at a number of real universities, now has the critical mass to stand on its own. In addition to computational courses this department is home to any primarily quantitative treatment of biology.

Bioinformatics Methods 💻✓

Nicholas James Provart, University of Toronto "Bioinformatic Methods I" and "Bioinformatic Methods II" (Coursera, Winter/Spring 2014: https://www.coursera.org/course/bioinfomethods1 and https://www.coursera.org/course/bioinfomethods2)

> *"This course focuses on employing existing bioinformatic resources—mainly web-based programs and databases—to access the wealth of data to answer questions relevant to the average biologist, and is highly hands-on… Topics covered include multiple sequence alignments, phylogenetics, gene expression data analysis, and protein interaction networks, in two separate parts… The first part, Bioinformatic Methods I, deals with databases, Blast, multiple sequence alignments, phylogenetics, selection analysis, RNA-seq, and metagenomics… The second part, Bioinformatic Methods II, covers motif searching, protein-protein interactions, structural bioinformatics, gene expression data analysis, and cis-element predictions."*

Commentary. This course is centered on "labs" involving the use of standard, usually web-based tools. It is designed for biologists but should also serve to familiarize computer scientists with the landscape of existing methods. This is almost purely a practical, hands-on course, conveying just enough information about the algorithms to support their intelligent use, in fairly cursory lectures. There is a heavy focus on plants, reflecting the instructor's background, but of course the methods are general. The assessments are largely cookbook, and have as much to do with navigating web resources as employing the tools, but that is much of what constitutes "end-user" bioinformatics today.

Evaluation.

(Part 1)

Course Level	*Hours per Week*	*Course Grade*				*Student Grade*	*Curve*
		Lectures	*Homework*	*Assessment*	*Overall*		*Pass/"A"*
Introductory	6	88	91	88	90	89.0	70/86

(Part 2)

Course Level	*Hours per Week*	*Course Grade*				*Student Grade*	*Curve*
		Lectures	*Homework*	*Assessment*	*Overall*		*Pass/"A"*
Intermediate	6	89	89	91	90	100.0	70/86

Prerequisites. Molecular Biology.

Textbook. None is required. See the Computational Biology course below for books that would provide deep background. One essential reference is *The NCBI Handbook*, provided online at PubMed, with good coverage of all the resources of the National Center for Biotechnology Information and related tools (http://www.ncbi.nlm.nih.gov/books/NBK143764).

Alternatives. Coursera's other offerings in this area include "生物信息学: 导论与方法" ("Bioinformatics: Introduction and Methods"), which is being taught in both Mandarin and English by Ge Gao and Liping Wei of Peking University (https://www.coursera.org/course/pkubioinfo), as well as "Введение в биоинформатику: биоинформатика в биологии и медицине" ("Introduction to Bioinformatics:

Bioinformatics in Biology and Medicine") in Russian from Anna Lapidus, Aleksey Makunin, and Pavel Dobrynin of Saint Petersburg State University (https://www.coursera.org/course/bioinfo). Another option is "Bioinformatics: Life Sciences on Your Computer" by Bob Lessick of Johns Hopkins University (https://www.coursera.org/course/bioinform). See the previous catalog for non-MOOC alternatives.

Going Further. The Computational Biology course and other entries immediately below cover many of the algorithms and their application at greater length.

Computational Biology

Doug Brutlag, Rhiju Das, Gavin Sherlock, Mike Snyder, and Peter Karp, Stanford University
"Computational Molecular Biology" (Stanford, Winter 2014: http://biochem218.stanford.edu)

> *"... a practical, hands-on approach to the field of computational molecular biology. The course is recommended for both molecular biologists and computer scientists desiring to understand the major issues concerning analysis of genomes, sequences and structures."*

Commentary. This carryover from the previous catalog is a wide-ranging bioinformatics practicum covering aspects of sequence analysis, genomics, phylogenetic reconstruction, gene regulation, and metabolic networks. It is not a MOOC, but there is an excellent set of slides in PDF format with numerous live links, which should be viewed in parallel with the video lectures, and a set of practical how-to videos as well. Where it overlaps with the Bioinformatics Methods course above, it provides much more detail about the workings of the algorithms and their various applications. Though it does have hands-on assignments, these are not as extensive as the previous course and much less effective outside the MOOC format.

Personal Note. The author taught a course with this title at the University of Pennsylvania in the years around 10 BGE (Before the Genome Era). In the meantime the syllabi of such courses have grown remarkably in both depth and breadth, such that they no longer provide comprehensive coverage of a fairly well-delimited subject area but must be considered overviews that introduce a wide range of alternative specializations, as evidenced by this catalog. Stated another way, what had once been itself a specialization may now be thought of as a full-fledged scientific discipline, albeit a quintessentially interdisciplinary one.

Prerequisites. Molecular Biology.

Textbook. The instructor recommends any of a number of books, which can be found in the slides near the beginning of the first lecture (http://biochem218.stanford.edu/01Genomics&Bioinformatics.pdf). One not mentioned, but a favorite of this author, is *Understanding Bioinformatics* by Marketa Zvelebil and Jeremy Baum [69]. Another, and perhaps the most recently updated, is *Introduction to Bioinformatics* by Arthur Lesk [70].

Alternatives. See the previous catalog for alternatives.

Going Further. The Sequence Analysis Algorithms and Genomics Algorithms courses below cover the computer science behind the tools in depth and involve actual implementation.

Computational Genomics

Rafael Irizarry and Michael Love, Dana Farber Cancer Institute and Harvard School of Public Health
"Data Analysis for Genomics" (edX, Spring 2014: https://www.edx.org/course/harvardx/harvardx-ph525x-data-analysis-genomics-1401)

> *"The purpose of this course is to enable students to analyze and interpret data generated by modern genomics technology, specifically microarray data and next generation sequencing data. We will focus*

on applications common in public health and biomedical research: measuring gene expression differences between populations, associated genomic variants to disease, measuring epigenetic marks such as DNA methylation, and transcription factor binding sites… The course covers the necessary statistical concepts needed to properly design experiments and analyze the high dimensional data produced by these technologies. These include estimation, hypothesis testing, multiple comparison corrections, modeling, linear models, principle component analysis, clustering, nonparametric and Bayesian techniques. Along the way, students will learn to analyze data using the R programming language and several packages from the Bioconductor project."

Commentary. This is the biological version of big data and data analytics, and as such it is central to modern bioinformatics. This welcome addition to the curriculum focuses closely on practical aspects of large-scale genomic data analysis, though it does not attempt to cover genome assembly, sequence alignment, and wider topics in systems biology or comparative genomics. The primary lecturer is one of the founders of the R-based Bioconductor project, an open source and open development software platform for the analysis of genomic data (http://bioconductor.org).

Prerequisites. Molecular Biology, Statistics and programming skills (including R).

Textbook. Only standard R books are recommended; for these, see Statistical Programming in the Data Science Department. For Bioconductor, the manual by Thomas Girke of the University of California, Riverside, is recommended (http://manuals.bioinformatics.ucr.edu/home/R_BioCondManual).

Alternatives. The "EMBO Practical Course on Analysis of High-Throughput Sequence Data" (http://www.ebi.ac.uk/training/online/course/embo-practical-course-analysis-high-throughput-seq), mentioned in the previous catalog, is not a MOOC but closely coordinates video lectures with detailed analysis exercises, with tutorial handouts and code supplied, also using R and Bioconductor. Topics include short read analysis, ChIP-Seq data and analysis, statistical concepts, differential expression by RNA-Seq, and allele-specific expression and eQTL. The Broad Institute's GATK software package (http://www.broadinstitute.org/gatk/index.php), which supports analysis of next-generation resequencing projects, has comprehensive workshops associated with it, most recently in 2013 (http://www.youtube.com/playlist?list=PLlMMtlgw6qNjSDAnDIDurM5g_C73hK4ya).

Going Further. Naomi Altman, Harmen Bussemaker, Sean Davis, and Olivier Elemento taught the Cold Spring Harbor Laboratory (CSHL) course in "Statistical Methods for Functional Genomics" in 2013 (https://meetings.cshl.edu/courses/2013/c-data13.shtml), the lectures for which are available on YouTube (https://www.youtube.com/playlist?list=PLCMsYfrNgAZMU3IdLpXZu85w4L_FwQn86). It is more extensive than the recommended course above (with a total of 32 hours of videos), covers more examples, and includes a number of research talks, so is appropriate as a follow-up even though it overlaps and is not a MOOC.

Personal Note. While a post-doc, the author took two of these legendary CSHL courses over successive summers, in Molecular Cloning and Advanced Molecular Cloning. These rigorous 1-3 week courses at the historic and picturesque CSHL campus were known for marathon sessions in the lab, often running well into the night, and are still among the most intense learning experiences available to biologists-in-training. Unfortunately, it is also the kind of experience that online learners will find difficult to come by. (In theory, CSHL accepts applications from anyone, and even offers financial support in some cases, but only a very limited number of spots are available.) Computational biologists can at least console themselves that they are less disadvantaged by a lack of in-person laboratory experiences than "wet" biologists. See the course Laboratory Biology in the Biology Department for a discussion of what may be necessary to bring such laboratory experience to the MOOC universe.

Computational Evolution 💻✓

Anders Gorm Pederson, Technical University of Denmark "Computational Molecular Evolution" (Coursera, Winter 2014: https://www.coursera.org/course/molevol)

"The main goal of this course is to give an introduction to theory and algorithms in the field of computational molecular evolution. We will cover basic evolutionary theory (common descent, natural selection, genetic drift, models of growth and selection), and the main types of algorithms used for constructing and analyzing phylogenetic trees (parsimony, distance based methods, maximum likelihood methods, and Bayesian inference). We will also discuss the role of statistical modeling in science more generally."

Commentary. This nicely constructed course provides a virtual disk image for Oracle VirtualBox with pre-installed Linux and software including PAUP, PAML, MrBayes and other tools that would otherwise be tedious and/or expensive for the student to bring together. While the challenges are mainly to do with parameter-setting (which is all-important with these tools), the arrangement works smoothly to give the user a feel for the workflows, and the lectures are very effective.

Evaluation.

Course Level	*Hours per Week*	*Course Grade*				*Student Grade*	*Curve*
		Lectures	*Homework*	*Assessment*	*Overall*		*Pass/"A"*
Intermediate	6	96	94	91	94	99.4	70/90

Prerequisites: Introduction to Biology. Computational Molecular Biology, some math, and knowledge of UNIX are helpful.

Textbook. *Inferring Phylogenies* by the legendary Joseph Felsenstein [71] is not essential, but will greatly enhance one's experience of the course.

Computational Ecology

Tanya Berger-Wolf, Jason Leigh, Daniel Rubenstein, and Iain Couzin, University of Illinois at Chicago and Princeton University "Field Course in Computational Ecology" (University of Illinois at Chicago, Fall 2011: http://www.cs.uic.edu/bin/view/ComputationalEcology or YouTube: https://www.youtube.com/channel/UCgUpof3XPs-EjkCQG55dj1g)

"A unique highly integrated field course is offered in Kenya (at the Mpala Research Centre) where US biology (PhD from Princeton University) and engineering students (PhD from UIC) will work with faculty in both disciplines to learn how to ask questions, frame hypotheses and understand how and why the disciplines and cultures do this differently."

Commentary. This course was put together as background preparation for students embarking on field work in Kenya. While it includes lectures in general topics like network analysis and machine learning, there are also more directed talks on population biology and ecology, behavioral ecology, and collective behavior. Online auditors can only envy (or not) the students going on from this to do projects in the wild, but either way this course provides an interesting insight into interdisciplinary studies in this field.

Going Further. See the Mathematical Ecology course below for coverage of population biology in much greater depth.

Computational Neuroscience 💻✓

(See listing in Neurosciences Department.)

Sequence Analysis Algorithms

Pavel Pevzner, Philip Compeau, and Nikolay Vyahhi, University of California, San Diego "Bioinformatics Algorithms (Part 1)" (Coursera, Fall 2013: https://www.coursera.org/course/bioinformatics)

> *"In this course, we will take a look at some of the algorithmic ideas that are fundamental to an understanding of modern biology. Computational concepts like dynamic programming and graph theory will help us explore algorithms applied to a wide range of biological topics, from finding regulatory motifs to reconstructing the tree of life. Throughout the process, we will apply real bioinformatics algorithms to real genetic data."*

Commentary. This course is an in-depth exploration of a wide range of algorithms important for modern sequence analysis. It makes use of a custom-built educational platform called Stepic, essentially an e-book that is closely integrated with graded programming challenges implemented in the language of your choice. The grading in Stepic is in turn integrated with the Coursera platform, and while there is occasional downtime the arrangement works fairly well overall. The main disadvantage of Stepic is that it is complete and self-contained, such that the student is given little incentive to watch the separate videos of lectures by Pavel Pevzner, which are terrific.

Evaluation.

Course Level	*Hours per Week*	*Course Grade*				*Student Grade*	*Curve*
		Lectures	*Homework*	*Assessment*	*Overall*		*Pass/"A"*
Intermediate	12	96	96	93	94	90.6	60/80

Prerequisites. Introduction to Biology and programming experience.

Textbook. As noted above, Stepic is the main resource, and is available asynchronously for self-study (https://stepic.org). *An Introduction to Bioinformatics Algorithms* by the instructor and Neal Jones [72] is an excellent book, but is now dated.

Alternatives. The non-MOOC course recommended in the previous catalog, by Steve Skiena of Stony Brook University, remains an option (https://www.youtube.com/playlist?list=PLA48145CC64FE7990).

Genomics Algorithms

Pavel Pevzner, Philip Compeau, and Nikolay Vyahhi, University of California, San Diego "Bioinformatics Algorithms (Part 2)" (Coursera: TBA)

> *"... gene expression analysis, evolutionary tree reconstruction, computational aspects of human genetics, computational proteomics, genome annotation, network biology, and many other topics."*

Commentary. This is the continuation of the Sequence Analysis Algorithms course, dealing with a whole new set of data structures and algorithmic challenges. As with the first part, it is important to note that this course covers the design and implementation of the algorithms, rather than their practical use.

Prerequisites. Introduction to Biology and programming experience.

Textbook. See the Sequence Analysis Algorithms course above.

Quantitative Biology

Ron Milo, Weizmann Institute of Science "Cell Biology by the Numbers" (Weizmann Institute, Fall 2013: http://www.weizmann.ac.il/plants/Milo/CBN)

> *"Cell biology becomes increasingly richer* [sic] *in data and quantitation. In the course we will explore how knowledge of key numbers can be used to make useful inferences in molecular cell biology. We will*

experience the practice of making back of the envelope calculations (so called Fermi problems) in biology. We will learn how to avoid pitfalls in interpretation and correctly balance the complexity of biology and the clear-cut deductions often used potently in the physical sciences."

Commentary. This short non-MOOC course is an exploration in depth of a skill that most scientists develop in an *ad hoc* fashion, the "back of the envelope" calculation, applied to such topics as quantities and relative sizes of molecules and cellular assemblages, rates and durations of biological processes, energy levels, error rates, etc. It presents no mathematical difficulties to speak of but helps the student to develop a better "feel" for the cell, with occasional surprising insights.

Prerequisites. Introduction to Biology.

Alternatives. A more conventional approach to quantitative biology is available from edX in the "Quantitative Biology Workshop" by various faculty and staff of the Massachusetts Institute of Technology (https://www.edx.org/course/mitx/mitx-7-qbwx-quantitative-biology-1714). It provides exposure at an introductory level to data analysis tools and methods.

Going Further. Though not directed at biology, the edX course "Street-Fighting Math" by Sanjoy Mahajan of the Massachusetts Institute of Technology, takes estimation to a new level (https://www.edx.org/course/mitx/mitx-6-sfmx-street-fighting-math-1501). It teaches heuristic reasoning techniques including "dimensional analysis, easy cases, lumping, pictorial reasoning, taking out the big part, and analogy" applied in such arenas as "mental calculation, estimating population growth rates, understanding drag without differential equations, singing musical intervals to estimate logarithms, approximating integrals, summing infinite series, and turning differential equations into algebra." Note, however, that while the course is in a MOOC framework, it is more like an online textbook with only occasional short videos.

Mathematical Biology

Germán Enciso, University of California, Irvine "Mathematical Biology" (UCIrvineOCW, Winter 2014: https://www.youtube.com/playlist?list=PLqOZ6FD_RQ7lnGZ7fkn503y_7U4rrJ-Se)

"This course… consists of an introduction to modeling biological problems using continuous ODE methods… We describe the basic qualitative behavior of dynamical systems in the context of a simple population model. As time allows, we will then discuss other types of models such as chemical reactions inside the cell, or excitable systems leading to oscillations and neuronal signals."

Commentary. This non-MOOC course begins *in medias res*, since it is normally preceded at Irvine by a course dealing with discrete modeling that is not online, but it would appear that this material can stand alone. In any case ordinary differential equations (ODEs) are historically more significant in mathematical biology, for instance in enzyme kinetics and population models. Since it emphasizes graphical analysis and qualitative solutions, the course does not require Differential Equations as a prerequisite and introduces the necessary Linear Algebra, but having that preparation is likely to enrich the student's experience. The lectures are done on a whiteboard and are perfectly legible. The syllabus is available for inspection at (https://eee.uci.edu/14w/45015/home/Math+113B+Syllabus.pdf).

Prerequisites. Calculus. Differential Equations and Linear Algebra helpful.

Textbook. Detailed course notes by Frederic Wan are provided as the main reading material (http://www.math.uci.edu/~mcbu/courses/math113b/lecture/113b_notes.pdf). For additional study, Eduardo Sontag of Rutgers University has a beautiful and extensive set of notes on the subject (http://www.math.rutgers.edu/~sontag/FTP_DIR/notes_biomath.pdf), recently updated to include coverage of systems biology.

Alternatives. The more wide-ranging "Biomathematics" by Ranjith Padinhateeri of the Indian Institute of Technology, Bombay (http://nptel.ac.in/syllabus/syllabus.php?subjectId=102101003) is actually a rapid review of university level math, but it covers a wealth of biological applications such as diffusion, polymerization, crystallography, and the statistical thermodynamics of macromolecules. Also worth a look is "A Mathematical Way to Think About Biology" by David Liao, a recent PhD in Physics from Princeton, whose course on Udemy (https://www.udemy.com/lookatphysics) refers to his own very polished online resource (http://qbio.lookatphysics.com). It also reviews college math and provides many physics-oriented examples of applications to biology, especially systems biology.

Going Further. The various modeling courses in the Systems Biology Department give a greater variety of perspectives on mathematical modeling and greater depth as regards biological applications.

Mathematical Ecology

Wayne Getz, University of California, Berkeley "Modeling and Management of Biological Resources" (Berkeley Webcasts, Fall 2013: http://webcast.berkeley.edu/playlist#c,s,Fall_2013,-XXv-cvA_iB_zUNkmb-ArKeV4TVLokuR or http://www.youtube.com/view_play_list?p=-XXv-cvA_iB_zUNkmb-ArKeV4TVLokuR)

> *"Models of population growth, chaos, life tables, and Leslie matrix theory. Harvesting and exploitation theory. Methods for analyzing population interactions, predation, competition. Fisheries, forest stands, and insect pest management. Genetic aspects of population management. Mathematical theory based on simple difference and ordinary differential equations."*

Commentary. This course covers methods in mathematical modeling of population growth from the ground up, and so overlaps with the previous course and may be considered an alternative to it. This one, however, goes further to describe a variety of applications in ecology rather than topics like enzyme kinetics. It is not a MOOC and is less than ideal for that reason, but online students can access its primary teaching tool, a modeling package called Nova that features an easy-to-use graphical interface (http://www.novamodeler.com).

Prerequisites. Calculus.

Textbook. See the Mathematical Biology course above for books covering the necessary math.

Current Topics in Bioinformatics Methods

Various Speakers (Canadian Bioinformatics Workshops, 2013: http://bioinformatics.ca/workshops/2013)

> *"The Canadian Bioinformatics Workshops offer hands-on, intensive workshops to gain bioinformatics skills training in various topic areas relevant to life science research today."*

Commentary. This venerable series of workshops was founded in 1999 and since 2009 have been made available as online videos under a Creative Commons License. Despite the fact that the hands-on segments of the multi-day workshops are not available, the preparatory lectures are comprehensive and highly practical in describing the tools and offering advice in their use. The recurring topics of the workshops include "Exploratory Analysis of Biological Data using R," "Bioinformatics for Cancer Genomics," "Informatics for RNA-sequence Analysis," "Informatics on High Throughput Sequencing Data," "Pathway and Network Analysis of -omics Data," "Flow Cytometry Data Analysis using R," "Microarray Data Analysis," and "Informatics and Statistics for Metabolomics." (On the rare occasions when a workshop has flawed video, look in prior years for another edition.)

Current Topics in Computational Biology

Various Speakers, "CBIIT (Center for Biomedical Informatics and Information Technology) Speaker Series" (National Cancer Institute, U.S. National Institutes of Health: https://wiki.nci.nih.gov/display/CBIITSpeakers/CBIIT+Speaker+Series+Presentations+and+Resources)

> *"The CBIIT Speaker Series is a bi-weekly knowledge-sharing forum featuring internal and external speakers discussing topics of interest to the biomedical-informatics and cancer-research communities."*

Commentary. Though slanted toward cancer biology, the several dozen talks accumulated so far in this series run the gamut of computational biology concerns. The collection can also be viewed on YouTube (http://www.youtube.com/playlist?list=PLYKy4VbxNln5j89ESpYBVUkeFDbmQwxYG).

Current Topics in Computational Genomics

Various Speakers, "Current Topics in Genome Analysis" (National Human Genome Research Institute, 2014: http://www.genome.gov/12514288)

> *"A lecture series covering contemporary areas in genomics and bioinformatics."*

Commentary. This series of 13 extended guest lectures in course format is offered every other year by the National Human Genome Research Institute of the U.S. National Institutes of Health. Topics include "The Genomic Landscape circa 2014," "Biological Sequence Analysis I & II," "Genome-Scale Sequence Analysis," "Regulatory and Epigenetic Landscapes of Mammalian Genomes," "Applications of Genomics to Improve Public Health," "Introduction to Population Genetics," "Genomic Approaches to the Study of Complex Genetic Diseases'" "Identifying the Genomic Basis of Rare Diseases," "Phamacogenomics," "Large-Scale Expression Analysis," "Genomic Medicine," "Next-Generation Sequencing Technologies," and "Genomics of Microbes and Microbiomes." Handouts are provided.

Going Further. See the previous catalog for additional tutorials.

Prerequisites. Molecular Biology.

Computational Biology Seminars

(See seminars at http://www.youtube.com/playlist?list=PL9s5HIfOWP3Nd-6vEmAogm_gHdGky2ZOL)

Evolutionary Biology Department

This is another area that has seen a profusion of new courses, and is distinctive enough to create a new department. It encompasses not only molecular evolutionary biology but also paleontology extending to human origins, which is increasingly making use of quantitative approaches, and even astrobiology, which includes both hypotheses of extraterrestrial life and origins of life on earth.

Introduction to Evolution

Joel Cracraft and David Randle, American Museum of Natural History "Evolution: A Course for Educators" (Coursera, Fall 2013: https://class.coursera.org/amnhevolution)

> *"… the course explores the history of evolutionary theory and the evidence that supports it. We will learn about patterns of human evolution and societal implications of modern evolutionary biology, and how scientists determine relatedness among living and extinct organisms."*

Commentary. The course is designed for teachers and in four weeks provides only a taste of the main topics surrounding evolution, but it serves as a quick introduction for utter novices and pays appropriate attention to Darwin. Assessment includes a short peer-reviewed essay, which is common for MOOCs in the humanities but less so in the sciences.

Darwinism

William Durham et al., Stanford University "Darwin's Legacy" (Stanford Continuing Studies Program, Fall 2008: http://www.youtube.com/playlist?list=PLF2E17B4CDCCE15F5)

> *"Charles Darwin launched a sweeping new theory of life in his epic book,* On the Origin of Species *(1859). The theory opened eyes and minds around the world to a radical new understanding of the flora and fauna of the planet. Here, Darwin showed for the first time that no supernatural processes are necessary to explain the profusion of living beings on earth, that all organisms past and present are related in a historical branching pattern of descent, and that human beings fall into place quite naturally in the web of all life… [In this course] we will meet weekly with leading Darwin scholars from around the country to learn about Darwin's far-reaching legacy in fields as diverse as anthropology, religion, medicine, psychology, philosophy, literature, and biology."*

Commentary. This non-MOOC course could perhaps be more accurately titled the "History of Evolutionary Thought," and there is no better way to approach this than to study Darwin himself. Darwin's bicentenary is beginning to fade into memory, but it was the occasion for a number of courses and seminars that are still of value for historical, philosophical, and scientific perspectives on the theory of evolution. This was one of the best.

Textbook. One can't do much better than to go straight to the source (http://darwin-online.org.uk).

Personal Note. Darwin's home in southeast England, Down House, has been made into a museum (http://www.english-heritage.org.uk/daysout/properties/home-of-charles-darwin-down-house). Visiting there is a transformative experience and highly recommended. Besides the beautiful grounds where Darwin did experiments, highlights include the surprisingly modest study where he wrote *On the Origin of Species*, and a rather harrowing replica of his cabin on the Beagle.

Alternatives. Cambridge University laid on an extremely well-produced week-long collection of talks including the likes of Richard Dawkins, Paul Nurse, John Sulston, Harold Varmus, Chris Ponting, and many other luminaries, interspersed with the actor Terry Molloy reading excerpts from Darwin's

writings (http://www.youtube.com/playlist?list=PL34FBAB97197AB401). The U.S. National Institutes of Health also hosted a Darwin event featuring Donald Johanson, David Kingsley, David Haussler, Elaine Ostrander, Ajit Varki, and Pardis Sabeti (http://videocast.nih.gov/summary.asp?Live=7493).

Evolutionary Biology

Stephen Stearns, Yale University "Principles of Evolution, Ecology and Behavior" (Open Yale, Spring 2009: http://oyc.yale.edu/ecology-and-evolutionary-biology/eeb-122)

> *"This course presents the principles of evolution, ecology, and behavior for students beginning their study of biology and of the environment… Recent advances have energized these fields with results that have implications well beyond their boundaries: ideas, mechanisms, and processes that should form part of the toolkit of all biologists and educated citizens."*

Commentary. Although not a full MOOC, this course is carried over from the previous catalog because of its very thorough treatment of evolutionary biology in an ecological context. Especially interesting is the coverage of evolutionary medicine, in which the instructor is a leading light.

Textbook. *Evolution: An Introduction* by the instructor and Rolf Hoekstra [73]. For ecology, the course mentions several texts, but the Wikibooks entry is very good (http://en.wikibooks.org/wiki/Ecology).

Personal Note. The author's first experience as a Teaching Assistant (TA) was for the version of this course taught at Johns Hopkins. Being a TA is a very important part of the learning experience at the graduate level, and one to which online learners could have little or no exposure until the advent of MOOCs. Because MOOCsters who are stuck on some point often post questions to the courses' community discussion forums, fellow students have the opportunity to provide help. This is in addition to the answers that are given (less frequently) by the "official" course TAs, whose posts are tagged with a special icon. In fact, students who have done well in a previous iteration of a course or who have prior experience may be invited to become "community TAs" with their own special icon. This is not only a laudable service to the course community, it is hugely valuable experience to anyone who would like to achieve more than basic competency in a subject area.

Alternatives. Though also not a MOOC, the University of California, Irvine offers the first half of "Organisms to Ecosystems" by Michael Clegg, which deals with the basics of evolution, on its OpenCourseWare site (http://ocw.uci.edu/courses/biosci_94_organisms_to_ecosystems.html).

Going Further. The U.S. National Institutes of Health has a series of 16 invited lectures on "Evolution and Medicine" (http://videocast.nih.gov/PastEvents.asp?c=64), including talks by the instructors of this course and the one immediately below. The speakers are stellar and the topics are actually much more wide-ranging than medicine, together providing an excellent capstone to the recommended course.

Evolutionary Genetics 💻✓

Mohamed Noor, Duke University "Introduction to Genetics and Evolution" (Coursera, Winter 2013: https://www.coursera.org/course/geneticsevolution)

> *"Evidence for evolution… Introduction to basic genetics… Recombination and genetic mapping simple traits… Complications to genetic mapping… Genes vs. environment… Basic population genetics and Hardy-Weinberg… Gene flow, differentiation, inbreeding… Natural selection and genetic drift… Molecular evolution… Adaptive behaviors and sexual selection… Species formation and phylogenetics… Evolutionary applications and misapplications…"*

Commentary. This course overlaps with the basic Genetics course in the Biology Department insofar as it covers introductory genetics, but only to the extent necessary to engage with the main topic of

population and evolutionary genetics. Even so, it only covers the latter subjects at an introductory level. Nevertheless there are hands-on exercises in mapping, heritability, Hardy-Weinberg analysis, measurement of selective pressure, etc. that make it a worthwhile extension, and the instructor has the personality to keep students involved. Also of interest is a free iPhone app developed by a student in the course that implements several of the quantitative aspects of the course (https://itunes.apple.com/us/app/genetics-and-evolution/id650401749).

Evaluation.

Course Level	*Hours per Week*	*Course Grade*				*Student Grade*	*Curve Pass/"A"*
		Lectures	*Homework*	*Assessment*	*Overall*		
Introductory	6	92	88	88	88	89.4	60/80

Prerequisites. Introduction to Biology.

Textbook. None is required. The *Nature* education site has a brief but well-done introduction to the topic (http://www.nature.com/scitable/ebooks/cntNm-16553748) and an excellent set of articles on both evolutionary (http://www.nature.com/scitable/topic/evolutionary-genetics-13) and population genetics (http://www.nature.com/scitable/topic/population-and-quantitative-genetics-21).

Going Further. We note again that the Broad Institute has a "Primer on Medical and Population Genetics" featuring eleven lectures by their senior staff (http://www.broadinstitute.org/scientific-community/science/programs/medical-and-population-genetics/primers/primer-medical-and-pop).

Computational Evolution ✓

(See listing in Computational Biology Department.)

Introduction to Paleontology

Henning Haack, University of Copenhagen "Origins—Formation of the Universe, Solar System, Earth, and Life" (Coursera, Fall 2014: https://www.coursera.org/course/origins)

> *"The history of our planet and Solar System, during an interval of almost 5 billion years, is controlled by a series of key biological and geological processes. The course will investigate the prehistory and origins of our Solar System, the Earth and its tectonic processes, the origin of life and the evolution of the complex marine and terrestrial ecosystems that have uniquely defined our planet."*

Commentary. Paleontology, the geologic perspective on the origin and evolution of life, is very broadly drawn by the syllabus of this course. For computational biologists, it may be of interest mostly insofar as it sets the stage for other courses.

Going Further. For those who take this course and become interested in geology proper, Coursera offers "Planet Earth" from Stephen Marshak of the University of Illinois at Urbana-Champaign (https://www.coursera.org/course/earth).

Introduction to Paleobiology

Bruce Fouke, University of Illinois at Urbana-Champaign "Emergence of Life" (Coursera, TBA: https://www.coursera.org/course/emergenceoflife)

> *"This course will evaluate the entire history of life on Earth within the context of our cutting-edge understanding of the Tree of Life. This includes reconnaissance of ancient primordial life before the first cell had evolved, the entire ~4-billion-year development of single- and multi-celled life through the lens of the Tree of Life, and the influence of Earth system processes (meteor impacts, volcanoes, ice sheets) on*

shaping and structuring the Tree. This synthesis emphasizes the universality of the emergence of life as a prelude for the search for extraterrestrial life."

Commentary. The starting point for this course is the work of Carl Woese (much of it on the Urbana-Champaign campus hosting this course) that helped establish the molecular basis for our modern understanding of the "tree of life." This makes for useful background to computational approaches to phylogenetic studies of the early stages of evolution.

Textbook. None is required.

Dinosaur Paleobiology

John Currie, University of Alberta "Dino 101: Dinosaur Paleobiology" (Coursera, Fall 2013: https://www.coursera.org/course/dino101)

"… [S]tudents will learn about the many kinds of non-avian dinosaurs that roamed the earth during the Mesozoic Era, from 250 to 65 million years ago. Numerous topics are covered in order to deliver a comprehensive survey of this important group of animals. These include adaptations for attack and defence, anatomy, appearances, behaviors, birth, deep time, evolutionary theory, feeding, fossilization, growth, integumentary structures, locomotion, major groupings, origins, paleogeography, plate tectonics, reproduction, species definition, stratigraphy, and the extinction event that brought their dominance to an end."

Commentary. Needless to say, the subject matter of this course is endlessly fascinating to kids of all ages. The course itself is not as challenging as it could be and indulges a bit too much in multimedia gimmickry at times. However the content is authoritative and there is a core of interesting science, including a bit of modeling that may be of interest to computational types.

Evaluation.

Course Level	*Hours per Week*	*Course Grade*				*Student Grade*	*Curve*
		Lectures	*Homework*	*Assessment*	*Overall*		*Pass/"A"*
Introductory	3	92	85	86	88	100.0	80/—

Textbook. None is required.

Paleoanthropology

Adam van Arsdale, Wellesley College "Introduction to Human Evolution" (edX, Fall 2013: https://www.edx.org/course/wellesleyx/wellesleyx-anth207x-introduction-human-873)

"This course will provide an overview of human evolutionary history from the present—contemporary human variation in a comparative context—through our last common ancestor with the living great apes, some 5-7 million years in the past. Emphasis will be placed on major evolutionary changes in the development of humans and the methodological approaches used by paleoanthropologists and related investigators to develop that knowledge. The course will begin by asking basic questions about how evolution operates to shape biological variation and what patterns of variation look like in living humans and apes. We will then look at how the human lineage first began to differentiate from apes, the rise and fall of the Australopithecines, the origin and dispersal of the genus Homo, and eventually the radical evolutionary changes associated with the development of agricultural practices in the past 15,000 years."

Commentary. This well-produced course offers coverage of the genetic aspects of hominin evolution, including recent selective sweeps, but its greatest strength lies in its very thorough characterization of the fossil evidence with detailed anatomic descriptions. The readings contribute greatly, and the

opportunities for computational intervention are at least covered in outline, not only in the genomic analyses of Neanderthal and Denisovan DNA but also in morphometric characterization of skeletal geometries and demographic modeling.

Personal Note. Any natural history museum will have reconstructions of hominids in various tableaux, in which the student will take renewed interest after taking this course. One of the most impressive such exhibits is the set of 15 sculptures by the artist John Gurche in the Smithsonian National Museum of Natural History in Washington, DC (http://humanorigins.si.edu/exhibit/early-human-reconstructions). They are truly haunting when seen "in the flesh," after the extensive exposure to fossil skulls one gets in this MOOC, and some such museum excursion is highly recommended as a follow-up. Also listen to a recent discussion with Gurche on the radio show Science Friday about "how art and science inform one another" (http://www.sciencefriday.com/segment/03/21/2014/sculpting-science.html), a theme further explored below in the Humanities Department.

Evaluation.

Course Level	*Hours per Week*	*Course Grade*				*Student Grade*	*Curve*
		Lectures	*Homework*	*Assessment*	*Overall*		*Pass/"A"*
Introductory	10	94	95	91	94	95	60/—

Textbook. None is required. Many readings are provided, most drawn from *Nature* education site (http://www.nature.com/scitable/knowledge/biological-anthropology-98009799).

Alternatives. Coursera offers as direct competition the course "Human Evolution: Past and Future" by John Hawks of the University of Wisconsin (https://www.coursera.org/course/humanevolution). Compared to the course above, the instructor is perhaps more of a rock star in the field, and his course is more slickly produced, with visits to field sites and extensive interviews with other workers in the field. (The conversation with Lee Berger is a highlight, particularly for his advocacy of the paleoanthropological version of Open Access.) However, the assessments for the Coursera MOOC are not as thorough-going nor are there course notes or slides available. Otherwise, the courses are so similar in content (including a focus on genetics) as to make the choice primarily one of timing. Better yet, just do both, as the edX version is excellent preparation for the Coursera entry.

Paleopathology

Michael Zimmerman, Villanova University "Paleopathology and the History of Disease" (iTunes U, 2012: https://itunes.apple.com/us/itunes-u/paleopathology-history-disease/id494399959)

> *"Paleopathology is defined as the study of the evidence of disease in ancient human and animal remains. Evidence of disease is based on historic records and the examination of skeletal material and mummies. The history of the field, and principles of anatomy, physiology and biochemistry are reviewed."*

Commentary. This is a non-MOOC series of lectures by an anthropologist who is also a retired pathologist. His delivery is rather dry and the videos are occasionally ragged, but the subject matter holds a certain grim fascination. While the medical material is peripheral to computational biology, it provides some in-depth background for the hot new field of paleogenomics [74]. A taste of the subject can be seen in a talk given by the instructor called "20th Century Paleopathology: a Train Robber and a President" (https://www.youtube.com/watch?v=FgSQvWH9Xyk).

Going Further. Paleopathology can be viewed as forensic science applied to the ultimate in "cold cases." For more of the latter subject, see Coursera's fascinating "Introduction to Forensic Science" by Roderick Wayland Bates of the Nanyang Technological University (https://www.coursera.org/course/ntufsc). It promises to shed light on the question of whether Napoleon was "murdered by the perfidious British, or killed by his wallpaper" as well as considering the Kennedy assassination, the identification of the last

Tsar of Russia, and numerous other lesser known cases. On an even more somber note, it will be noted that the Gene Codes Corporation, an early bioinformatics startup, spun off Gene Codes Forensics, Inc., as a result of its involvement in DNA-based mass fatality identification for the World Trade Center attack (http://genecodes.com/about-gene-codes/history).

Astrobiology 💻✓

Charles Cockell, University of Edinburgh "Astrobiology and the Search for Extraterrestrial Life" (Coursera, 2013: https://www.coursera.org/course/astrobio)

> *"In this course we will explore what we know about life's ability to live in extreme environments on the Earth, we will look at different hypotheses for how it originated. We will look at some of the missions to search for life in our own Solar System and on planets orbiting distant stars. We will discuss some of the extreme environments on the Earth that help us understand the limits of life and how life has adapted to cope with extremes. We will explore the possibility of intelligent alien life and some of the implications of its detection. The course will provide a foundation in astrobiology and introduce students to concepts in a diversity of scientific fields."*

Commentary. This short but comprehensive course spends about equal time on theories of the emergence of life on earth and the possibilities of life elsewhere. The connection to astronomy provides a link to another source of "big data" besides genomics. The instructor has written several books on the subject and provides wonderful visuals.

Evaluation.

Course Level	*Hours per Week*	*Course Grade*				*Student Grade*	*Curve*
		Lectures	*Homework*	*Assessment*	*Overall*		*Pass/"A"*
Introductory	4	95	90	90	93	98.2	65/85

Textbook. None is required, but the course description lists many optional books.

Alternatives. Princeton's David Spergel offers a more extensive 12-week MOOC on the same topics called "Imagining Other Earths" (https://www.coursera.org/course/otherearths).

Going Further. For those who develop an interest in the astronomical aspects of Astrobiology, there is a rigorous "Introduction to Astronomy" offered by Ronen Plesser of Duke University that is very worthwhile and includes a considerably more technical treatment of the topic of exoplanet search (https://www.coursera.org/course/introastro). For a more modest commitment, try "Confronting the Big Questions: Highlights of Modern Astronomy" by Adam Frank of the University of Rochester (https://www.coursera.org/course/astronomy), which has gorgeous visuals.

Current Topics in Paleoanthropology

Various Speakers, University of California, San Diego, Center for Academic Research and Training in Anthropogeny (CARTA Symposia: http://carta.anthropogeny.org/symposia/past_list)

> *"CARTA organizes up to three public symposia each year. These are free, widely advertised meetings that bring together scientists eminent in their respective fields to address a particular aspect of human origins. Presentations are specifically directed towards scientists in other fields and an educated lay audience, attempting to minimize the use of jargon, while highlighting clear and simple messages."*

Commentary. There have been over twenty CARTA symposia going back to 2004, all with online videos available, constituting a gold mine of interdisciplinary work in human origins. Recent instances include "Mind Reading: Human Origins and Theory of Mind," "Behaviorally Modern Humans: The Origin of Us," "Is the Human Mind Unique?," "The Evolution of Human Nutrition," "Human Origins: Lessons from

Autism Spectrum Disorders," "Culture-Gene Interactions in Human Origins," "Uniquely Human Features of the Brain," "The Genetics of Humanness," "The Evolution of Human Altruism," "Early Hominids," and "The Evolution of Human Biodiversity."

Current Topics in Cognitive Evolution

(See listing in Neurosciences Department.)

Evolutionary Biology Seminars

(See seminars at http://www.youtube.com/playlist?list=PL9s5HIfOWP3M1R0hDnpdpp8jFIwUCCwMG)

Systems Biology Department

As is the case in many academic institutions, this Department combines traditional physiology and modern systems biology, but it also includes preparatory courses in modeling and networks. A number of these courses come from the Icahn School of Medicine at Mount Sinai, and have been bundled into a five-part Coursera Specialization that can be taken serially as a unit, together with a "capstone" project (https://www.coursera.org/specialization/systemsbiology/6?utm_medium=listingPage). The component courses are listed here separately so as to provide commentary and offer alternatives. Also represented in this Department, by means of a Current Topics entry, is the blazingly hot field of synthetic biology.

Physiology 💻✓

Jennifer Carbrey and Emma Jakoi, Duke University "Introductory Human Physiology" (Coursera, Winter 2014: https://www.coursera.org/course/humanphysio)

> *"The students learn to recognize and explain the basic concepts that govern each organ and organ system and their integration to maintain homeostasis, as well as some clinical aspects of failure of these systems. The organ systems covered include: nervous, muscle, cardiovascular, respiratory, endocrine, male and female reproductive, gastrointestinal, and urinary."*

Commentary. This course, comprising 31 hours of lectures over 12 weeks, is advertised as sufficient preparation for the U.S. Medical College Admission Test. The course is rather unusual among MOOCs in having "closed book" examinations on the student's honor.

Evaluation.

Course Level	*Hours per Week*	*Course Grade*				*Student Grade*	*Curve*
		Lectures	*Homework*	*Assessment*	*Overall*		*Pass/"A"*
Intermediate	8	90	87	93	91	85.2	?/85

Prerequisites. Introduction to Biology.

Textbook. None is required, though several are recommended and having one would be advisable since the lecture notes provided are slightly hit-or-miss. OpenStax has a free online textbook covering Anatomy and Physiology (http://cnx.org/contents/14fb4ad7-39a1-4eee-ab6e-3ef2482e3e22).

Alternatives. The Carnegie Mellon Open Learning Initiative has a comprehensive "Anatomy & Physiology" offline self-study course, which would also be a good adjunct to the video course above (http://oli.cmu.edu/courses/free-open/anatomy-physiology).

Going Further. Coursera also offers "Exercise Physiology" by Mark Hargreaves of the University of Melbourne (https://www.coursera.org/course/exphys), comprising a short but technical exploration of this popular subfield. For the anatomical side of exercise, edX offers "Musculoskeletal Anatomy" by Trudy van Houten of the Harvard Medical School, which has an amazing array of activities including orthopedic physical exams, anatomical dissections, radiology workups, and virtual surgeries (https://www.edx.org/course/harvardx/harvardx-at1x-musculoskeletal-anatomy-1767).

Introduction to Systems Biology 💻✓

Ravi Iyengar, Icahn School of Medicine at Mount Sinai "Introduction to Systems Biology" (Coursera, Spring 2014: https://www.coursera.org/course/sysbio)

"An introduction to current concepts of how cellular molecules come together to form systems, how these systems exhibit emergent properties, and how these properties are used to make cellular decisions."

Commentary. This course is the starting point for the Coursera Specialization in Systems Biology. It touches on many but not all aspects of systems biology, with special attention to the instructor's areas of expertise, including network motifs and modularity. The assessments include a unique attempt to fit essay questions into a multiple choice format by asking the student to string together a selection of given sentences in the correct order; the experiment is not entirely successful.

Evaluation.

Course Level	*Hours per Week*	*Course Grade*				*Student Grade*	*Curve*
		Lectures	*Homework*	*Assessment*	*Overall*		*Pass/"A"*
Intermediate	8	91	89	85	88	95.0	?/80

Prerequisites. Introduction to Biology. Familiarity with statistics.

Textbook. None is required.

Mathematical Systems Biology

Uri Alon, Weizmann Institute of Science "Systems Biology" (Weizman, Spring 2011: http://www.weizmann.ac.il/mcb/UriAlon/movies/Systems%20Biology%20Course%202014)

"Living cells are a special form of condensed matter, matter that has been optimized by evolution to perform functions. Are there laws of nature that govern living matter, that stem from the 'need' to function? Are there common design-principles shared by living systems and human-made objects that perform functions, such as machines, circuitry and software? This course discusses theoretical concepts and analysis of biological networks. We discuss gene- and protein-circuits as computational devices, and approaches for analysis of large networks. The theoretical discussion is accompanied by examples of well-studied model systems."

Commentary. This course can also be considered an introduction to systems biology, but one taught by a physicist (the 2004 Overton Prize winner Uri Alon), which lends it an entirely different flavor compared to the course above. It has a narrower focus on biological circuits of various kinds, but with a full mathematical treatment. The course is not a MOOC and so has no ancillary material, but the instructor closely follows his textbook (see below) which has plenty of exercises. (The 2011 version of the course is complete, but a 2014 edition was underway as of this writing.)

Prerequisites. Introduction to Biology, Calculus, Differential Equations.

Textbook. *An Introduction to Systems Biology: Design Principles of Biological Circuits* by the instructor [75].

Quantitative Systems Biology

Various Speakers, International Centre for Theoretical Sciences (ICTS), Tata Institute of Fundamental Research "Winter School on Quantitative Systems Biology" (ICTS, Winter 2013: http://www.icts.res.in/program/all/talks_tab/338 or https://www.youtube.com/playlist?list=PL04QVxpjcnjg5Y1vGamUcCJxopAO3a20r)

"New experimental techniques are opening windows on biological mechanisms inside the cell and in the brain, making these systems accessible to quantitative investigation. These advances have shown the importance of the concerted interaction of many agents in producing overall behaviors, and call for an

understanding of biological functions at the systemic level. The present school responds to the need to provide physicists with a broad exposure to quantitative problems in the study of living systems."

Commentary. Like the previous course, this one has a physics orientation. However the adjective "quantitative" in the title, in contrast to the "mathematical" in the previous title, is meant to reflect a wider and more eclectic scope in both the applications covered and the methods applied. It ranges far beyond networks to cover a miscellany of topics from many branches of biology that call for quantitative, systems-oriented approaches. It is more like a coordinated set of seminars than a MOOC, but it is an interesting elective and follows fairly neatly on the previous course.

Prerequisites. Introduction to Biology, Calculus, Differential Equations.

Experimental Systems Biology 💻

Marc Birtwistle, Icahn School of Medicine at Mount Sinai "Experimental Methods in Systems Biology" (Coursera, Fall 2014: https://www.coursera.org/course/expmethods)

" This course will cover techniques in genomics, proteomics, metabolomics, genome wide functional screens, chemical library screens and high content imaging. Each approach will be introduced in the context of the biological questions the approach is used to address. There will be a discussion of the strengths and limitations of the technology. The experiments will be described from start to finish using videos. Treatment protocols and sample preparation will be described. Experts who run cores will describe the machinery and technologies used for these experiments. The raw data is obtained and the methods of statistical analyses will be discussed. Descriptions of experimental methods underlying dynamical models will be described. Genome wide screen for functional assays, including high content imaging and chemical library screening for drug discovery will also be introduced. The course will conclude with the description of publicly available databases."

Commentary. This is another course in the Systems Biology series from the Icahn School of Medicine at Mount Sinai, covering experimental methods used in the field. It would be a useful adjunct to the Genomics course in the Biology Department, covering many of the same technologies but with a different emphasis and orientation.

Prerequisites. Cell Biology, Biochemistry.

Introduction to Networks 💻✓

Lada Adamic, University of Michigan "Social Network Analysis" (Coursera, Fall 2013: https://www.coursera.org/course/sna)

"What are networks and what use is it to study them?... Random network models: Erdos-Renyi and Barabasi-Albert... Network centrality... Community... Small world network models, optimization, strategic network formation and search... Contagion, opinion formation, coordination and cooperation... Cool and unusual applications of SNA... SNA and online social networks."

Commentary. Social networks share important properties with biological networks and this class is an appropriate introduction to those properties and metrics important in network biology, such as node degree and various flavors of centrality. The course uses Gephi and NetLogo for demonstrations and homeworks, and R for the optional programming assignments.

Evaluation.

Course Level	*Hours per Week*	*Course Grade*				*Student Grade*	*Curve*
		Lectures	*Homework*	*Assessment*	*Overall*		*Pass/"A"*
Introductory	6	91	88	88	90	96.8	80/—

Textbook. *Networks, Crowds and Markets* by David Easley and Jon Kleinberg is optional [76] and is also freely available online (http://www.cs.cornell.edu/home/kleinber/networks-book).

Alternatives. Coursera has several other courses on social networks at an introductory level. Mung Chiang and Christopher Brinton of Princeton University teach "Networks Illustrated: Principles without Calculus" (https://www.coursera.org/course/ni), and Michael Kearns of the University of Pennsylvania has a course entitled "Networked Life" (https://www.coursera.org/course/networks).

Going Further. See the previous catalog for additional recommendations in the area of graph theory.

Network Analysis

Matthew Jackson, Stanford University "Social and Economic Networks: Models and Analysis" (Coursera, Winter 2014: https://www.coursera.org/course/networksonline)

> *"The course begins with some empirical background on social and economic networks, and an overview of concepts used to describe and measure networks. Next, we will cover a set of models of how networks form, including random network models as well as strategic formation models, and some hybrids. We will then discuss a series of models of how networks impact behavior, including contagion, diffusion, learning, and peer influences."*

Commentary. Although the description of this course is similar to the introductory course above, it is at a more advanced level and requires significantly more in the way of mathematical sophistication. Once again this course (and nearly all such MOOCs) deal with social networks, but it should be emphasized that the key models and metrics are often relevant to biology, as are processes like formation and diffusion. The instructor also teaches Game Theory, and this course incorporates elements of that topic. Homeworks can be done with either Pajek or Gephi.

Prerequisites. Calculus, Linear Algebra, Probability, Statistics.

Textbook. *Social and Economic Networks* by the instructor [77] is the basis of the MOOC, but is optional.

Alternatives. The edX entry "Networks, Crowds and Markets" by David Easley, Jon Kleinberg, and Éva Tardos (https://www.edx.org/course/cornellx/cornellx-info2040x-networks-crowds-1354) is a very promising alternative, based as it is on their excellent textbook, mentioned in the previous course.

Network Biology ✓

Avi Ma'ayan, Icahn School of Medicine at Mount Sinai "Network Analysis in Systems Biology" (Coursera, Fall 2013: https://www.coursera.org/course/netsysbio)

> *"Students will learn how to construct, analyze and visualize different types of molecular networks, including gene regulatory networks connecting transcription factors to their target genes, protein-protein interaction networks, cell signaling pathways and networks, drug-target and drug-drug similarity networks and other functional association networks. Methods to process raw data from genome-wide RNA (microarrays and RNA-seq) and proteomics (IP-MS and phosphoproteomics) profiling will be presented. Processed data will be clustered, and gene-set enrichment analyses methods will be covered. The course will also discuss topics in network systems pharmacology including processing and using databases of drug-target interactions, drug structure, drug/adverse-events, and drug induced gene expression signatures. "*

Commentary. This course is an intensive and occasionally disjointed overview of methodologies used in analyzing biological networks, with a large number of assignments. A major proportion of these make use of web resources developed in the instructor's own lab. Thus the course is somewhat restricted in its perspective, but this is offset to some degree by the wide breadth of those resources.

Evaluation.

Course Level	Hours per Week	Course Grade				Student Grade	Curve
		Lectures	Homework	Assessment	Overall		Pass/"A"
Intermediate	8	86	85	89	86	100.0	50/75

Prerequisites. Statistics and Molecular Biology useful but not required.

Textbook. None is required.

Introduction to Modeling 💻✓

Scott Page, University of Michigan "Model Thinking" (Coursera, Winter 2014: https://www.coursera.org/course/modelthinking)

> *"Introduction: Why Model?... Sorting and Peer Effects... Aggregation... Decision Models... Models of People: Thinking Electrons... Linear Models... Tipping Points... Economic Growth... Diversity and Innovation... Markov Processes... Lyapunov functions... Coordination and Culture... Path Dependence... Networks... Randomness and Random Walks... The Colonel Blotto Game... The Prisoners' Dilemma and Collective Action... Mechanism Design: Auctions... Learning: Replicator Dynamics... The Many Model Thinker: Diversity and Prediction."*

Commentary. The instructor is a Professor of Complex Systems, Political Science, and Economics at Michigan's Center for the Study of Complex Systems. That makes this is a more broadly ranging approach to modeling than one is used to in computational biology, which in itself may justify its inclusion here. The slides are little more than scratchpads for the instructor's barely legible scrawl, but the excellent readings make up for this and the delivery is spirited.

Evaluation.

Course Level	Hours per Week	Course Grade				Student Grade	Curve
		Lectures	Homework	Assessment	Overall		Pass/"A"
Introductory	8	88	87	93	88	99.4	75/—

Textbook. *Complex Adaptive Systems* by John Miller and the instructor [78], is probably the most appropriate of several books recommended, but is optional given the readings provided.

General Systems Modeling 💻

Todd Murphey, Northwestern University "Everything is the Same: Modeling Engineered Systems" (Coursera, TBA: https://www.coursera.org/course/modelsystems)

> *"Students in this class will learn modeling and analysis techniques applicable to electrical, mechanical, and chemical systems. This 'systems' view, that focuses on what is common to these different physical systems, has been responsible for much of the progress in the last several decades in aeronautics, robotics, and other engineering disciplines where there are many different technologies working together."*

Commentary. While the Introduction to Modeling course above was an economist's and political theorist's take on modeling, this one is from an engineer's viewpoint. It is more quantitative and provides good practice in using differential equations to model interacting collections of components of many different types. The principles carry over to biology, of course.

Prerequisites. Calculus. Differential Equations is helpful but not essential.

Textbook. Online course notes are provided, with additional material.

Biological Systems Modeling 💻

Eric Sobie, Icahn School of Medicine at Mount Sinai "Dynamic Modeling Methods for Systems Biology" (Coursera, Winter 2014: https://www.coursera.org/course/dynamicalmodeling)

> *"This course focuses on dynamical modeling techniques used in Systems Biology research. These techniques are based on biological mechanisms, and simulations with these models generate predictions that can subsequently be tested experimentally. These testable predictions frequently provide novel insight into biological processes. The approaches taught here can be grouped into the following categories: 1) ordinary differential equation-based models, 2) partial differential equation-based models, and 3) stochastic models."*

Commentary. This course introduces the critical mathematical techniques underlying much of the activity in systems biology today, such as modeling of cells, organs, ecosystems, epidemics, the brain, the immune system, and so forth. The techniques employed include ordinary differential equations, partial differential equations, and stochastic models, with particular attention paid to bistability of biochemical signaling and modeling of the cell cycle and electrical signals. The course uses MATLAB, for which free temporary licenses have been arranged; students are required to modify and apply code modules that are presented to them.

Prerequisites. Only calculus is listed as a prerequisite by the instructor, but the syllabus suggests that Differential Equations as well as Probability would be advantageous.

Textbook. Readings are provided from open access journals or e-reprints.

Neuronal Modeling 💻✓

(See listing in Neurosciences Department.)

Graphical Models 💻✓

(See listing in Data Sciences Department.)

Dynamical Systems

(See listing in Mathematics Department.)

Systems Biology Applications 💻

Susana Neves and Ravi Iyengar, Icahn School of Medicine at Mount Sinai "Integrated Analysis in Systems Biology" (Coursera, Spring 2015: https://www.coursera.org/course/integratedanalysis)

> *"This course will serve as the capstone for the sequence which will focus on developing the integrative skills in the students. This will be done through a set of directed reading and analysis of the current primary literature... These analyses will set the stage for a final exam which will be a research project where the student will be given a biological problem and asked to select an experimental strategy and one or more computational strategy, and based on the selections predict the types of results that would be obtained."*

Commentary. This course is intended as the "overall final exam" for the series of systems biology courses from the Icahn School of Medicine at Mount Sinai. Topics to be covered include cell signaling networks, immune signaling, genomics and network analysis in cancer, and dynamical multiscale modeling.

Prerequisites. Cell Biology, Biochemistry, Statistics, Calculus. Given the "capstone" nature of this course it would be advisable to take other Coursera courses in this series from Icahn School of Medicine at Mount Sinai, listed above.

Textbook. Current systems biology literature will be analyzed critically

---

Current Topics in Systems Biology

Various Speakers, National Center for Systems Biology, Annual Meeting (NIH VideoCast, Summer 2013: http://videocast.nih.gov/Summary.asp?File=18036)

> *"The National Centers for Systems Biology provide national leadership in systems biology research, training, and knowledge dissemination. They accomplish this goal through the development of truly innovative programs that establish new research areas and new research culture, by developing and distribute enabling technologies, and by training a new generation of systems biologists... Each year an annual meeting is held to discuss research and educational programs being performed within the Centers."*

Commentary. This annual meeting of the major systems biology centers funded by the U.S. National Institutes of Health has been the occasion for command performances from the leaders in the field. Speakers at the 2013 event included Arthur Lander, Andrew Murray, Ravi Iyengar, David Botstein, Peter Sorger, Gary Churchill, John Aitchison, Kevin White, Bridget Wilson, and Alex Hoffman.

Current Topics in Synthetic Biology

Various Speakers "Fourth New Phytologist Workshop: Synthetic Biology" (University of Bristol, 2012: https://www.youtube.com/playlist?list=PLylDstki6x-p3LHwkTKldrdQBgvWzc6W0)

> *"The overall goal for the workshop was to bring together scientists working in the highly interdisciplinary field of synthetic biology to present cutting-edge research aligned with three key themes – 'Engineering principles and approaches in synthetic biology', 'Synthetic biology in microbes' and 'Synthetic biology in plants'."*

Various Speakers "First International Workshop on Mammalian Synthetic Biology Workshop" (Massachusetts Institute of Technology, 2013: http://mammalian-synbio.org/video)

> *"The general goals of the workshop were to nucleate the nascent mammalian synthetic biology community, reach out to experts from other fields that can benefit from and contribute to this field, and define the important challenges and future directions."*

Commentary. These two workshops together very neatly span the emerging field of synthetic biology, on the one hand covering microbes and plants with a European perspective, and on the other hand mammalian systems with an American slant.

Current Topics in Network Analysis

Various Speakers, The Royal Society "Workshop on Function Prediction in Complex Networks" (videolectures.net, 2012: http://videolectures.net/complexnetworks2012_london)

> *"The aim of the meeting was to bring together researchers from complex networks, and those working in machine learning and graph theory. The goal was to identify current challenges in complex networks analysis and identify possible methodologies for addressing them."*

Commentary. This workshop includes sessions on characterizing complex network structure, dynamic processes on complex networks, and predicting network function. Included are talks on protein-protein interaction networks, ecological webs, and whole animal modeling.

Systems Biology Seminars

(See seminars at http://www.youtube.com/playlist?list=PL9s5HIfOWP3PfE0i59wEfriBqHolRzOHs)

Neurosciences Department

This is another new department in which much has been happening on the MOOC front, after being all but completely absent from the previous catalog. This is felicitous, since neuroinformatics has to be counted as one of the hottest subfields of computational biology.

Introduction to Psychology

Anderson Smith, Georgia Institute of Technology "Introduction to Psychology as a Science" (Coursera, Winter 2014: https://www.coursera.org/course/psy)

> *"[The] course will include discussions of the brain and nervous system, sensation and perception, learning, memory, intelligence and thinking, lifespan development, emotion and motivation, personality, social behavior, behavioral disorders, and psychological treatment of disorders."*

Commentary. An introductory course in psychology is a good entrée into the neurosciences. Many alternatives are available (see below), but this one promises to be a more "scientific" approach than is typical for freshman psychology courses.

Textbook. The course uses the Carnegie Mellon University Open Learning Initiative courseware (http://oli.cmu.edu/courses/free-open/introductory-psychology-course-details). The last-mentioned alternative course below from the Massachusetts Institute of Technology uses a free online textbook by Charles Stangor (http://www.saylor.org/site/textbooks/Introduction%20to%20Psychology.pdf).

Alternatives. Coursera also offers an "Introduction to Psychology" by Steve Joordens of the University of Toronto (https://www.coursera.org/course/intropsych). A non-MOOC alternative is Yale's excellent course by Paul Bloom (http://oyc.yale.edu/psychology/psyc-110). Finally, John Gabrieli of the Massachusetts Institute of Technology has a very well-structured course with all the appurtenances of a MOOC on MIT OpenCourseWare (http://ocw.mit.edu/courses/brain-and-cognitive-sciences/9-00sc-introduction-to-psychology-fall-2011).

Personal Note. The author took the last-mentioned alternative course as an undergraduate and has fond memories of the late Jerry Lettvin's guest lectures. Lettvin was famous for the classic paper "What the Frog's Eye Tells the Frog's Brain" [79], at that time the most cited paper in all of science, which first proposed feature detectors in the retina. Besides this and a number of other advances in neuroscience, Lettvin wrote poetry and famously debated Timothy Leary about drugs. For the author, Lettvin's most memorable talk was one that made a convincing connection between cephalopod biology and ancient Greek myth surrounding Medusa. He was a larger-than-life Renaissance man who was perfectly at ease straddling the arts and sciences (a characteristic discussed further in the Humanities Department).

Prerequisites. None.

Introduction to Neurosciences ✓

Idan Segev, The Hebrew University of Jerusalem "Synapses, Neurons and Brains" (Coursera, Spring 2013: https://www.coursera.org/course/bluebrain)

> *"This course will discuss, step-by-step, how modern molecular, optical, electrical, anatomical and theoretical methods have provided fascinating insights into the operation of the elementary building blocks of brains and, most importantly, how neuronal mechanisms underlie memory and learning processes. We will next discuss why computer simulations are so essential for understanding both neuronal 'life ware' and the emergence of networks dynamics (e.g., as in the 'Blue Brain Project')."*

Commentary. This course is best thought of as an aperitif in anticipation of more extensive study in neuroscience. While it includes a good introduction to the Hodgkin-Huxley and cable equations, it is mostly a rapid-fire sampling of the field at practically every level, delivered with ingratiating verve. Its greatest attraction may be its coverage of the most recent technological developments for probing brain structure and function, where the current excitement in the field is very well conveyed by the instructor.

Evaluation.

Course Level	Hours per Week	Course Grade				Student Grade	Curve
		Lectures	Homework	Assessment	Overall		Pass/"A"
Introductory	6	92	89	90	90	92.2	70/—

Prerequisites. Basic biology.

Textbook. *From Neuron to Brain* by John Nicholls, Robert Martin, Paul Fuchs, David Brown, Mathew Diaond, and David Weisblat [80]. However, the course is not closely tied to it and there are several good introductory neuroscience textbooks; see Neuroanatomy and Neurophysiology below for alternatives.

Alternatives. A more extensive and far more elaborate introduction to the subject is offered by Harvard as "Fundamentals of Neuroscience" on edX (https://www.edx.org/course/harvardx/harvardx-mcb80-1x-fundamentals-925). It relies on a custom web site that unfortunately proves cumbersome, and in fact the entire course is overproduced, with extensive cartoon animations that add little value. However, there are interesting interactive demonstrations and the option of doing home experiments in neuronal signaling by purchasing an impressive product called SpikerBox from the company Backyard Brains (www.BackyardBrains.com), provided you can find a very large cockroach and don't mind vivisecting it.

Introduction to Medical Neurobiology

Peggy Mason, The University of Chicago "Understanding the Brain: The Neurobiology of Everyday Life" (Coursera, TBA: https://www.coursera.org/course/neurobio)

> *"The course will cover three main topics: neuroanatomy, neural communication, and neural systems... Introduction: neurons, development and organization of the human brain... Neuroanatomy: Sensory and motor pathways, nerves... Strokes, traumatic injuries, and diseases... Neural communication: Information transfer in the nervous system... Perception: Vision... The inner ear: Hearing and balance... Voluntary movement: From stumbling to Simon Says... Voluntary movement: Coordination, chunking and habit... Homeostasis: Thermoregulation, sleep, and eating... Executive function: Memory, language, playing well with others."*

Commentary. This is an alternative introduction to neuroscience, in this case more from a human neurological perspective. The instructor teaches medical students, and the name used for this course is taken from the title of her textbook (see below).

Prerequisites. Introduction to Biology.

Textbook. None is required. *Medical Neurobiology* by the instructor [81] is optional.

Neuroanatomy and Neurophysiology ✓

Leonard White, Duke University "Medical Neuroscience" (Coursera, Winter 2014: https://www.coursera.org/course/medicalneuro)

> *"The course provides students an understanding of the essential principles of neurological function, from cellular and molecular mechanisms of neural signaling and plasticity to the organization and function of sensory and motor systems. This course emphasizes the neural and vascular anatomy of the*

human brain and spinal cord, providing an anatomical framework for localizing lesions within the central nervous system. It also emphasizes the neurobiological foundation for understanding cognition, mental illness and disorders of human behavior."

Commentary. This course is designed for first-year graduate students in the health professions, and involves extensive memorization (you will have dreams about the cranial nerves) as well as detailed human brain dissections that are not for the squeamish. It is very well taught and highly rewarding for those who are able to put in the many hours required. Although notes are provided, serious students should not attempt the course without purchasing the suggested text (see below) coauthored by the instructor, which comes bundled with neuroanatomical atlas software that is also essential to effective learning. The assessments are demanding and the final exam makes heavy use of clinical cases.

Evaluation.

Course Level	*Hours per Week*	*Course Grade*				*Student Grade*	*Curve*
		Lectures	*Homework*	*Assessment*	*Overall*		*Pass/"A"*
Intermediate	14	98	98	97	98	95.2	?/90

Personal Note. The author was so taken by this MOOC that his holiday wish list included a life-sized brain model of the sort frequently used by the instructor in his lectures. While these can be even more expensive than the textbook, they really are helpful in understanding the anatomic relationships, and look impressive on one's desk. 3B Scientific sells a range of models (www.a3bs.com).

Prerequisites. Introduction to Biology. Some prior exposure to neuroscience would be an advantage, simply because of the intensity of the coursework.

Textbook. *Neuroscience* by Dale Purves, George Augustine, David Fitzpatrick, William Hall, Anthony-Samuel LaMantia, and the instructor [82] is optional but strongly recommended. The University of Texas Medical School at Houston, under the direction of John Byrne, has developed an amazingly comprehensive free online neuroscience textbook that incorporates many animations and inline assessments (http://neuroscience.uth.tmc.edu).

Clinical Neurology

Daniel Lowenstein, University of California, San Francisco "Introduction to Clinical Neurology" (Coursera, Winter 2014: https://www.coursera.org/course/clinicalneurology)

"This course will provide a basic overview of the most common and important neurological diseases and conditions affecting people worldwide: stroke, epilepsy, headache, back pain, neurodegenerative diseases, movement disorders, alterations of consciousness, nervous system infections, traumatic brain injury, and neuromuscular diseases.... [It] will focus on the fundamentals of the clinical presentation and evaluation of people with these disorders, as well as some of the additional diagnostic approaches and options for treatment."

Commentary. While aimed squarely at medics (and indeed the course can be used for Continuing Medical Education credits), this MOOC is accessible to anyone with "a basic knowledge of neuroanatomy and neurophysiology." The most serious students of the neurosciences, even if their primary interest is computational in nature, may find this a very worthwhile excursion.

Evaluation.

Course Level	*Hours per Week*	*Course Grade*				*Student Grade*	*Curve*
		Lectures	*Homework*	*Assessment*	*Overall*		*Pass/"A"*
Introductory	5	93	85	93	92	96.8	70/—

Prerequisites. Neuroanatomy and Neurophysiology is helpful.

Textbook. Several expensive books (and an app) are recommended, but the course is actually self-contained.

Neuropharmacology 💻✓

(See listing in Translational Sciences Department.)

Systems Neuroscience 💻

Jennifer Groh, Duke University "The Brain and Space" (Coursera, Spring 2014: https://www.coursera.org/course/brainspace)

> *"Recognizing your mother, finding your phone, going to the grocery store, playing the banjo—these require careful sleuthing and coordination across different sensory and motor domains. This course traces the brain's detective work to create this sense of space and argues that the brain's spatial focus permeates our cognitive abilities, affecting the way we think and remember."*

Commentary. This course is actually only a slice of what can be considered Systems Neuroscience, covering those aspects that overlap with the field of perception. However it should be typical of the approaches and methodologies required for a systems view of the brain, including such notions as brain maps, rate coding, memory, and generalizations from the study of spatial thinking. This should make it attractive to computationally oriented students, especially since the instructor is a computational neuroscientist as well as an experimentalist.

Textbook. The course is organized around the instructor's upcoming book *Making Space: How the Brain Knows Where Things Are* [83], currently available for pre-order (and obviously optional).

Circuits and Electronics 💻

Anant Agarwal, Gerald Sussman, Piotr Mitros, Chris Terman, and Taia Khanna, Massachusetts Institute of Technology "Circuits and Electronics" (edX, Fall 2013: https://www.edx.org/course/mitx/mitx-6-002x-circuits-electronics-1130)

> *"The course introduces engineering in the context of the lumped circuit abstraction. Topics covered include: resistive elements and networks; independent and dependent sources; switches and MOS transistors; digital abstraction; amplifiers; energy storage elements; dynamics of first- and second-order networks; design in the time and frequency domains; and analog and digital circuits and applications."*

Commentary. This material will be useful for the most serious students of neurosciences, not only to better understand the Hodgkin-Huxley and cable equations as models of nerve conduction, but for the exposure to signal processing and the use of modularization and abstraction in engineering. This course, led by the head of the edX program at MIT, is intensive and includes online laboratories.

Personal Note. As an undergraduate the author took several electronics courses in this department, and as a graduate student found the experience invaluable in building biophysical instrumentation, even making a business of it on the side. Given the importance of laboratory instrumentation and robotics to bioinformatics, this course should also be of interest in other domains besides neuroscience.

Prerequisites. Physics (Electricity and Magnetism), Calculus, Linear Algebra, some Differential Equations.

Textbook. *The Foundations of Analog and Digital Electronic Circuits* by Anant Agarwal and Jeffrey Lang [84]. The book is being made available for free online use during the course. The course text for the Rice University alternative below is also online (http://cnx.org/content/col10040/latest).

Alternatives. A non-MOOC version of the course is available through MIT OpenCourseware (http://ocw.mit.edu/courses/electrical-engineering-and-computer-science/6-002-circuits-and-electronics-spring-2007). Coursera has a similar "Fundamentals of Electrical Engineering" by Don Johnson of Rice University (https://www.coursera.org/course/eefun), also rather intensive. For a more biological introduction to electrical principles, Coursera also offers "Bioelectricity: A Quantitative Approach" by Duke University's Roger Barr (https://www.coursera.org/course/bioelectricity), but it must be said that the assessments and some other aspects of this course had issues the first time through.

Computational Neuroscience

Rajesh Rao and Adrienne Fairhall, University of Washington "Computational Neuroscience" (Coursera, Spring 2013: https://www.coursera.org/course/compneuro)

> *"This course provides an introduction to basic computational methods for understanding what nervous systems do and for determining how they function. We will explore the computational principles governing various aspects of vision, sensory-motor control, learning, and memory. Specific topics that will be covered include representation of information by spiking neurons, processing of information in neural networks, and algorithms for adaptation and learning."*

Commentary. This course covers a broad range of theoretical and computational neuroscience, roughly following the outline of what is a standard textbook in the field (see below). The course tends to lurch a bit between fairly gentle introductory material and sudden doses of nontrivial math, but overall it is a good starting point for further work. Homeworks require MATLAB/Octave, but are far from overwhelming. Especially helpful are guest lectures by several workers in the field who provide different perspectives.

Evaluation.

Course Level	Hours per Week	Course Grade				Student Grade	Curve
		Lectures	Homework	Assessment	Overall		Pass/"A"
Intermediate	8	90	88	93	90	100.0	60/—

Prerequisites. Linear Algebra, Calculus, and Probability. Familiarity with MATLAB or Octave is useful.

Textbook. *Theoretical Neuroscience: Computational and Mathematical Modeling of Neural Systems* by Peter Dayan and Laurence Abbott [85].

Neuronal Modeling

Wulfram Gerstner, École Polytechnique Fédérale de Lausanne "Neuronal Dynamics—Computational Neuroscience of Single Neurons" (edX, Fall 2013: https://www.edx.org/course/epflx/epflx-bio465x-neuronal-dynamics-1024)

> *"This course gives an introduction to the field of theoretical and computational neuroscience with a focus on models of single neurons. Neurons encode information about stimuli in a sequence of short electrical pulses (spikes). Students will learn how mathematical tools such as differential equations, phase plane analysis, separation of time scales, and stochastic processes can be used to understand the dynamics of neurons and the neural code."*

Commentary. This very substantial course is focused specifically on mathematical modeling of neurons and has relatively little empirical content. The delivery is cogent and precise, but many of the important details are left to the instructor's commentary and hand annotations of the slides. The course should be

easier to follow after the publication of his new textbook (see below). The homeworks are challenging, and the final exam is a killer, though the student is given two chances.

Evaluation.

Course Level	Hours per Week	Course Grade				Student Grade	Curve
		Lectures	Homework	Assessment	Overall		Pass/"A"
Advanced	10	90	87	90	88	80.0	35/85

Prerequisites. Calculus and Differential Equations. Probability and Statistics would be helpful.

Textbook. *Neuronal Dynamics* by the instructor, Werner Kistler, Richard Naud, and Liam Paninski [86]. For the first iteration of the course, the first chapter was made available online. An earlier book by the instructor and Werner Kistler, *Spiking Neuron Models* [87], is freely available online in HTML format (http://lcn.epfl.ch/~gerstner/BUCH.html).

Behavioral Biology

Robert Sapolsky, Stanford University "Human Behavioral Biology" (Stanford, Spring 2010: https://www.youtube.com/playlist?list=PL848F2368C90DDC3D)

> *"How to approach complex normal and abnormal behaviors through biology. How to integrate disciplines including sociobiology, ethology, neuroscience, and endocrinology to examine behaviors such as aggression, sexual behavior, language use, and mental illness."*

Commentary. This introductory, non-MOOC course actually covers a wide swath of evolution, molecular genetics, and neuroscience. The instructor is a neuroendocrinologist, a primatologist, and above all a charismatic lecturer.

Textbook. Unfortunately, the original classroom version involved readings that are not available. The topics are so eclectic that naming a textbook is impractical. However, for considerably more neurological detail see the free online book *Neurobiology of Sensation and Reward* edited by Jay Gottfried (http://www.ncbi.nlm.nih.gov/books/NBK92797).

Alternatives. Coursera's "Animal Behaviour" by Raoul Mulder and Mark Elgar of the University of Melbourne is very well designed, and although a more traditional approach to behavior, it is mostly experimental rather than descriptive (https://www.coursera.org/course/animalbehav). The genetic aspects of human behavior are covered in Coursera's "Introduction to Human Behavioral Genetics" by Matt McGue of the University of Minnesota (https://www.coursera.org/course/behavioralgenetics).

Going Further. Also, see the entry Neuroeconomics below.

Neuroeconomics

Vasily Klucharev, Higher School of Economics, National Research University (Russia) "Introduction to Neuroeconomics: How the Brain Makes Decisions" (Coursera, Summer 2014: https://www.coursera.org/course/neuroec)

> *"Economics, psychology, and neuroscience are converging today into a unified discipline of Neuroeconomics with the ultimate aim of providing a single, general theory of human decision making. Neuroeconomics provides economists, psychologists and social scientists with a deeper understanding of how they make their own decisions, and how others decide."*

Commentary. This promises to be a fairly comprehensive course in one of the trendiest interdisciplinary topics of the last few years. It combines the latest brain-imaging methods with decision theory, which itself has spawned a slew of Nobel Prizes in economics. Not only are there plenty of

quantitative and computational aspects to this new field, but there are very likely to be plenty of jobs as well.

Textbook. None is required but several chapters in *Neuroeconomics: Decision Making and the Brain* by Paul Glimcher and Ernst Fehr [88] are recommended. An older book by Paul Glimcher, *Decisions, Uncertainty, and the Brain: The Science of Neuroeconomics* [89], is now very reasonably priced.

Going Further. For a somewhat broader look at behavioral economics, consider the edX course "Behavioural Economics in Action" taught by Dilip Soman of the University of Toronto (https://www.edx.org/course/university-torontox/university-torontox-be101x-behavioural-1009). For insight into how people are already making money in this arena, see "An Introduction to Consumer Neuroscience and Neuromarketing" by Thomas Zoëga Ramsøy of Copenhagen Business School (https://www.coursera.org/course/neuromarketing).

Consciousness

John Kihlstrom, University of California, Berkeley "Scientific Approaches to Consciousness" (Berkeley Webcasts, Spring 2013: http://webcast.berkeley.edu/playlist#c,d,Cognitive_Science,-XXv-cvA_iCn782RS__ZxZOafDB-Sze1 or http://www.youtube.com/view_play_list?p=-XXv-cvA_iCn782RS__ZxZOafDB-Sze1)

> *"This course will examine the nature of human consciousness from the interdisciplinary perspective of cognitive science. It will cover topics from the philosophy of mind, cognitive linguistics, neuroscience, psychology, and computational models."*

Commentary. This is an upper-division course in the well-regarded Cognitive Science Department at Berkeley. Computational types will likely be fascinated by the highly interdisciplinary field surrounding the phenomenon of human consciousness, which over the years has attracted eminent philosophers, linguists, and biologists including two Nobel laureates, Francis Crick and Gerald Edelman, who famously switched their careers to neurosciences. While the Berkeley webcast resource lacks the accessory features of true MOOCs, the instructor intends his lectures to be available to an external audience and in past years has offered access to ancillary materials to those who contact him.

Prerequisite. Introduction to Psychology.

Textbook. *Consciousness: The Science of Subjectivity* by Antti Revonsuo [90] is the primary text.

Going Further. Those who find themselves diverted by the philosophical aspects of consciousness may also want to try the Philosophy of Mind course in the Humanities Department, below. For a really offbeat approach to consciousness, the best-selling author and scholar Robert Wright, currently at Princeton University, investigates the Buddhist doctrine of the self (or lack thereof) through the lens of current evolutionary psychology in the Coursera offering "Buddhism and Modern Psychology" (https://class.coursera.org/psychbuddhism).

Current Topics in Cognitive Evolution

Various Speakers, Oxford University "New Thinking: Advances in the Study of Human Cognitive Evolution" (Oxford iTunes U, Summer 2011: https://itunes.apple.com/us/itunes-u/new-thinking-advances-in-study/id459118882?mt=10)

> *"An interdisciplinary conference focusing on new ideas and discoveries in research on the evolution of human cognition. The conference focuses on genetic, developmental, and socio-cultural processes that have played a particularly significant role in the evolution of human cognition, and on uniquely human*

cognitive achievements in domains such as causal understanding, language, social learning, theory of mind and metacognition."

Commentary. This conference comprised ten talks including "The Social Brain on the Internet, "Why the Human Cognitive Niche Was and Is a Crucially Socio-Cognitive Niche," "Metacognition and the Social Mind: How Individuals Interact at the Neural Level," "Experiencing Language," "Signals, Honesty, and the Evolution of Language," "Embodiment: Taking Sociality Seriously," "Cortico-Cerebellar Evolution and the Distributed Neural Basis of Cognition," "A New Comparative Psychology," "The Mystery of Cumulative Culture," and "Cultural Inheritance of Cultural Learning."

Neurosciences Seminars

(See seminars at http://www.youtube.com/playlist?list=PL9s5HIfOWP3O8duZxxndf3i8AJ1O1gGnl)

Translational Sciences Department

Translational Sciences is a broadly defined term that encompasses the movement of therapies from research to application, or "bench to bedside." We include in this new department drug discovery (not only the academic concerns of translational research but also the scientific, regulatory and business aspects of pharmaceutical research and development) and facets of healthcare where informatics has a significant role. We take these to include traditional medical informatics, health informatics (which deals with electronic health records), clinical trials (both data management and statistical analysis), biomedical engineering and imaging, genomic and personalized medicine, healthcare technologies, healthcare policy, and healthcare entrepreneurship.

Spanning the concerns of this Department from an informatics viewpoint is an open access book published by this journal, edited by Maricel Kann and Fran Lewitter, entitled *Translational Bioinformatics* (www.ploscollections.org/translationalbioinformatics). Those interested in approaching translational sciences from a computational standpoint would be well advised to make this collection a focal point.

Introduction to Drug Discovery

Williams Ettouati and Joseph Ma, University of California, San Diego "Drug Discovery, Development & Commercialization" (Coursera, Fall 2014: https://www.coursera.org/course/drugdiscovery)

> *"Students will learn the process of drug discovery and development through specific examples of case studies to better understand the issues facing the challenges of delivering a new drug on the market. At the completion of this course you will be able to have a better understanding of how a small or large molecule becomes a pharmaceutical drug."*

Commentary. This course comprises a series of guest lectures by experts from both academia and industry, each focused on a particular phase of drug discovery, development, and marketing. (Among the speakers is the founding editor of this journal, Phil Bourne.) As a major element of the course, students form teams and simulate the entire process for an existing marketed drug; such collaboration may not be to everyone's taste as a form of assessment, but of course the lectures are available to be audited.

Personal Note. In moving from a faculty position to the pharmaceutical industry, the author was most struck by the eclecticism of the latter. The project teams in this MOOC are meant to have input from a very wide range of disciplines, which is absolutely the case in drug companies (though the input from marketing to early R&D is a bit more indirect in reality). Interdisciplinarity is still sometimes seen as novel in academia, but it has long been a way of life in pharma and especially biotech, where the business side cohabits even more intimately with the science. Computational biologists, whose very existence is based on interdisciplinarity, may find that they adapt especially well to this way of working, and to the wide scope of concerns exemplified by this catalog.

Alternatives. Much the same material is covered in the edX entry "Take your Medicine—The Impact of Drug Development" by Janet Walkow and colleagues from the University of Texas at Austin (https://www.edx.org/course/utaustinx/utaustinx-ut-4-01x-take-medicine-impact-669).

Pharmacology

Emma Meagher, University of Pennsylvania "Fundamentals of Pharmacology" (Coursera, TBA: https://www.coursera.org/course/pharm101)

"This set of courses will discuss the discipline of pharmacology and its integration throughout medical science. Specifically, the content will be organized as follows: 1) Basic Pharmacological Principles; 2) Applied Pharmacology, the concept of applying the basic principles to each organ system with an emphasis on melding pathophysiology with biologic targets for drug therapy; 3) Therapeutics, considered to be the clinical application of applied pharmacology, including the financial implications of therapy, evidence-based medicine, and the limitations of drug therapy and future directions of therapeutics in all disease states, as well as the legal implications of prescription writing; and 4) Advanced Pharmacological Principles, such as cancer therapeutics."

Commentary. This course has not had a repeat performance since its first run. Given the importance of the topic, one hopes that it will be revived soon.

Alternatives. Steven Farmer of the University of Hawaii, Maui College has a very practical non-MOOC course in "General Pharmacology" for nursing and allied health professions, which covers specific drugs and conditions exhaustively (http://www.youtube.com/playlist?list=PLA60863814382D93D).

Neuropharmacology 💻✓

Henry Lester, California Institute of Technology "Drugs and the Brain" (Coursera, Winter 2012: https://www.coursera.org/course/drugsandbrain)

"The neuroscience of drugs for therapy, for prevention, and for recreation. Drug addiction and drug abuse. You'll learn the prospects for new generations of medications in neurology, psychiatry, aging, and treatment of substance abuse."

Commentary. The course covers introductory neuropharmacology at the level of receptors and synapses, with reasonably good exposure to current research on the molecular biology of psychiatric and neurologic disorders, and addiction in particular. Its focus is mechanistic rather than quantitative. As a side benefit, the very genteel instructor provides mini-tours of the CalTech campus.

Evaluation.

Course Level	*Hours per Week*	*Course Grade*				*Student Grade*	*Curve*
		Lectures	*Homework*	*Assessment*	*Overall*		*Pass/"A"*
Introductory	6	92	89	91	90	95.9	70/—

Prerequisites. Molecular Biology.

Textbook. *Molecular Neuropharmacology: A Foundation for Clinical Neuroscience* by Eric Nestler, Steven Hyman, and Robert Malenka [91] is recommended but not required.

Going Further. For a still more focused look at addiction, Michael Kuhar of Emory University offers "The Addicted Brain" on Coursera (https://www.coursera.org/course/addictedbrain).

Medicinal Chemistry 💻✓

(See listing in Chemistry Department.)

Cheminformatics

(See listing in Chemistry Department.)

Health Informatics 💻✓

Mark Braunstein, Georgia Institute of Technology "Health Informatics in the Cloud" (Coursera, Winter 2013: https://www.coursera.org/course/healthinformatics)

> *"This course is intended to help even non-technically trained students gain basic proficiency in health informatics: the application of computing to healthcare delivery, public health and community-based clinical research… Weeks 1-2 cover the US healthcare delivery system's unique structural, economic and policy issues and how they create a potentially strategic role for health informatics. Weeks 3-5 explain at a high level the core technologies involved in contemporary health informatics. Weeks 6-8 explore how these technologies are being deployed using some of the best commercial and open source products as examples. Weeks 9-10 present the technologies that have been developed to explore the digital data provided by these new systems with several examples of cutting edge research using 'big data'."*

Commentary. The instructor has a long history in health informatics, having developed one of the very first electronic health records and several other innovations. The course is not technically deep (other than a brief foray into public key cryptography), but it is detailed and wide-ranging. Moreover there are numerous interviews with other academics and vendors, both established and startup, that give a particularly good perspective on business aspects of the field, in addition to the governmental policy elements.

Evaluation.

Course Level	*Hours per Week*	*Course Grade*				*Student Grade*	*Curve*
		Lectures	*Homework*	*Assessment*	*Overall*		*Pass/"A"*
Introductory	8	92	90	94	93	92.0	70/90

Alternatives. Coursera also offers a less technical "Interprofessional Healthcare Informatics" from Karen Monsen of the University of Minnesota (https://www.coursera.org/course/newwayhealthcare), which puts more emphasis on the viewpoints of practitioners. MIT OpenCourseWare has "Health Information Systems to Improve Quality of Care in Resource-Poor Settings" by Leo Celi, Hamish Fraser, Peter Szlovits, and Ken Paik (http://ocw.mit.edu/courses/health-sciences-and-technology/hst-s14-health-information-systems-to-improve-quality-of-care-in-resource-poor-settings-spring-2012), who examine ways that informatics can contribute in developing countries. The Carnegie-Mellon Open Learning Initiative is in the process of deploying "Healthcare Information and Management Systems" (http://oli.cmu.edu/courses/free-open/healthcare-information-and-management-systems), which is not video-based but has many interactive features and can lead to a professional certification.

Prerequisites. Basic computer skills.

Textbook. *Health Informatics in the Cloud* by the instructor [92] is the basis for the course.

Infectious Disease Dynamics 💻✓

Marcel Salathé, Ottar Bjornstad, Rachel Smith, Mary Poss, David Hughes, Peter Hudson, Matthew Ferrari, and Andrew Read, Pennsylvania State University "Epidemics—the Dynamics of Infectious Disease" (Coursera, Fall 2013: https://www.coursera.org/course/epidemics)

> *"This course will cover key concepts that relate to the emergence, the spread, and the control of infectious disease epidemics… The basics: history of infectious diseases, basic concepts of disease dynamics, parasite diversity, evolution & ecology of infectious diseases… Emergence of diseases: The basic reproductive number, critical community size, epidemic curve, zoonoses, spill over, human/wildlife interface, climate change, hot zones, pathology… Spread of diseases: transmission types (droplets, vectors, sex), superspreading, diffusion, social networks, nosomical transmission, manipulation of*

behavior… Control of diseases: drug resistance, vaccination, herd immunity, quarantines, antibiotics, antivirals, health communication, ethical challenges of disease control… The future of infectious diseases: Evolution of virulence, emergence of drug resistance, eradication of diseases, medicine & evolution, crop diseases & food security, digital epidemiology."

Commentary. This beautifully produced course covers infection dynamics in some detail, and is a good introduction to quantitative aspects of the epidemic spread of disease. It does not go very deeply into the math, but it does cover the basic models and makes a connection with social network approaches. The course has associated with it two optional simulation games.

Evaluation.

Course Level	Hours per Week	Course Grade				Student Grade	Curve
		Lectures	Homework	Assessment	Overall		Pass/"A"
Introductory	5	92	89	90	90	96.0	80/—

Textbook. Links are provided to relevant scientific articles through the course.

Going Further. Darwin College of Cambridge University is running a lecture series this year on "Plagues," available on iTunes U, which approaches its subject in a highly multi-disciplinary way (https://itunes.apple.com/us/itunes-u/darwin-college-lecture-series/id806139095). Also from Cambridge on iTunes U is an extensive series of advanced seminars on "Infectious Disease Dynamics" given at the Isaac Newton Institute for Mathematical Sciences (https://itunes.apple.com/us/itunes-u/infectious-disease-dynamics/id833385368).

Epidemiology and Biostatistics 💻

Earl Francis Cook and Marcello Pagano, Harvard School of Public Health "Health in Numbers: Quantitative Methods in Clinical & Public Health Research" (edX, Fall 2012: https://www.edx.org/course/harvardx/harvardx-ph207x-health-numbers-354)

> *"Principled investigations to monitor and thus improve the health of individuals are firmly based on a sound understanding of modern quantitative methods. This involves the ability to discover patterns and extract knowledge from health data on a sample of individuals and then to infer, with measured uncertainty, the unobserved population characteristics. This course will address this need by covering the principles of biostatistics and epidemiology used for public health and clinical research. These include outcomes measurement, measures of associations between outcomes and their determinants, study design options, bias and confounding, probability and diagnostic tests, confidence intervals and hypothesis testing, power and sample size determinations, life tables and survival methods, regression methods (both, linear and logistic), and sample survey techniques."*

Commentary. With the increasing importance of translational studies, even molecular computational biologists would be well advised to learn the rudiments of statistical methods and conventions associated with human studies. This course and the following one from Harvard's School of Public Health work well together for this purpose.

Textbook. *Principles of Biostatistics* by Marcello Pagano (one of the instructors) and Kimberlee Gavreau [93]. The book is made available to students for free during the course.

Alternatives. Coursera has a more basic introduction to epidemiology by Karin Yeatts and Lorraine Alexander of the University of North Carolina (https://www.coursera.org/course/epidemiology). Also on Coursera, Michael Oakes of the University of Minnesota has a course on "Social Epidemiology" which takes a broad perspective on national policies and global determinants of health (https://www.coursera.org/course/socialepi).

Clinical Trials 💻✓

James Ware, Elliott Antman, Julie Buring, Graham McMahon, Marcia Testa, and Robert Truog, Harvard School of Public Health “Fundamentals of Clinical Trials” (edX, Fall 2013: https://www.edx.org/course/harvardx/harvardx-hsph-hms214x-fundamentals-941)

> *“This course will provide an introduction to the scientific, statistical, and ethical aspects of clinical trials research. Topics include the design, implementation, and analysis of trials, including first-in-human studies (dose-finding, safety, proof of concept, and Phase I), Phase II, Phase III, and Phase IV studies. All aspects of the development of a study protocol will be addressed, including criteria for the selection of participants, treatments, and endpoints, randomization procedures, sample size determination, data analysis, and study interpretation. The ethical issues that arise at each phase of therapy development will be explored.”*

Commentary. This is a comprehensive and well-taught course on all aspects of clinical trials, including a healthy dose of ethics. Assessment, besides frequent quizzes, is by way of commenting on case studies, though these are not very rigorously graded. Although the statistical analyses presented are fairly self-contained, a full understanding of them really does require a more basic course in research statistics, though not necessarily epidemiology.

Evaluation.

Course Level	*Hours per Week*	*Course Grade*				*Student Grade*	*Curve*
		Lectures	*Homework*	*Assessment*	*Overall*		*Pass/"A"*
Intermediate	8	96	90	88	93	99	80/—

Prerequisites. Statistics and/or Epidemiology and Biostatistics.

Textbook. *Fundamentals of Clinical Trials* by Lawrence Friedman, Curt Furberg and David DeMets [94] is optional.

Alternatives. Johns Hopkins has a similar but shorter and less detailed course on Coursera from Janet Holbrook and Lea Drye, called “Design and Interpretation of Clinical Trials,” which even follows the same (optional) textbook (https://www.coursera.org/course/clintrials). From the Karolinska Institute, Merrick Zwarenstein, Vinod Diwan, and Salla Atkins offer “Pragmatic Randomized Controlled Trials in Health Care” on edX (https://www.edx.org/course/kix/kix-kipractihx-pragmatic-randomized-1541).

Going Further. The National Institutes of Health Clinical Center has a comprehensive video training course “Principles and Practice of Clinical Research” that includes slides and other useful materials (http://ippcr.nihtraining.com/archive.php?year=2012). It covers much of the same ground as the recommended course above but some topics are covered in greater detail, and there is even a mock IRB (Institutional Review Board) meeting. Special concerns are addressed in Coursera’s “Vaccine Trials: Methods and Best Practices” by Karen R. Charron and Amber Bickford Cox of the Johns Hopkins University (https://www.coursera.org/course/vacctrials). This course provides a detailed look at Good Clinical Practice standards and the dreaded U.S. Code of Federal Regulations 21 CFR.

Clinical Data Management 💻✓

Stephany Duda, Paul Harris, and Firas Wehbe, Vanderbilt University “Data Management for Clinical Research” (Coursera, Fall 2013: https://www.coursera.org/course/datamanagement)

> *“This course is designed to teach important concepts related to research data planning, collection, storage and dissemination. Instructors will offer information and best-practice guidelines for 1) investigator-initiated & sponsored research studies, 2) single- & multi-center studies, and 3) prospective data collection & secondary-reuse of clinical data for purposes of research. The curriculum will balance*

theoretical guidelines with the use of practical tools designed to assist in planning and conducting research. Real-world research examples, problem solving exercises and hands-on training will ensure students are comfortable with all concepts."

Commentary. The Vanderbilt group are leaders in clinical data management, in part due to their development of the very popular REDCap (Research Electronic Data Capture) clinical trials management system. The course is closely tied to learning REDCap, but this actually proves to be a good way to introduce the wider issues surrounding clinical data.

Evaluation.

Course Level	*Hours per Week*	*Course Grade*				*Student Grade*	*Curve Pass/"A"*
		Lectures	*Homework*	*Assessment*	*Overall*		
Introductory	7	93	89	90	92	96.3	65/90

Prerequisites. Basic computer skills.

Textbook. None is required.

Biomedical Engineering

W. Mark Saltzman, Yale University "Frontiers of Biomedical Engineering" (Open Yale, Spring 2008: http://oyc.yale.edu/biomedical-engineering/beng-100)

"The course covers basic concepts of biomedical engineering and their connection with the spectrum of human activity. It serves as an introduction to the fundamental science and engineering on which biomedical engineering is based. Case studies of drugs and medical products illustrate the product development-product testing cycle, patent protection, and FDA approval."

Commentary. This course is not a MOOC (though many of the Open Yale courses are being retrofitted to the Coursera platform). Much of it is a review of basic physiology, but taught from an engineering perspective, making it more amenable to the informatics mindset. There is brief coverage of biomedical imaging, treated more fully in the next course.

Textbook. The syllabus lists a number of books (http://oyc.yale.edu/biomedical-engineering/beng-100#syllabus), mostly by the instructor.

Alternatives. Yaakov Nahmias of the Hebrew University of Jerusalem teaches a comprehensive "Introduction to Tissue Engineering" on Coursera (https://www.coursera.org/course/tissue101). This course may be preferred to the one above because it is a MOOC, and it is only listed as an alternative because it is more narrowly focused.

Biomedical Imaging

Graham Galloway, David Reutens, Ian Brereton, Gail Durbridge, Karine Mardon, Damion Stimson, Rajiv Bhalla, and Andrew Janke, University of Queensland "Introduction to Biomedical Imaging" (edX, Spring 2014: https://www.edx.org/course/uqx/uqx-bioimg101x-introduction-biomedical-1429)

"This course provides an introduction to biomedical imaging and modern imaging modalities. The course also covers the basic scientific principals behind each modality, and introduces some of the key applications, from neurological diseases to cancers."

Commentary. The course, which is quite comprehensive in its coverage though introductory in nature, includes both basic modules at an elementary level and advanced modules more appropriate for those with a quantitative and/or informatics background. Included are some simple interactive features.

Prerequisites. Calculus, Introduction to Computer Science (for the advanced modules).

Textbook. None is required. The course "Biomedical Signal and Image Processing" at the Massachusetts Institute of Technology has a detailed set of course notes (http://ocw.mit.edu/courses/health-sciences-and-technology/hst-582j-biomedical-signal-and-image-processing-spring-2007/lecture-notes).

Going Further. Martin Lindquist of Johns Hopkins University teaches "Statistical Analysis of fMRI Data" on Coursera (https://www.coursera.org/course/fmri), which requires knowledge of statistics and regression analysis. It stresses methods for dealing with noisy, high-dimensional data that can carry over to other "big data" applications. Another resource is the 2011 symposium at the U.S. National Institutes of Health on "Advanced Medical Imaging Developments and Applications for Neuroscience Research" (http://videocast.nih.gov/launch.asp?16712).

Genomic Medicine 💻

Jeanette McCarthy and Robert Nussbaum, University of California, San Francisco "Genomic and Precision Medicine" (Coursera, Spring 2014: https://www.coursera.org/course/genomicmedicine)

> *"Our course aims to provide participants with some baseline knowledge of genomics, an overview of the clinical applications of genomic medicine, the skills to evaluate the clinical validity and utility of new tests, and an appreciation of the associated ethical and social issues inherent in this field."*

Commentary. This course is aimed at physicians, and in fact qualifies for Continuing Medical Education credits, but will be accessible to those with a background in biology and genetics. The term "precision medicine" refers to the use of a patient's genomic information to provide targeted treatment tailored to the individual, one of the most exciting trends in medicine today and one that will clearly make heavy use of informatics. After the introductory material, the lectures are satisfyingly pithy, and assessment is based on multiple-choice quizzes and short peer-graded responses.

Personal Note. While on the faculty of the University of Pennsylvania School of Medicine in the early 1990s, Dr. Nussbaum had a great deal to do with establishing academic bioinformatics at that institution, in part by recruiting the author to the genetics faculty. At that time informatics was seen as crucial to the mapping and sequencing of the human genome, and now two decades later it is equally important in the effective exploitation of that information in the clinic.

Prerequisites. Introduction to Biology, Genetics.

Textbook. None is required. The only readings are an extensive review by one of the instructors (https://d396qusza40orc.cloudfront.net/genomicmedicine/McCarthy-GenomicMedicineADecade.pdf) and an educational booklet from the National Institutes of Health entitled *The New Genetics* (http://publications.nigms.nih.gov/thenewgenetics/index.html). The *Nature* education site has a huge collection of relevant articles (http://www.nature.com/scitable/topic/genes-and-disease-17).

Alternatives. Georgetown University offers "Genomic Medicine Gets Personal" on edX, taught by a widely interdisciplinary range of faculty (https://www.edx.org/course/georgetownx/georgetownx-medx202-01-genomic-medicine-837), but this appears to be a much more introductory course than the one recommended above. For a distinctly clinical slant, the Stanford School of Medicine has released a series of ten lectures in Genomic Medicine that the faculty prepared for their pathology residents (http://www.youtube.com/playlist?list=PLfTljtR5bxMcTg8hgQp9sA4YQwicpSAQv&feature=c4-feed-u).

Biomedical Ethics 💻

Tom Beauchamp, John Keown, Rebecca Kukla, Margaret Little, Madison Powers, Karen Stohr, and Robert Veatch, Georgetown University "Introduction to Bioethics" (edX, Spring 2014: https://www.edx.org/course/georgetownx/georgetownx-phlx101-01-introduction-811)

"This course will explore fundamental moral issues that arise in medicine, health, and biotechnology. Get behind the headlines—and polarized debates—and join others who want to think deeply and openly about these problems. Some are as old as life itself: the vulnerability of illness, the fact of death. Some are new, brought on by a dizzying pace of technology that can unsettle our core ideas about human nature and our place in the world. And nearly all intersect with issues of racial and gender equality, as well as policies affecting the world's most vulnerable populations."

Commentary. With every major medical center now employing bioethicists and many supporting departments devoted to the subject, there is no debating the importance of the subject to researchers in general. That applies to information technologists in particular when it comes to privacy issues, as well as to anyone working in any aspect of human genetics and genomics. This is a very refined production by an all-star cast of faculty, hitting all the expected topics as well as some more unusual issues, such as medical tourism and climate justice.

Textbook. A fulsome collection of readings is provided online, including excerpts from books by various of the instructors.

Alternatives. The course Clinical Trials in this Department has a generous dose of lectures on ethical aspects of human studies. The NIH offers a comprehensive short course on "Ethical and Regulatory Aspects of Clinical Research" (http://clinicalcenter.nih.gov/podcast/bioethics_eracr.html).

Going Further. In slightly different veins, Coursera has "Technology and Ethics" by Robert Bailey of Ohio State University (https://www.coursera.org/course/techethics) and "Neuroethics" by Jonathan D. Moreno of the University of Pennsylvania (https://www.coursera.org/course/neuroethics). See the general Ethics course in the Humanities Department for a less specialized treatment of the subject.

Healthcare Policy

John E. McDonough, Harvard School of Public Health "United States Health Policy" (edX, Spring 2014: https://www.edx.org/course/harvardx/harvardx-ph210x-united-states-health-1407)

"Featuring some of the nation's foremost teachers and thought leaders, this course provides students with a basic and thorough understanding of the U.S. health care system focusing on access, quality of care, and costs. Students will learn how the system is structured, how care is organized, delivered, and financed, and how the Affordable Care Act will influence the future of the system. Students will understand the U.S. health policy making structure process at the federal, state, and local levels."

Commentary. The events surrounding the initial rollout of the U.S. healthcare.gov web site under Obamacare should leave no doubt as to the importance of informatics to healthcare policy. The instructor worked on the writing and passage of the Affordable Care Act and was a member of the Massachusetts House of Representatives where he co-chaired the Joint Committee on Health Care.

Textbook. *Inside National Health Reform* by the instructor is recommended [95]. Portions are made available during the course.

Alternatives. Coursera has "Understanding and Improving the US Healthcare System" by Matthew Davis of the University of Michigan (https://www.coursera.org/course/ushealthcare) and "Health Policy and the Affordable Care Act" by Ezekiel Emanuel of the University of Pennsylvania, one of the illustrious Emanuel brothers (https://www.coursera.org/course/healthpolicy).

Healthcare Entrepreneurship

Regina Herzlinger, Margo Seltzer, and Kevin Schulman, Harvard Business School "Innovating in Health Care" (edX, Spring 2014: https://www.edx.org/course/harvardx/harvardx-bus5-1x-innovating-health-care-1405)

"The course focuses on evaluating and crafting business models that attain alignment between an entrepreneurial health care venture and the six factors that critically shape new health care ventures - Financing, Structure, Public Policy, Consumers, Technology, and Accountability. Innovating in Health Care discusses the impact of these factors on business models for three different kinds of innovations: consumer-focused, technology-driven, and integrations which create scale."

Commentary. Opportunities for healthcare startups outside the therapeutic realm tend to involve the web and some form of informatics. This course is offered both as a regular MOOC and in a limited version that admits 100 teams of 6 people who then develop a health care business idea in depth.

Textbook. The course makes use of Harvard business case studies (see the description for the Business course in the Humanities Department), several of which are provided free to students in the course and others of which are purchasable. Some are optional and some are "strongly recommended."

Personal Note. The author has watched a healthcare web startup founded by cousins of his a decade ago grow from 3 to 70 employees, and it is a fascinating process to witness. See PatientsLikeMe.com for an interesting mix of technology, social networking, analytics, interface design, academic research, and a unique business model. (The author has no financial stake in the company.)

Alternatives. Coursera also has a course on "Healthcare Innovation and Entrepreneurship" by Marilyn Lombardi and Bob Barnes of Duke University, which involves a more conventional lecture format (https://www.coursera.org/course/healthcareinnovation), but which also tends slightly to the platitudinous. See also Entrepreneurial Skills in the Humanities Department.

Current Topics in Genomic Medicine

Various Speakers, U.S. National Institutes of Health and The Johns Hopkins University "Genomics in Medicine Lecture Series" (GenomeTV/YouTube, 2011-2014: http://www.genome.gov/27546022, http://www.genome.gov/27549874, and http://www.genome.gov/27553517)

"This ... lecture series by top experts in genomics will enhance health-care professionals' understanding of the intersection between genomics and medicine."

Commentary. These twenty lectures together provide a very comprehensive overview of current research and practice in genomic medicine, with the most recent series focusing on cancer. The speakers are drawn from various institutes of the National Institutes of Health as well as the Johns Hopkins School of Medicine. Slides are made available for most of the lectures.

Drug Discovery Seminars

(See seminars at http://www.youtube.com/playlist?list=PL9s5HIfOWP3OHGrX6wEkvIHLGyDK8-KSl)

Translational Sciences Seminars

(See seminars at http://www.youtube.com/playlist?list=PL9s5HIfOWP3NdWPjoSPxdSJhlOLVTfIHC)

Humanities Department

If the inclusion of this department comes as a surprise, consider that most undergraduate programs have some sort of distribution requirement that mandates coursework outside science and engineering. Usually the rationale is that educational institutions should produce well-rounded individuals, but arguments can be made that are more relevant to scientific training, for instance that exposure to philosophy improves reasoning skills, exposure to literature improves reading and writing skills, exposure to history helps place science in societal context, and so forth.

Moreover, perhaps the most dynamic enterprise in the humanities today sits at its intersection with computation, in what is termed "Digital Humanities" (DH). Salient DH concerns include curation and data mining, both of which are vitally important in bioinformatics as well. Recognizing this, the U.S. National Institutes of Health and National Endowment for the Humanities, along with the Research Councils U.K., recently sponsored and participated in a symposium at the University of Maryland Institute for Technology in the Humanities called "Shared Horizons: Data, Biomedicine, and the Digital Humanities" to explore the interface; the author had the privilege of delivering the keynote address, which was entitled "With a Wild Surmise: Intimations of Computational Biology in Keats, Carroll, and Joyce."

We use an exceedingly broad definition of humanities for this virtual department, including essentially any subject outside the scope of "hard" science and engineering, and also courses explicitly designed to improve students' skills in writing, speaking, and the like.

Introduction to Philosophy 💻✓

Caspar Hare, Massachusetts Institute of Technology "Introduction to Philosophy: God, Knowledge and Consciousness" (edX, Fall 2013: https://www.edx.org/course/mitx/mitx-24-00x-introduction-philosophy-god-888)

> *"This course has two goals. The first is to introduce you to the things that philosophers think about. We will look at some perennial philosophical problems: Is there a God? What is knowledge, and how do we get it? What is the place of our consciousness in the physical world? Do we have free will? How do we persist over time, as our bodily and psychological traits change? The second goal is to get you thinking philosophically yourself. This will help you develop your critical reasoning and argumentative skills more generally. Along the way we will draw from late, great classical authors and influential contemporary figures."*

Commentary. The course provides a good taste of the methodology of modern philosophy, particularly emphasizing logical rigor. The segments involving class discussions, both real and virtual, are especially significant for this course.

Evaluation.

Course Level	*Hours per Week*	*Course Grade*				*Student Grade*	*Curve*
		Lectures	*Homework*	*Assessment*	*Overall*		*Pass/"A"*
Introductory	6	95	90	93	94	96	66/—

Personal Note. The author took an early classroom version of this course and was inspired to do a second undergraduate degree in philosophy. While that would be overkill for committed computational biologists, it did prove to be good preparation for elements of computer science such as logic, of computational biology such as ontology, and of systems biology such as emergence [96].

Textbook. All necessary readings are provided. The philosophical version of open access includes the journals *Philosopher's Imprint* from the University of Michigan (http://www.philosophersimprint.org) and *Notre Dame Philosophical Reviews* (http://ndpr.nd.edu), as well as the *Stanford Encyclopedia of Philosophy* (http://plato.stanford.edu).

Alternatives. A team of faculty from the University of Edinburgh offer a competing "Introduction to Philosophy" on Coursera (https://www.coursera.org/course/introphil). It is elaborately produced and of interest for its (brief) coverage of the philosophy of science, but compared to the course above it treats more topics with less depth and less attention to methodology. A more traditional historical introduction to philosophy can be had in non-MOOC form from Peter Millican of Oxford University on iTunes U (https://itunes.apple.com/us/itunes-u/general-philosophy/id381701367). Oxford also has a very short introductory course "Philosophy for Beginners" by Marianne Talbot, also on iTunes U (https://itunes.apple.com/us/itunes-u/philosophy-for-beginners/id381704133).

Going Further. See the courses immediately below for some of the standard subtopics of the field. In addition, Coursera has several more specialized courses of note. Michael Roth, the president of Wesleyan University, is also a noted philosopher and teaches "The Modern and the Postmodern" with considerable charisma (https://www.coursera.org/course/modernpostmodern). Jon Stewart of the University of Copenhagen offers "Søren Kierkegaard—Subjectivity, Irony and the Crisis of Modernity" (https://www.coursera.org/course/kierkegaard), which is not only beautifully taught but is recorded on location at many historic and picturesque sites around Denmark. (Note that to study Kierkegaard is to study Socrates, so the course is not as narrow as it may sound.)

Logic 💻

(See listing in Mathematics Department.)

Ontology

Barry Smith, State University of New York, Buffalo "An Introduction to Ontology: From Aristotle to the Universal Core" (Buffalo Ontology Site, 2009: http://ontology.buffalo.edu/smith/IntroOntology_Course.html)

> *"This course is designed to be of interest to both philosophers and those with a background in computer and information science. No prior knowledge of ontology is presupposed."*

Commentary. Ontology was a keystone of metaphysics long before it became associated with computer science and bioinformatics. The instructor is a philosopher by training, but has become involved in biomedical ontologies and other applications. This short non-MOOC course provides the philosophical grounding, then discusses ontology in a computer science context, including the semantic web. (If you have trouble with the WMV files, try opening them in VLC, obtainable from http://www.videolan.org).

Going Further. The instructor has several other video tutorials online, including more on biomedical ontologies (http://ncorwiki.buffalo.edu/index.php/Barry_Smith:_Video_Introductions_to_Ontology).

Ethics 💻

Michael Sandel, Harvard ER22.1x "Justice" (EdX, Spring 2014: http://www.justiceharvard.org or https://www.edx.org/course/harvardx/harvardx-er22-1x-justice-1408)

> *"Justice is a critical analysis of classical and contemporary theories of justice, including discussion of present-day applications. Topics include affirmative action, income distribution, same-sex marriage, the*

role of markets, debates about rights (human rights and property rights), arguments for and against equality, dilemmas of loyalty in public and private life."

Commentary. This course is a carryover from the previous catalog, now available on the edX platform but offered for self-study rather than being scheduled. While there are more specialized ethics courses listed under Alternatives and Going Further, this storied Harvard course provides a good general introduction with very high production values.

Textbook. The instructor's book *Justice: What's the Right Thing to Do?* [97] is very reasonably priced, but there is no shortage of alternatives. For coverage in depth, see the Stanford Encyclopedia of Philosophy (http://plato.stanford.edu) entries for the three main moral theories: consequentialism, deontological ethics, and virtue ethics.

Alternatives. Princeton's Peter Singer, who is famous for his views on animal rights, has an entry on Coursera entitled "Practical Ethics" (https://www.coursera.org/course/practicalethics). Oxford has a short non-MOOC tutorial called "A Romp through Ethics for Complete Beginners," taught by Marianne Talbot with more focus on traditional moral philosophy (http://podcasts.ox.ac.uk/series/romp-through-ethics-complete-beginners). For a psychologist's take on ethics, see "Moralities of Everyday Life" by Yale's Paul Bloom on Coursera (https://www.coursera.org/course/moralities). For a business perspective, see "Unethical Decision Making in Organizations" by Guido Palazzo and Ulrich Hoffrage of the University of Lausanne, also on Coursera (https://www.coursera.org/course/unethicaldecision). See the previous catalog for still more possibilities.

Going Further. See the Biomedical Ethics course in the Translational Sciences Department for specialized application of these general principles.

Philosophy of Mind

Marianne Talbot, Oxford University "A Romp through the Philosophy of Mind" (iTunes U: https://itunes.apple.com/us/itunes-u/romp-through-philosophy-mind/id503111374)

"These days many people believe the mind simply is the brain. Descartes would have disagreed profoundly. He recommended a dualism of substance. Modern philosophers are again finding various forms of dualism attractive because the problems with physicalism are so intractable. One such problem is whether the mind, like the brain, is located in space (specifically inside the head). But does philosophy have anything sensible to say about the mind? Surely today it is scientists we should be listening to? Come and find out why this is—and always will be—false."

Commentary. Philosophy of mind (not to be confused with epistemology, which is the philosophy of knowledge, covered in the Introduction to Philosophy above) has always flirted with the neuroscience of consciousness, and vice versa. This short, somewhat tendentious course may thus prove a useful counterpoint to the course on Consciousness in the Neurosciences Department.

Textbook. For under $10, *The Mystery of Consciousness* by John Searle [98], which is a series of book reviews and responses by the authors, may be the best overview of the area despite being dated. One of the most entertaining ways to absorb the subject is through the novel *Thinks...* by the prolific British author David Lodge [99], who has also written a related work of literary criticism, *Consciousness and the Novel* [100].

Personal Note. The author's undergraduate advisor was Ned Block, now at New York University, who subsequently became famous for his work on the philosophy of mind, consciousness, and in particular his arguments against the validity of the Turing Test for artificial intelligence. See a rather quirky interview with him at http://vimeo.com/58254376 for a brief explication of the mind-body problem

together with a music video on the same topic by a rock band called The Amygdaloids, the members of which are all scientists.

Alternatives. A more extensive (also non-MOOC) course that spends more time on the neuroscience of consciousness is "The Nature of Mind" by John Joseph Campbell of the University of California, Berkeley (http://webcast.berkeley.edu/playlist#c,d,Philosophy,-XXv-cvA_iAX6VIQl_dauu368BQFSpX0 or http://www.youtube.com/view_play_list?p=-XXv-cvA_iAX6VIQl_dauu368BQFSpX0).

Going Further. Marvin Minsky, a pioneer of artificial intelligence at the Massachusetts Institute of Technology, offers a graduate seminar on MIT OpenCourseWare called "The Society of Mind" that mixes psychology and computer science in a comprehensive effort to explain how minds are made from collections of simpler processes (http://ocw.mit.edu/courses/electrical-engineering-and-computer-science/6-868j-the-society-of-mind-fall-2011). The upper-level "Philosophy of Mind" by Berkeley's John Searle (http://itunes.apple.com/WebObjects/MZStore.woa/wa/viewPodcast?id=701381047) is only available as audio on iTunes U, but is nonetheless worthwhile because the instructor is perhaps the most eminent philosopher in the field (though he is often at odds with proponents of Artificial Intelligence).

Philosophy and Science

Michela Massimi, Alasdair Richmond, Suilin Lavelle, David Carmel, Mark Sprevak, Duncan Pritchard, Andy Clark, John Peacock, Barbara Webb, Kenny Smith and Peggy Series, The University of Edinburgh "Philosophy and the Sciences" (Coursera, Fall 2014: https://www.coursera.org/course/philsci)

> *"Scientific research across both the physical sciences and the cognitive sciences has raised pressing questions for philosophers. The goal of this course is to introduce you to some of the main areas and topics at the key juncture between philosophy and the sciences… Areas you'll learn about will include: Philosophy of cosmology, where we'll consider questions about the origin and evolution of our universe, the nature of dark energy and dark matter and the role of anthropic reasoning in the explanation of our universe… Philosophy of psychology, among whose issues we will cover the evolution of the human mind and the nature of consciousness… Philosophy of neurosciences, where we'll consider the nature of human cognition and the relation between mind, machines, and the environment."*

Commentary. This course is of interest for its treatment of certain specific scientific fields, obviously, but it should be noted that what is usually termed the philosophy of science actually examines science as a discipline, with its scope including the general nature of science and the scientific method, the character of scientific proof, the epistemological status of scientific theories and explanations, and the like. Unfortunately no MOOCs appear to be extant in the philosophy of science *per se*, though it is one of the major subfields of philosophy today.

Textbook. Extensive lecture notes on the philosophy of science have been posted by Lyle Zynda of Iowa State University (http://www.soc.iastate.edu/sapp/phil_sci_lecture00.html). There are many textbooks on the subject, easily discovered.

Alternatives. Although there are no academic video courses available on philosophy of science, as noted above, there is a lucid and fairly comprehensive series on YouTube entitled "An Introduction to the History and Philosophy of Science" (http://www.youtube.com/playlist?list=PL67E2553770A6E39E). However, the lecturer is identified only by the tag "SisyphusRedeemed," and he simply describes himself as a "professional philosopher," so buyer beware. James Paradis of the Massachusetts Institute of Technology puts evolution in a philosophical and literary context in his course "Darwin and Design" (http://ocw.mit.edu/courses/literature/21l-448j-darwin-and-design-fall-2010/index.htm).

Philosophy and Mathematics 💻✓

Hannes Leitgeb and Stephan Hartmann, Ludwig-Maximilians-Universität München "Introduction to Mathematical Philosophy" (Coursera, Spring 2014: https://www.coursera.org/course/mathphil)

> *"Infinity (Zeno's Paradox, Galileo's Paradox, very basic set theory, infinite sets)… Truth (Tarski's theory of truth, recursive definitions, complete induction over sentences, Liar Paradox)… Rational Belief (propositions as sets of possible worlds, rational all-or-nothing belief, rational degrees of belief, bets, Lottery Paradox)… If-then (indicative vs subjunctive conditionals, conditionals in mathematics, conditional rational degrees of belief, beliefs in conditionals vs conditional beliefs)… Confirmation (the underdetermination thesis, the Monty Hall Problem, Bayesian confirmation theory)… Decision (decision making under risk, maximizing expected utility, von Neumann Morgenstern axioms and representation theorem, Allais Paradox, Ellsberg Paradox)… Voting (Condorcet Paradox, Arrow's Theorem, Condorcet Jury Theorem, Judgment Aggregation)… Quantum Logic and Probability (statistical correlations, the CHSH inequality, Boolean and non-Boolean algebras, violation of distributivity)."*

Commentary. Note that this is not the philosophy of mathematics, but rather the use of mathematics to conduct philosophical investigation. It covers some of the most fundamental underpinnings of logic and probability, through the lens of philosophy.

Evaluation.

Course Level	*Hours per Week*	*Course Grade*				*Student Grade*	*Curve*
		Lectures	*Homework*	*Assessment*	*Overall*		*Pass/"A"*
Intermediate	8	94	94	90	91	95.8	70/—

Textbook. The instructors recommend several books at the end of the course, but also provide a useful collection of online lecture notes from a variety of sources (http://www.mcmp.philosophie.uni-muenchen.de/students/math/index.html).

Going Further. From the same instititution, a series of advanced seminars on related topics is available on iTunes U (https://itunes.apple.com/us/itunes-u/mcmp-philosophy-of-science/id654726625).

Linguistics

Jürgen Handke, Philipps-Universität Marburg "Linguistic Fundamentals," "Speech Science," and "The Nature of Meaning" (The Virtual Linguistics Campus: http://linguistics.online.uni-marburg.de or https://www.youtube.com/playlist?list=PLRIMXVU7SGRK7aWd4TtgUVqGOaYqflR0g, https://www.youtube.com/playlist?list=PLRIMXVU7SGRJwPnGUNly2eyISuF5iFwCv, and https://www.youtube.com/playlist?list=PLRIMXVU7SGRK7aWd4TtgUVqGOaYqflR0g)

> [Linguistic Fundamentals] *"… provides an overview of the core areas of linguistics from phonetics to pragmatics. It serves as the fundamental introduction to the field using examples from English as well as other languages."* [Speech Science] *"… discusses all aspects of human speech: speech production, speech perception and the physics of speech (including the computer-based analysis of speech sounds)."* [The Nature of Meaning] *"… discusses all aspects of meaning: from word meaning, via sentence meaning (including propositional and predicate logic) to the meaning of utterances. Furthermore, it includes aspects such as ambiguity vs. vagueness and historical aspects of word meaning."*

Commentary. This home-brew MOOC from the University of Marburg includes three short courses that together comprise a nice overview of the field of linguistics. One can either register at the Virtual Linguistics Campus for participation in the MOOC, or use the YouTube links to audit the lectures. This is

good preparation for the Natural Language Processing course in the Computer Science Department, and in turn for a deeper understanding of issues in text mining.

Personal Note. While a member of the genetics faculty at the University of Pennsylvania, the author was also affiliated with the cross-departmental Institute for Research in Cognitive Science (http://www.ircs.upenn.edu) where computer scientists, linguists, logicians, philosophers, psychologists, and neuroscientists have productively communed for decades. It may be argued that cognitive scientists pioneered the same sort of cross-fertilization between engineering and scientific fields that computational biologists strive for, but notably also incorporated humanities into the mix.

Textbook. The instructor has an extensive list of suggested books (http://linguistics.online.uni-marburg.de). Marcus Kracht, now at the University of Beilefeld, has posted very polished lecture notes for a linguistics course he taught at the University of California, Los Angeles (http://wwwhomes.uni-bielefeld.de/mkracht/html/ling-intro.pdf). (See also his notes under the Natural Language Processing course in the Computer Science Department above.)

Going Further. The same site also offers an advanced course by the same instructor in "The Structure of English" (https://www.youtube.com/playlist?list=PLRIMXVU7SGRLxULxRrVjWbn-XwaUjgz-B) as well as a number of other resources. Martin Hilpert of the University of Neuchatel teaches "An Introduction to English Linguistics" at about the same level (http://members.unine.ch/martin.hilpert/intro.html).

Literature

Eric Rabkin, University of Michigan "Fantasy and Science Fiction: The Human Mind, Our Modern World" (Coursera, Winter 2014: https://www.coursera.org/course/fantasysf)

> *"From a practical viewpoint, of all the fictional forms that fantasy takes, science fiction, from Frankenstein to Avatar, is the most important in our modern world because it is the only kind that explicitly recognizes the profound ways in which science and technology, those key products of the human mind, shape not only our world but our very hopes and fears. This course will explore Fantasy in general and Science Fiction in specific both as art and as insights into ourselves and our world."*

Commentary. Nothing improves one's reading and writing skills like a good literature course, where MOOCs tend to have demanding reading lists and many peer-reviewed writing assignments. Of all the possibilities, the genre of fantasy and science fiction is the most obvious suggestion for computational biologists. For one thing, scientists and engineers probably read such books disproportionately already. For another, fantasy and science fiction would seem to be a way to exercise imaginative powers relevant to the field.

Evaluation.

Course Level	Hours per Week	Course Grade				Student Grade	Curve
		Lectures	Homework	Assessment	Overall		Pass/"A"
Introductory	12	92	95	93	92	82.1	?/?

Textbook. An extensive reading list is provided, much of which is in the public domain and can be downloaded from Project Gutenberg (http://www.gutenberg.org). Among these are several classics of biological interest, such as Mary Shelley's *Frankenstein*, H.G. Wells' *The Island of Dr. Moreau*, and stories by Nathaniel Hawthorne including "Rappacini's Daughter" and "Dr. Heidegger's Experiment." (Note: Project Gutenberg offers books in formats including HTML, EPUB, Kindle, and plain text UTF-8; the author has used the latter extensively to download corpuses for text mining purposes.)

Personal Note. While it may seem obvious that science fiction should be the closest approach that literature makes to science, the author is bound to acknowledge that his first journal publication was a

commentary in *Technology Review* on the 29th World Science Fiction Convention that was skeptical about the relevance of the modern genre to real-world science [101]. Those with similar doubts may want to consider the alternatives below.

Alternatives. Fantasy and science fiction is certainly not to everyone's taste, and there are many MOOC alternatives. For those who feel they spend way too much time in the lab or at the computer, "The Fiction of Relationship" by Arnold Weinstein of Brown University is excellent, and scientists may appreciate especially the works by Kafka and Borges (https://www.coursera.org/course/relationship). Fans of historical fiction should look at "Plagues, Witches, and War: The Worlds of Historical Fiction" as taught by Bruce Holsinger of the University of Virginia (https://www.coursera.org/course/hisfiction), which includes interactions with several working authors and focuses on research techniques. Those who prefer verse may like "The Art of Poetry" by Boston University's Robert Pinsky, a former U.S. Poet Laureate and "the only member of the American Academy of Arts and Letters to have appeared on both *The Simpsons* and *The Colbert Report*" (https://www.edx.org/course/bux/bux-arpo222x-art-poetry-1565). For a bit of the Bard, try "Shakespeare: On the Page and in Performance" by Yu Jin Ko of Wellesley College (https://www.edx.org/course/wellesleyx/wellesleyx-eng112x-shakespeare-page-877), and perhaps a recent book by Dan Falk, *The Science of Shakespeare,* which finds intriguing connections between the playwright and the Scientific Revolution [102]. Finally, if you have a taste for the classics, consider "The Ancient Greek Hero" by the eminent Gregory Nagy of Harvard (https://www.edx.org/course/harvardx/harvardx-cb22-1x-ancient-greek-hero-1047), a truly epic overview of ancient literature, and especially of Homer, whose oeuvre was recently dated to 710-760 BCE by molecular phylogeneticists using a Markov chain Monte Carlo procedure on his vocabulary [103].

Personal Note. As an undergraduate, the author took several courses in the classics including one taught by Prof. Nagy when he was a newly-minted Assistant Professor at Harvard. In taking his edX course now it is gratifying to see that this distinguished teacher's passion for the subject is undiminished, and further that he has become "a strong proponent of the use of technology in teaching" (http://chs.harvard.edu/wa/pageR?tn=ArticleWrapper&bdc=12&mn=1234). Later, as a graduate student in biology, the author stole away from the lab to audit a course on "Yeats, Pound, and Eliot" by the famous literary critic Hugh Kenner, who also had a penchant for crossing the arts/sciences divide; besides his extensive literary output, Kenner published a book on geodesic math and a user's guide to an early personal computer (http://www.nytimes.com/2003/11/25/books/25KENN.html).

Going Further. The edX course "Representations of HIV/AIDS" by Davidson College's Ann Fox (who teaches literature) and David Wessner (a biologist) should be a marvelous opportunity to explore the possibilities of radically cross-disciplinary studies, in the particular context of the AIDS epidemic (https://www.edx.org/course/davidsonx/davidsonx-d002-representations-hivaids-1383).

History

Yuval Noah Harari, The Hebrew University of Jerusalem "A Brief History of Humankind" (Coursera, Fall 2013: https://www.coursera.org/course/humankind)

> *"The course surveys the entire length of human history, from the evolution of various human species in the Stone Age up to the political and technological revolutions of the twenty-first century."*

Commentary. History might seem far removed from computational biology, but there are recent trends in historical scholarship that may narrow the gap. One is an increasing use of quantitative methods, and in particular a dynamical systems approach to historical change termed "cliodynamics" [104]. Another is what is called "Big History," which studies the entire scope of the history of the world and indeed of the universe from the Big Bang onward, in a highly multidisciplinary fashion. The evolution of life is an

important component, and this course limits itself to human history in particular. It may thus be seen as background for paleoanthropology with particular emphasis on the development and transmission of culture, and the course also examines the important place of science and technology in the context of history (particularly as it relates to the scientific revolution and to imperialism). Biologists may object to the speculative nature of the discourse, but it is undeniably thought provoking, and after all it is very much in the style of the biologist Jared Diamond's runaway bestseller *Guns, Germs, and Steel* [105].

Evaluation.

Course Level	Hours per Week	Course Grade				Student Grade	Curve
		Lectures	Homework	Assessment	Overall		Pass/"A"
Introductory	5	89	90	85	88	100.0	70/—

Textbook. None is required, though the course is based on a book by the instructor that is still in the process of being translated into English. There are countless world history textbooks available, which can readily be bought used.

Alternatives. If you prefer a more conventional approach, Coursera has "The Modern World: Global History since 1760" by the University of Virginia's Philip Zelikow, who is both distinguished (he directed the 9/11 Commission) and highly ingratiating (https://www.coursera.org/course/modernworld). For a slightly wider scope there is Coursera's "A History of the World since 1300" by Jeremy Adelman of Princeton University (https://www.coursera.org/course/wh1300). Annenberg Learner offers two contrasting approaches: one is a global history treatment like those above called "Bridging World History" (http://www.learner.org/resources/series197.html), and the other is "The Western Tradition" by Eugen Weber of the University of California, Los Angeles, which at a quarter-century after its recording provides a glimpse of a traditional, pre-globalization syllabus (with the same title as the author's freshman history course) (http://www.learner.org/resources/series58.html).

Archaeology

Sue Alcock, Brown University "Archaeology's Dirty Little Secrets" (Coursera, Winter 2014: https://www.coursera.org/course/secrets)

> *"In this class, we will ask and answer a series of questions about the role and practice of archaeology in the world today. If archaeologists are trained to investigate the past, what is left for us to study? Who gets to be an archaeologist? How and why do archaeologists hunt for 'treasures', and what do we do once we've discovered them? What can we know, and not know, about people in the past?"*

Commentary. This is another field that has taken a sharp turn toward the digital in recent years [106]. In fact, some of the most exciting new archaeology is being done from space [107], thrusting the field decisively into the realm of big data. Even before these developments, a subfield called evolutionary archaeology had been adopting Darwinian principles in its methodology [108]. This entertaining and unpretentious course samples many of the ways archaeologists work today, and features exercises such as learning to write cuneiform in modeling clay and locating archaeological sites with Google Earth.

Textbook. Readings are supplied, but to go further the instructor recommends *Archaeology: Theories, Methods, and Practice* by the renowned Colin Renfrew and Paul Bahn [109], earlier editions of which can be gotten used at a much lower price.

Alternatives. An anthropological take on the subject is offered by Annenberg Learner in "Out of the Past," produced with Pennsylvania State University (http://www.learner.org/resources/series45.html).

Architecture

Mark Jarzombek, Massachusetts Institute of Technology 4.605x "A Global History of Architecture—Part 1" (edX, Fall 2013: https://www.edx.org/course/mitx/mitx-4-605x-global-history-architecture-884)

> *"This course will examine architecture through time, beginning with First Societies and extending to the 15th century. Though the course is chronological, it is not intended as a linear narrative, but rather aims to provide a more global view, by focusing on different architectural 'moments.' The lectures will give students the appropriate grounding for understanding a range of buildings and contexts."*

Commentary. Like both biological sciences and engineering, the history of architecture deals with complex forms that evolve over time and for which the interrelationship between structure and function is a key concern. Thus we speak of computer architectures and of the architectures of biological structures like cells, tissues, and organs. Aside from the intrinsic interest of the subject, students may find an elective in this area stimulates analogical thinking in productive ways. That has certainly been the case for prominent evolutionary biologists like Stephen Jay Gould and Richard Lewontin [110-111].

Evaluation.

Course Level	Hours per Week	Course Grade				Student Grade	Curve
		Lectures	Homework	Assessment	Overall		Pass/"A"
Introductory	6	92	89	93	90	95	80/—

Textbook. *A Global History of Architecture* by the instructor with Francis Ching and Vikramaditya Prakash [112] is a fantastic resource, though moderately expensive. It is helpful for the course in that the video images are all too fleeting and the handouts provided are terse.

Alternatives. While the course above provides an anthropological perspective, the non-MOOC "History of Architecture I" by Jacqueline Gargus of the Ohio State University is more oriented to design and aesthetics (https://itunes.apple.com/us/course/history-of-architecture-i/id570008367).

Going Further. Coursera offers a wonderful course on "Roman Architecture" by Diana Kleiner of Yale University (https://www.coursera.org/course/romanarchitecture). The instructor was the Founding Director of Open Yale Courses.

Art

Jeannene Przyblyski, California Institute of the Arts "Live!: A History of Art for Artists, Animators and Gamers" (Coursera, Winter 2014: https://www.coursera.org/course/livearthistory)

> *"Explore art history from the artist's perspective. Learn how contemporary artists, animators and gamers work from the art of the past as part of their creative process, while building your own skills in visual analysis and creative and critical thinking."*

Commentary. There are a surprising number of connections between art, computing, and biology. From a scientific perspective, neuroscientists and evolutionary biologists are increasingly interested in art and the brain [113]. As noted under the Paleoanthropology course in the Evolutionary Biology Department, artists are also important in the reconstruction of extinct species. Since the legendary work of the scientific illustrator Irving Geis, practitioners of biomolecular graphics have recognized the importance of aesthetics to their work [50], and now molecular animations have become important tools in research and teaching [51]. The latter fact lends particular interest to this course, geared as it is toward animators and gamers. In that regard, note the importance of gaming to recent discoveries in protein structure and design coming from the University of Washington laboratory of David Baker together with an online game-playing community [114-115].

Textbook. None is required. Art, and art books, are everywhere, of course.

Alternatives. Annenberg Learner offers two short video courses: "Art of the Western World" (http://www.learner.org/resources/series1.html) and "Art through Time: A Global View" (http://www.learner.org/resources/series211.html). For those who might be tempted to try their own hands at creating art, Coursera has "Introduction to Art: Concepts and Techniques" by Anna Divinsky of the Pennsylvania State University (https://www.coursera.org/course/art).

Going Further. For a sense of the uses of animation in biology, watch the short clip from iBioMagazine entitled "Animating Cell Biology" by Janet Iwasa, then at Harvard and now at the University of Utah (http://www.youtube.com/watch?v=68MnuydU1jk).

Music

Michael Edwards, Zack Moir, and Richard Worth, University of Edinburgh "Fundamentals of Music Theory" (Coursera, Summer 2014: https://www.coursera.org/course/musictheory)

> *"This course will introduce students to the theory of music, providing them with the skills needed to read and write Western music notation, as well as to understand, analyse, and listen informedly. It will cover material such as pitches and scales, intervals, clefs, rhythm, form, meter, phrases and cadences, and basic harmony."*

Commentary. Music hath charms that are well known, but there are many other reasons that it might interest a computational biologist. Neuroscientists have studied its effects on the brain (see Going Further, below). The famous evolutionary biologist and cognitive scientist W. Tecumseh Fitch has combined those two fields in his study of music [116]. Several bioinformatics groups have worked on birdsong, in terms of sequence analysis [117], syntactic analysis [118], and the transcriptomics of "musical" brain regions [119] as well as of the FoxP2 gene thought also to be related to human language [120]. There are many music appreciation MOOCs (see Alternatives, below), but this music theory course should appeal to the computationally minded.

Textbook. Though none is required, *Tonal Harmony* by Stefan Kostka and Dorothy Payne [121], a standard text, is recommended. A less expensive (free) alternative is the much briefer OpenStax book *Introduction to Music Theory* by Catherine Schmidt-Jones (http://cnx.org/content/col10208/latest).

Alternatives. Other music courses on Coursera include "From the Repertoire: Western Music History through Performance" by Jonathan Coopersmith and David Ludwig of the Curtis Institute of Music (https://www.coursera.org/course/musichistoryperforms), "Introduction to Classical Music" by Kevin Korsyn of the University of Michigan (https://www.coursera.org/course/introtoclassical), "Jazz Appreciation" by Jeffrey Hellmer of the University of Texas, Austin (which makes heavy use of a novel adaptive learning technology) (https://www.edx.org/course/utaustinx/utaustinx-ut-8-01x-jazz-appreciation-1149), and "Listening to World Music" by Carol Muller of the University of Pennsylvania (https://www.coursera.org/course/worldmusic), among others. Annenberg Learner offers "Exploring the World of Music" (http://www.learner.org/resources/series105.html).

Going Further. For the neurosciences perspective, the Stanford Institute for Creativity and the Arts has an annual Music and the Brain Symposium, one edition of which is available on iTunes U (https://itunes.apple.com/us/itunes-u/music-and-the-brain-symposium/id480026138). The U.S. Library of Congress has a similar series of webcasts (https://itunes.apple.com/us/itunes-u/music-and-the-brain-webcasts/id386017685).

Economics

Antonio Rangel, California Institute of Technology "Principles of Economics for Scientists" (Coursera, TBA: https://www.coursera.org/course/econ1scientists)

> *"The impact of economic forces in our lives is sizable and pervasive. For this reason, it is impossible to understand the social and economic forces shaping our lives without a good understanding of basic economic principles. This course provides a quantitative and model-based introduction to such principles, and teaches how to apply them to make sense of a wide range of real world problems."*

Commentary. This course makes the already dismal science even more rigorously quantitative, as befits CalTech, and it seems sure to appeal to computational biologists if any economics course could. Besides the pragmatic benefit of knowledge of this topic in a world where science costs money, principles of economics may also prove useful as tools. For example, a recent study published in this journal borrowed utility functions from economics to study motor control [122], and another publication used economic concepts to model the evolution of mutualistic symbioses [123].

Textbook. None is required. Economics textbooks abound, of course, and are generally not very economical, but one that is Creative Commons-licensed and recommended as an alternative for the MIT OpenCourseware course is *Principles of Microeconomics* by Libby Rittenberg and Timothy Tregarthen (http://ocw.mit.edu/ans7870/14/14.01SC/MIT14_01SCF11_rttext.pdf).

Alternatives. The instructor appears to have shifted his loyalties to edX, teaching "Principles of Economics with Calculus" in Winter, 2014 (https://www.edx.org/course/caltechx/caltechx-ec1011x-principles-economics-1286), so if the course above does not reappear on Coursera look for it on edX. There are numerous other economics courses available on Coursera and elsewhere, too many to list but easy to find.

Business

Brian J. Bushee, The Wharton School of the University of Pennsylvania "An Introduction to Financial Accounting" (Coursera, Fall 2013: https://www.coursera.org/course/accounting)

> *"This course will improve your fluency in financial accounting, the language of business. You will learn how to read, understand, and analyze most of the information provided by companies in their financial statements. These skills will help you make more informed decisions using financial information."*

Christian Terwiesch, The Wharton School of the University of Pennsylvania "An Introduction to Operations Management" (Coursera, Spring 2014: https://www.coursera.org/course/operations)

> *"This course will teach you how to analyze and improve business processes, be it in services or in manufacturing. You will learn how to improve productivity, how to provide more choice to customers, how to reduce response times, and how to improve quality."*

David Bell, Peter Fadar, and Barbara E. Kahn, The Wharton School of the University of Pennsylvania "An Introduction to Marketing" (Coursera, Fall 2013: https://www.coursera.org/course/marketing)

> *"This Wharton course will teach the fundamentals of marketing by getting to the root of customer decision making. The course will focus on branding strategies, customer centricity and new market entry."*

Franklin Allen, The Wharton School of the University of Pennsylvania "An Introduction to Corporate Finance" (Coursera, Fall 2013: https://www.coursera.org/course/finance)

> *"This course will provide a market-oriented framework for analyzing the major types of financial decisions made by corporations. Lectures and readings will provide an introduction to present value*

techniques, capital budgeting principles, asset valuation, the operation and efficiency of financial markets, the financial decisions of firms, and derivatives."

Commentary. Wharton, which is consistently ranked among the very top business schools in the world, has put a core curriculum online as their "Foundation Series." Anyone who thinks he or she might one day work in industry, manage a sizeable lab, do some consulting, or spin off a company could benefit hugely from training in business.

Personal Note. Before first going from academia to industry, the author participated in a certificate program at Wharton in which PhDs from varied disciplines got a condensed version of the MBA core curriculum, much like this one, taught in one summer. That brief exposure to a different mindset was subsequently beneficial in many different contexts over the years (for instance, see the next paragraph), and a similar experience is highly recommended for those who have the time and inclination.

Textbook. Textbooks are numerous in this area and easily found. What is unique about B-school education, though, is the use of case studies. In a typical class, students will have read an extensive briefing document describing some real-life business situation. One or more of them will be called upon by the instructor in turn to provide a review of the case together with their own analysis of the situation, *e.g.,* whether or not it was handled correctly and if not, what should have been done. The instructor asks probing questions, and then sums up with a take-home message. Case studies, some of which are famous, are marketed for use in schools, notably by Harvard (http://hbsp.harvard.edu/product/cases) and Stanford (https://gsbapps.stanford.edu/cases). For instance, for a few dollars one can obtain a case study entitled *GlaxoSmithKline: Reorganizing Drug Discovery* (http://hbr.org/product/GlaxoSmithKline--Reorgani/an/605074-PDF-ENG) by Harvard faculty Robert Huckman and Eli Strick. They describe in fair detail a major reorganization that occurred at GSK, giving the industry context, the particular issues, the individuals involved, and exhibits of hard data intended to be used in the students' analyses. As someone who lived through and participated in that event as a Senior Vice President in R&D, the author can attest that the case study does indeed pick up on the significant issues dealt with at the time, and effectively simulates the same kinds of decision-making and strategy execution that had to be done in real life. The use of case studies in B-school is similar in spirit to journal clubs in biology, but tend to be more central to the core curriculum and conducted in Socratic fashion.

Going Further. MBA students also get a healthy dose of Economics and learn Entrepreneurial Skills, both of which are listed elsewhere in this Department, as well as studying Statistics, which is listed in the Mathematics Department. Coursera and edX have a wide variety of other business courses.

Writing Skills 💻

Kristin Sainani, Stanford University "Writing in the Sciences" (Coursera, Fall 2012: https://www.coursera.org/course/sciwrite or Stanford, Fall 2013: https://class.stanford.edu/courses/Medicine/SciWrite/Fall2013/about)

"This course teaches scientists to become more effective writers, using practical examples and exercises. Topics include: principles of good writing, tricks for writing faster and with less anxiety, the format of a scientific manuscript, and issues in publication and peer review."

Commentary. It goes without saying that writing is an indispensible skill for scientists of any stripe, and this course is suitably specialized. Note that many graduate programs now have courses of this sort, often geared not only to journal publication but also to grant writing, a critical skill in itself.

Personal Note. As an undergraduate, the author took both journalism and fiction-writing courses, and held several different editorial positions on the student newspaper. From a practical standpoint, no experience has been any more important than this to a subsequent career in science, in all its aspects. It

is no coincidence that successful scientists are often good writers, and that many write books directed at the general public later in their careers. Any work put into developing writing skills is sure to pay dividends.

Alternatives. Coursera has a number of more general courses in communication, including "English Composition I" by Denise Comer of Duke University (https://www.coursera.org/course/composition), "First Year Composition 2.0" by Karen Head of the Georgia Institute of Technology (https://www.coursera.org/course/gtcomp), and "Writing II: Rhetorical Composing" by a number of instructors from Ohio State University (https://www.coursera.org/course/writing2).

Gong Further. For those who are inspired to go much further and pursue a career in an area such as science journalism, see the short clip from iBioMagazine by Chris Tachibana entitled "Science Writing and Editing" (http://www.youtube.com/watch?v=5FfnnEUaLMQ).

Speaking Skills

Matt McGarrity, University of Washington COMM220UWx "Introduction to Public Speaking" (edX Winter 2014: https://www.edx.org/course/uwashingtonx/uwashingtonx-comm220uwx-introduction-1234 and Coursera, Spring 2014: https://www.coursera.org/course/publicspeak)

> *"This course is designed to help you become a more effective and confident public speaker. We will demystify the process of writing, practicing, and performing a clear and engaging speech, work through the unique traits of oral versus written communication, and learn how to prepare speeches that are easier to deliver orally and understand aurally."*

Commentary. This is one of a very few courses available on both the edX and Coursera platforms, which should make for an interesting comparison. To complete three peer-reviewed speech assignments, students make video recordings of themselves speaking.

Personal Note. As a graduate student, the author suffered crippling stage fright. If you are similarly challenged, first be assured that it *will* disappear with time and practice, but if you would like some additional advice (beyond the classic "imagine your audience in the nude") see the non-MOOC course "Overcoming Public Speaking Anxiety" by Margaret Swisher and Barbara Myslik of the University of California, Davis (https://itunes.apple.com/us/itunes-u/overcoming-public-speaking/id414117823).

Alternatives. For more specific advice, see the iBioSeminars lecture by Susan McConnell of Stanford entitled "Designing Effective Scientific Presentations" (http://www.ibiology.org/ibioseminars/susan-mcconnell-part-1.html).

Teaching Skills

Pamela Jeffries and David Andrews, Johns Hopkins University "University Teaching 101" (Coursera, Spring 2014: https://www.coursera.org/course/univteaching101)

> *"Higher education professionals, new PhD graduates, newly hired university educators and research scientists, and others interested in enhancing their teaching techniques in a university setting will acquire in this six-week course the foundational knowledge of the science of teaching and learning and develop skills and strategies for teaching at the university level."*

Commentary. As the instructors note: "Not too long ago, it was believed that anyone who graduated from a doctoral program was capable of teaching. In recent years, however, it has become apparent that teaching is not an intuitive behavior." If you are in a position to teach, now or in the future, do your prospective students a favor and give this course a look.

Alternatives. There is a welter of Coursera courses aimed at teachers, albeit mostly to do with primary education and professional development (https://www.coursera.org/courses?cats=education).

Going Further. For a more philosophical take on teaching, see "History and Future of (Mostly) Higher Education" by Duke University's Cathy Davidson (https://www.coursera.org/course/highered).

Creative Skills

Brad Hokanson and Marit McCluske, University of Minnesota "Creative Problem Solving" (Coursera, Spring 2014: https://www.coursera.org/course/cps)

> *"This course deals directly with your ability for creativity which is a critical skill in any field. It focuses on divergent thinking, the ability to develop multiple ideas and concepts to solve problems. Through a series of creativity building exercises, short lectures, and readings, learners develop both an understanding of creativity and increase their own ability."*

Commentary. This course is from the University of Minnesota College of Design and so refers to creativity in the broadest sense, rather than scientific creativity in particular.

Going Further. For a completely different take on creativity, see the 2013 "Autumn School on Computational Creativity" at the University of Helsinki (http://videolectures.net/ascc2013_porvoo). These lectures deal with a new movement that is attempting both to model creativity computationally and to enable computers "to be autonomously creative or to collaborate as co-creators with humans."

Argumentation Skills

Walter Sinnott-Armstrong and Ram Neta, Duke University "Think Again: How to Reason and Argue" (Coursera, Winter 2014: https://www.coursera.org/course/thinkagain)

> *"You will learn some simple but vital rules to follow in thinking about any topic at all and some common and tempting mistakes to avoid in reasoning. We will discuss how to identify, analyze, and evaluate arguments by other people (including politicians, used car salesmen, and teachers) and how to construct arguments of your own in order to help you decide what to believe or what to do."*

Commentary. The description makes it sound a bit like a self-help course, but actually the syllabus includes a fairly systematic catalog of deductive and inductive argumentation patterns, causal reasoning, fallacies, and so on, which should serve any scientist well.

Alternatives. The Carnegie Mellon Open Learning Initiative has a non-video self-study tutorial in "Argument Diagramming" that uses software tools as aids in constructing arguments (http://oli.cmu.edu/courses/free-open/argument-diagramming-course-details). Marianne Talbot of Oxford University teaches "Critical Reasoning: A Romp Through the Foothills of Logic" on iTunes U (https://itunes.apple.com/us/itunes-u/critical-reasoning-romp-through/id852351853).

Leadership Skills

Robert Austin, Shannon Hessel, and Richard L. Nolan, Copenhagen Business School "Leadership in 21st Century Organizations" (Coursera, TBA: https://www.coursera.org/course/leadership21st)

> *"The 21st Century Leadership Challenge… Getting your Bearings and Constituting Agendas… Assessing the Financial Context Session… Transparency, Trust, and Accountability to Stakeholders, Communication and Relationships… Accessing Expertise… Inspiring Teams and Creating Opportunity for People… Governance, Restructuring, and Risk… Execution in a Globalized, Networked World… A Framework for Leadership in the 21st Century."*

Commentary. This course is taught with an orientation toward the business context, but leadership obviously has value in organized research as well.

Personal Note. The author, having learned leadership (however imperfectly) by trial and error at nearly every level from small project management to responsibility for a corporate division, feels bound to suggest that this is likely to be the one topic in this curriculum that may not be teachable in either a virtual or real classroom. This is not to suggest that taking a course like this is completely without value, but rather that one should not expect to master the subject by studying it. Look for every opportunity to actually do it, at any scale. Start making your mistakes as soon as possible.

Alternatives. Coursera also has a more cosmopolitan "International Organizational Behavior and Leadership" course from the Università Bocconi (https://www.coursera.org/course/intorb), and other entries with a more inspirational tone such as "Inspiring Leadership through Emotional Intelligence" by Richard Boyatzis of Case Western Reserve University (https://www.coursera.org/course/lead-ei) and "Better Leader, Richer Life" by Stewart Friedman of the Wharton School of the University of Pennsylvania (https://www.coursera.org/course/totalleadership).

Going Further. The Massachusetts Institute of Technology provides "Leadership and Empowerment: Resources from Graduate Women at MIT (GWAMIT)" with videos pertinent to women (though men are welcome) (http://ocw.mit.edu/resources/res-cd-001-leadership-and-empowerment-resources-from-graduate-women-at-mit-gwamit-spring-2012).

Entrepreneurial Skills

James V. Green, University of Maryland, College Park "Developing Innovative Ideas for New Companies: The First Step in Entrepreneurship" (Coursera, Winter 2014: https://www.coursera.org/course/innovativeideas)

> *"Using proven content, methods, and models for new venture opportunity assessment and analysis, students will learn how to enhance their entrepreneurial mindset and develop their functional skill sets to see and act entrepreneurially. The initial steps to creating a business plan, and raising financial capital to launch the firm, are examined as well. Our goal is to demystify the startup process, and to help you build the skills to identify and act on innovative opportunities now, and in the future."*

Commentary. There are many MOOCs dealing with entrepreneurship, but this one is especially highly rated by former students. Unlike some of the Alternatives below, it seems to be generic as regards the type of business to be started.

Alternatives. For more specialized courses, see Healthcare Entrepreneurship in the Translational Sciences Department and Software Engineering in the Computer Science Department. For a technology orientation, try "Innovation and Commercialization" by Eugene Fitzgerald and Andreas Wankerl of the Massachusetts Institute of Technology on edX (https://www.edx.org/course/mitx/mitx-3-086x-innovation-commercialization-880). Coursera has two interesting variations: "Social Entrepreneurship" by Kai Hockerts of Copenhagen Business School (https://www.coursera.org/course/socialentrepeneur) and "Beyond Silicon Valley: Growing Entrepreneurship in Transitioning Economies" by Michael Goldberg of Case Western Reserve University (https://www.coursera.org/course/entpecon). Udacity and edX have other generic entrepreneurship courses as well, and still other resources are listed in the previous catalog.

Going Further. See the Business entry elsewhere in this department.

Humanities Seminars

(See seminars at http://www.youtube.com/playlist?list=PL9s5HIfOWP3MMOB06Sbxg-ToIDAJ3eiUs)

References

1. Klein PN (2013) Coding the Matrix: Linear Algebra through Applications to Computer Science. Newton, MA: Newtonian Press.
2. Artin M (1991) Algebra. Upper Saddle River, NJ: Pearson.
3. Rudin W (1976) Principles of Mathematical Analysis. New York: McGraw-Hill.
4. Hurdal MK, Stephenson K (2009) Discrete conformal methods for cortical brain flattening. Neuroimage 45(1 Suppl):S86-98. doi: 10.1016/j.neuroimage.2008.10.045
5. MacLennan BJ (2013) Cognition in Hilbert space. Behav Brain Sci 36(3):296-297. doi: 10.1017/S0140525X1200283X
6. Martin-Merino M, Blanco A, De Las Rivas J (2009) Combining dissimilarities in a hyper reproducing kernel Hilbert space for complex human cancer prediction. J Biomed Biotechnol 2009:906865. doi: 10.1155/2009/906865
7. Mishra R, Bhushan S (2012) Knot theory in understanding proteins. J Math Biol 65(6-7):1187-213. doi: 10.1007/s00285-011-0488-3.
8. Stanley RP (2011) Enumerative Combinatorics. Cambridge: Cambridge University Press.
9. Bertsekas DP, Tsitsiklis JN (2008) Introduction to Probability, 2nd Edition. Nashua, NH: Athena Scientific.
10. Gallier, JH (2014) Logic for Computer Science: Foundations of Automatic Theorem Proving, 2nd Edition. Mineola, NY: Dover.
11. Hopcroft A, Motwani R, Ullman J (2007) Automata Theory, Languages, and Computation, 3rd Edition. Upper Saddle River, NJ: Prentice Hall.
12. Sipser M (2012) An Introduction to the Theory of Computation, 3rd Edition. Boston: Cengage Learning.
13. Yousefi MR, Dougherty ER (2013) Intervention in gene regulatory networks with maximal phenotype alteration. Bioinformatics 29(14):1758-67. doi: 10.1093/bioinformatics/btt242
14. Chen ZZ, Deng F, Wang L (2013) Exact algorithms for haplotype assembly from whole-genome sequence data. Bioinformatics 29(16):1938-45. doi: 10.1093/bioinformatics/btt349.
15. Li Z, Wang RS, Zhang XS (2011) Two-stage flux balance analysis of metabolic networks for drug target identification. BMC Syst Biol 5 Suppl 1:S11. doi: 10.1186/1752-0509-5-S1-S11
16. Huang T, He Z (2012) A linear programming model for protein inference problem in shotgun proteomics. Bioinformatics 28(22):2956-62. doi: 10.1093/bioinformatics/bts540
17. Kutz N (2013) Data-Driven Modeling & Scientific Computation. Oxford: Oxford University Press.
18. Alligood KT, Sauer TD, Yorke JA (2000) CHAOS: An Introduction to Dynamical Systems. New York: Springer.
19. Pal R, Huang Y, Chen Y (2012) Selected articles from the IEEE International Workshop on Genomic Signal Processing and Statistics (GENSIPS'2011). BMC Genomics 13 Suppl 6:S1. doi: 10.1186/1471-2164-13-S6-S1
20. Kumar V, Muratani M, Rayan NA, Kraus P, Lufkin T, Ng HH, Prabhakar S (2013) Uniform, optimal signal processing of mapped deep-sequencing data. Nat Biotechnol 31(7):615-22. doi: 10.1038/nbt.2596
21. Han Z, Tian L, Pécot T, Huang T, Machiraju R, Huang K (2012) A signal processing approach for enriched region detection in RNA polymerase II ChIP-seq data. BMC Bioinformatics 13 Suppl 2:S2. doi: 10.1186/1471-2105-13-S2-S2

22. Cheever EA, Overton GC, Searls DB (1991) Fast Fourier transform-based correlation of DNA sequences using complex plane encoding. Comput Appl Biosci 7(2):143-54.

23. Adami C (2012) The use of information theory in evolutionary biology. Ann N Y Acad Sci 1256:49-65. doi: 10.1111/j.1749-6632.2011.06422.x.

24. Waltermann C, Klipp E (2011) Information theory based approaches to cellular signaling. Biochim Biophys Acta 1810(10):924-32. doi: 10.1016/j.bbagen.2011.07.009.

25. Yeung RW (2010) Information Theory and Network Coding. New York: Springer.

26. Leyton-Brown K, Shoham Y (2008) Essentials of Game Theory: A Concise Multidisciplinary Introduction. San Rafael, CA: Morgan and Claypool.

27. Diestel R (2010) Graph Theory, 4th Edition. New York: Springer.

28. Guttag JV (2013) Introduction to Computation and Programming Using Python, Revised and Expanded edition,. Cambridge, MA: MIT Press.

29. Sedgewick R, Wayne K (2014) Algorithms: Part 1 (4th Edition). Boston: Addison-Wesley.

30. Bryant RE, O'Hallaron DR (2010) Computer Systems: A Programmer's Perspective. Boston: Addison-Wesley.

31. Bartlett J (2004) Programming from the Ground Up. Broken Arrow, OK: Bartlett Publishing.

32. Cormen TH, Leiserson CE, Rivest RL, Stein C (2009) Introduction to Algorithms 3rd Edition. Cambridge, MA: MIT Press.

33. Silberschatz A, Galvin PB, Gagne G (2012) Operating Systems Concepts, 9th Edition. New York: Wiley.

34. Yiu CP, Chen YW (2014) High-quality macromolecular graphics on mobile devices: a quick starter's guide. Methods Mol Biol 1091:343-52. doi: 10.1007/978-1-62703-691-7_24.

35. Franckle T, Haas D, Mandl KD (2013) App Store for EHRs and Patients Both. AMIA Summits Transl Sci Proc. 2013:73.

36. Clark AM, Ekins S, Williams AJ (2012) Redefining Cheminformatics with Intuitive Collaborative Mobile Apps. Mol Inform 31(8):569-584.

37. Bentley F, Barrett E (2012) Building Mobile Experiences. Cambridge, MA: MIT Press.

38. Valvano JW (2012) Embedded Systems: Introduction to Arm® Cortex(TM)-M Microcontrollers. CreateSpace Independent Publishing Platform.

39. Chen Y, Schmidt B, Maskell DL (2013) A hybrid short read mapping accelerator. BMC Bioinformatics 14:67. doi: 10.1186/1471-2105-14-67.

40. Lloyd S, Snell QO (2011) Accelerated large-scale multiple sequence alignment. BMC Bioinformatics. 12:466. doi: 10.1186/1471-2105-12-466.

41. Zierke S, Bakos JD (2010) FPGA Acceleration of the phylogenetic likelihood function for Bayesian MCMC inference methods. BMC Bioinformatics. 11:184. doi: 10.1186/1471-2105-11-184.

42. Kirk DB, Hwu WW (2012) Programming Massively Parallel Processors, 2nd Edition: A Hands-on Approach. Amsterdam: Morgan Kaufman.

43. Odersky M, Spoon L, Venners B (2010) Programming in Scala, 2nd Edition. Walnut Creek, CA: Artima Press.

44. Tanenbaum AS, Wetherall DJ (2010) Computer Networks, 5th Edition. Englewood Cliffs, NJ: Prentice Hall.

45. Gibson TA (2012) The roots of bioinformatics in ISMB. PLoS Comput Biol 8(8): e1002679. doi:10.1371/journal.pcbi.1002679

46. Russell S, Norvig P (2009) Artificial Intelligence: A Modern Approach, 3rd Edition. Englewood Cliffs, NJ: Prentice Hall.

47. Bird S, Klein E, Loper E (2009) Natural Language Processing with Python. Sebastopol, CA: O'Reilly Media.

48. Manjasetty BA, Turnbull AP, Panjikar S, Büssow K, Chance MR (2008) Automated technologies and novel techniques to accelerate protein crystallography for structural genomics. Proteomics 8(4):612-625. doi: 10.1002/pmic.200700687.

49. Gaisford W (2012) Robotic liquid handling and automation in epigenetics. J Lab Autom. 17(5):327-329.

50. Mura C, McCrimmon CM, Vertrees J, Sawaya MR (2010) An Introduction to Biomolecular Graphics. PLoS Comput Biol 6(8): e1000918. doi: 10.1371/journal.pcbi.1000918.

51. Bromberg S, Chiu W, Ferrin TE (2010) Workshop on Molecular Animation. Structure 18(10):1261–1265. doi: 10.1016/j.str.2010.09.001.

52. Shirley P, Ashikhmin M, Marschner S (2009) Fundamentals of Computer Graphics. Boca Raton, FL: AK Peters/CRC Press.

53. Gonzalez RC, Woods RE (2007) Digital Image Processing, 3rd Edition. Englewood Cliffs, NJ: Prentice Hall.

54. Ullman J, Rajaraman A (2013) Mining of Massive Datasets. Cambridge UK: Cambridge University Press. 326 p.

55. Manning C, Raghavan P, Schutze H (2008) Introduction to Information Retrieval. Cambridge UK: Cambridge University Press. 496 p.

56. Baca M (2008) Introduction to Metadata. Los Angeles: Getty Research Institute.

57. James G, Witten D, Hastie T, Tibshirani R (2013) An Introduction to Statistical Learning: with Applications in R. New York: Springer.

58. Hastie T, Tibshirani R, Friedman J (2009) The Elements of Statistical Learning: Data Mining, Inference, and Prediction (2nd edition). New York: Springer.

59. Koller D (2009) Probabilistic Graphical Models: Principles and Techniques. Cambridge, MA: MIT Press.

60. McQuarie DA, Simon JD (1997) Physical Chemistry: A Molecular Approach. Sausalito, CA: University Science Books.

61. Averill B, Eldredge P (2011) General Chemistry: Principles, Patterns, and Applications. Washington DC: Flat World Knowledge.

62. Rupp B (2009) Biomolecular Crystallography: Principles, Practice, and Application to Structural Biology. New York: Garland Science.

63. Phan JH, Moffitt RA, Stokes TH, Liu J, Young AN, Nie S, Wang MD (2009) Convergence of biomarkers, bioinformatics and nanotechnology for individualized cancer treatment. Trends Biotechnol. 27(6):350-358. doi: 10.1016/j.tibtech.2009.02.010.

64. Nelson DL, Cox MM (2012) Lehninger Principles of Biochemistry, 6th Edition. New York: W.H. Freeman.

65. Redfield RJ (2012) "Why do we have to learn this stuff?"—A new genetics for 21st century students. PLoS Biol 10(7): e1001356.

66. Alberts B, Johnson A, Lewis J, Raff M, Roberts K, Walter P (2007) The Molecular Biology of the Cell. New York: Garland Science.

67. Reinberg D, Jenuwein T (2008) Epigenetics. Cold Spring Harbor, NY: Cold Spring Harbor Laboratory Press.
68. Flint SJ, Enquist LW, Racaniello VR, Skalka AM (2008). Principles of Virology. Washington, DC: ASM Press.
69. Zvelebil M, Baum JO (2007) Understanding Bioinformatics. New York: Garland Science.
70. Lesk A (2014) Introduction to Bioinformatics. Oxford: Oxford University Press.
71. Felsenstein J (2003) Inferring Phylogenies. Sunderland, MA: Sinauer.
72. Jones NC, Pevzner PA (2004) An Introduction to Bioinformatics Algorithms. Cambridge, MA: MIT Press.
73. Stearns SC, Hoekstra R (2005) Evolution: An Introduction. Oxford: Oxford University Press.
74. Shapiro B, Hofreiter M (2014) A paleogenomic perspective on evolution and gene function: new insights from ancient DNA. Science 343, 1236573. doi: 10.1126/science.1236573.
75. Alon U (2006) An Introduction to Systems Biology: Design Principles of Biological Circuits. Boca Raton, FL: Chapman & Hall/CRC.
76. Easley D, Kleinberg J (2010) Networks, Crowds and Markets: Reasoning about a Highly Connected World. Cambridge, UK: Cambridge University Press.
77. Jackson M (2009) Social and Economic Networks. Princeton, NJ: Princeton University Press.
78. Miller JH, Page SE (2009) Complex Adaptive Systems: An Introduction to Computational Models of Social Life. Princeton, NJ: Princeton University Press.
79. Lettvin, JL, Maturana HR, McCulloch WS, Pitts WH (1959) What the Frog's Eye Tells the Frog's Brain. Proc Inst Radio Engr 47:1940-1951.
80. Nicholls JG, Martin AR, Fuchs PA, Brown DA, Diamond ME, Weisblat D (2011) From Neuron to Brain, 5th Edition. Sunderland, MA: Sinauer.
81. Mason P (2011) Medical Neurobiology. Oxford, UK: Oxford University Press.
82. Purves D, Augustine GJ, Fitzpatrick D, Hall WC, LaMantia A-S, White LE (2011) Neuroscience, Fifth Edition. Sunderland, MA: Sinauer Associates.
83. Groh JM (2014) Making Space: How the Brain Knows Where Things Are. Cambridge, MA: Belknap Press of Harvard University Press.
84. Agarwal A, Lang JH (2005) The Foundations of Analog and Digital Electronic Circuits. San Francisco: Morgan Kaufman.
85. Dayan P, Abbott LF (2005) Theoretical Neuroscience: Computational and Mathematical Modeling of Neural Systems. Cambridge, MA: MIT Press.
86. Gerstner W, Kistler WM, Naud R, Paninski, L (2014) Neuronal Dynamics: From Single Neurons to Networks and Models of Cognition. Cambridge, UK: Cambridge University Press.
87. Gerstner W, Kistler WM (2002) Spiking Neuron Models: Single Neurons, Populations, Plasticity. Cambridge: Cambridge University Press.
88. Glimcher PW, Fehr E (2013) Neuroeconomics: Decision Making and the Brain, 2nd Edition. Burlington, MA: Academic Press.
89. Glimcher PW (2003) Decisions, Uncertainty, and the Brain: The Science of Neuroeconomics. Cambridge, MA: MIT Press.
90. Revonsuo A (2009) Consciousness: The Science of Subjectivity. New York: Psychology Press.

91. Nestler E, Hyman S, Malenka R (2008) Molecular Neuropharmacology: A Foundation for Clinical Neuroscience. New York: McGraw-Hill.
92. Braunstein ML (2012) Health Informatics in the Cloud. New York: Springer.
93. Pagano M, Gavreau K (2000) Principles of Biostatistics. Independence, KY: Cengage Learning.
94. Friedman LM, Furberg CD, DeMets DL (2010) Fundamentals of Clinical Trials. New York: Springer.
95. McDonough JE (2011) Inside National Health Reform. Berkeley, CA: University of California Press.
96. Searls DB (2009) Omic empiricism. Sci Signal 2(68):eg6. doi: 10.1126/scisignal.268eg6.
97. Sandel MJ (2009) Justice: What's the Right Thing to Do? New York: Farrar, Straus and Giroux.
98. Searle JR (1990) The Mystery of Consciousness. New York: The New York Review of Books.
99. Lodge D (2002) Thinks… London: Penguin Books.
100. Lodge D (2004) Consciousness and the Novel: Connected Essays. Cambridge, MA: Harvard University Press.
101. Searls DB (1972) Will robots marry? Technol Rev 74(5):8-9.
102. Falk D (2014) The Science of Shakespeare: A New Look at the Playwright's Universe. New York: Thomas Dunne Books.
103. Altschuler EL, Calude AS, Meade A, Pagel M (2013) Linguistic evidence supports date for Homeric epics. Bioessays 35(5):417-20. doi: 10.1002/bies.201200165.
104. Turchin P (2008) Arise "cliodynamics." Nature 454(7200):34-35. doi: 10.1038/454034a.
105. Diamond JM and Ordunio D (1997) Guns, Germs, and Steel. New York: Norton.
106. Daly P, Evans TL (2006) Digital Archaeology: Bridging Method and Theory. New York: Routledge.
107. Parcak SH (2009) Satellite Remote Sensing for Archaeology. New York: Routledge.
108. Hart JP, Terrell JE, eds. (2002) Darwin and Archaeology: A Handbook of Key Concepts. Westport, CT: Bergin & Garvey.
109. Renfrew C, Bahn P (2012) Archaeology: Theories, Methods, and Practice, 6th Edition. London: Thames & Hudson.
110. Gould SJ, Lewontin RC (1979) The spandrels of San Marco and the Panglossian paradigm: a critique of the adaptationist programme. Proc. Roy. Soc. London B 205:581-598.
111. Mark R (1996) Architecture and evolution. American Scientist (July-August):383-389.
112. Jarzombek MM, Prakash V, Ching FDK (2010) A Global History of Architecture. Hoboken, NJ: Wiley.
113. Zaidel DW (2013) Art and brain: the relationship of biology and evolution to art. Prog Brain Res. 204:217-33. doi: 10.1016/B978-0-444-63287-6.00011-7.
114. Cooper S, Khatib F, Treuille A, Barbero J, Lee J, Beenen M, Leaver-Fay A, Baker D, Popović Z, Players F (2010) Predicting protein structures with a multiplayer online game. Nature 466(7307):756-60. doi: 10.1038/nature09304.
115. Eiben CB, Siegel JB, Bale JB, Cooper S, Khatib F, Shen BW, Players F, Stoddard BL, Popovic Z, Baker D (2012) Increased Diels-Alderase activity through backbone remodeling guided by Foldit players. Nat Biotechnol 30(2):190-2. doi: 10.1038/nbt.2109.
116. Fitch WT (2006) The biology and evolution of music: a comparative perspective. Cognition 100: 173-215.

117. Bradley DW, Bradley RA (1983) Application of sequence comparison to the study of bird songs. In Time Warps, String Edits, and Macromolecules: The Theory and Practice of Sequence Comparison, Sankoff D and Kruskal J, eds., 189-209. Chicago: University of Chicago Press.

118. Daou A, Johnson F, Wu W, Bertram R. (2012) A computational tool for automated large-scale analysis and measurement of bird-song syntax. J Neurosci Methods. 210(2):147-60. doi: 10.1016/j.jneumeth.2012.07.020

119. Lovell PV, Clayton DF, Replogle KL, Mello CV (2008) Birdsong "transcriptomics": neurochemical specializations of the oscine song system. PLoS One 3(10):e3440. doi: 10.1371/journal.pone.0003440

120. Scharff C, Haesler S (2005) An evolutionary perspective on FoxP2: strictly for the birds? Curr Opin Neurobiol. 15(6):694-703.

121. Kostka S, Payne D (2012) Tonal Harmony. Boston: McGraw-Hill.

122. Rigoux L, Guigon E (2012) A model of reward- and effort-based optimal decision making and motor control. PLoS Comput Biol8(10):e1002716. doi: 10.1371/journal.pcbi.1002716

123. Friesen ML, Jones EI (2012) Modelling the evolution of mutualistic symbioses. Methods Mol Biol. 2012;804:481-99. doi: 10.1007/978-1-61779-361-5_24.
